# Supplementary material for: Discovery of a peripheral 5HT2A antagonist as a clinical candidate for metabolic dysfunction-associated steatohepatitis
Source: Nat Commun. 2024 Jan 20;15:645. doi: 10.1038/s41467-024-44874-3 (PMC10799935; doi:10.1038/s41467-024-44874-3)
Supplement: Supplementary file 1 — Supplementary Information [file 41467_2024_44874_MOESM1_ESM.pdf]

Supporting Information

Discovery of a peripheral $5HT_{2A}$ antagonist as a clinical candidate for Metabolic Dysfunction-Associated Steatohepatitis

Haushabhau S. Pagire[1,2,‡], Suvarna H. Pagire[1,2,‡], Byung-kwan Jeong[3,‡], Won-Il Choi[3, ‡], Chang Joo Oh[4], Chae Won Lim[5], Minhee Kim[1], Jihyeon Yoon[1], Seong Soon Kim[6], Myung Ae Bae[6], Jae-Han Jeon[4,7], Sungmin Song[2], Hee Jong Lee[2], Eun Young Lee[2], Peter C. Goughnour[2], Dooseop Kim[2], In-Kyu Lee[4,8], Rohit Loomba[9], Hail Kim[3,10,*], Jin Hee Ahn[1,2,*]

[1] Department of Chemistry, Gwangju Institute of Science and Technology, Gwangju 61005, Republic of Korea
[2] JD Bioscience Inc., TJS Knowledge Industrial Center Suite 801, 208 Beon-gil Cheomdangwagi-ro, Buk-gu, Gwangju 61011, Republic of Korea
[3] Graduate School of Medical Science and Engineering, Korea Advanced Institute of Science and Technology, Daejeon 34141, Republic of Korea
[4] Research Institute of Aging and Metabolism, Kyungpook National University School of Medicine, Daegu 41404, Republic of Korea
[5] Leading-edge Research Center for Drug Discovery and Development for Diabetes and Metabolic Disease, Kyungpook National University Hospital, Daegu 41404, Republic of Korea
[6] Bio & Drug Discovery Division, Korea Research Institute of Chemical Technology, Daejeon 34114, Republic of Korea.
[7] Department of Internal Medicine, School of Medicine, Kyungpook National University, Kyungpook National University Chilgok Hospital, Daegu 41404, Republic of Korea
[8] Department of Internal Medicine, School of Medicine, Kyungpook National University, Kyungpook National University Hospital, Daegu 41944, Republic of Korea
[9] NAFLD Research Center, Division of Gastroenterology and Hepatology, Department of Medicine, University of California at San Diego, La Jolla, CA 92093, USA.
[10] Biomedical Research Center, Korea Advanced Institute of Science and Technology (KAIST), Daejeon, 34141, Republic of Korea

[‡]These authors equally contributed to this work.

*To whom correspondence should be addressed:

Supplementary Tables

Supplementary Table 1. Experimental conditions for the 5HT subtypes

Antagonism assay	Source	Stimulus	Incubation	Measured Component	Detection Method
$5HT_{1A}$ (h)	human recombinant (BA/F3 cells)	serotonin (15 nM)	RT	intracellular $[Ca^{2+}]$	Fluorimetry
$5HT_{1B}$ (h)	human recombinant (Hela cells)	serotonin (100 nM)	20 min., 37°C	cAMP	HTRF
$5HT_{1D}$	rat recombinant (CHO cells)	serotonin (3 nM)	28°C	impedance	Cellular dielectric spectroscopy
$5HT_{2B}$ (h)	human recombinant (CHO cells)	serotonin (30 nM)	30 min., 37°C	IP1	HTRF
$5HT_{2C}$ (h)	human recombinant (HEK-293 cells)	serotonin (10 nM)	30 min., 37°C	IP1	HTRF
$5HT_{4E}$ (h)	human recombinant (CHO cells)	serotonin (30 nM)	30 min., RT	cAMP	HTRF
$5HT_{6}$ (h)	human recombinant (CHO cells)	serotonin (300 nM)	30 min., 37°C	cAMP	HTRF
$5HT_{7}$ (h)	human recombinant (CHO cells)	serotonin (300 nM)	30 min., 37°C	cAMP	HTRF

Supplementary Table 2. Primer sequences

Gene name	Forward primer (5’ to 3’)	Reverse primer (5’ to 3’)
Htr2a	CGTGTCCATGTTAACCATCCT	ACTGGGATTGGCATGGATATAC
Srebp1c	ATCGCAAACAAGCTGACCTG	AGATCCAGGTTTGAGGTGGG
Fasn	AAGCGGTCTGGAAAGCTGAA	AGGCTGGGTTGATACCTCCA
Pparγ	CATTTCTGCTCCACACTATGAAG	CATCTTGGACGTAGAGGTGGA
36b4	GAGGAATCAGATGAGGATATGGGA	AAGCAGGCTGACTTGGTTGC

Supplementary Figures

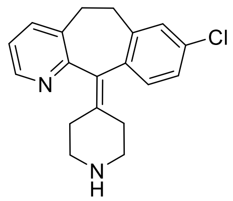

Desloratadine (2)
Brand Name: Clarinex

Supplementary Figure 1: Structure of compound 2.

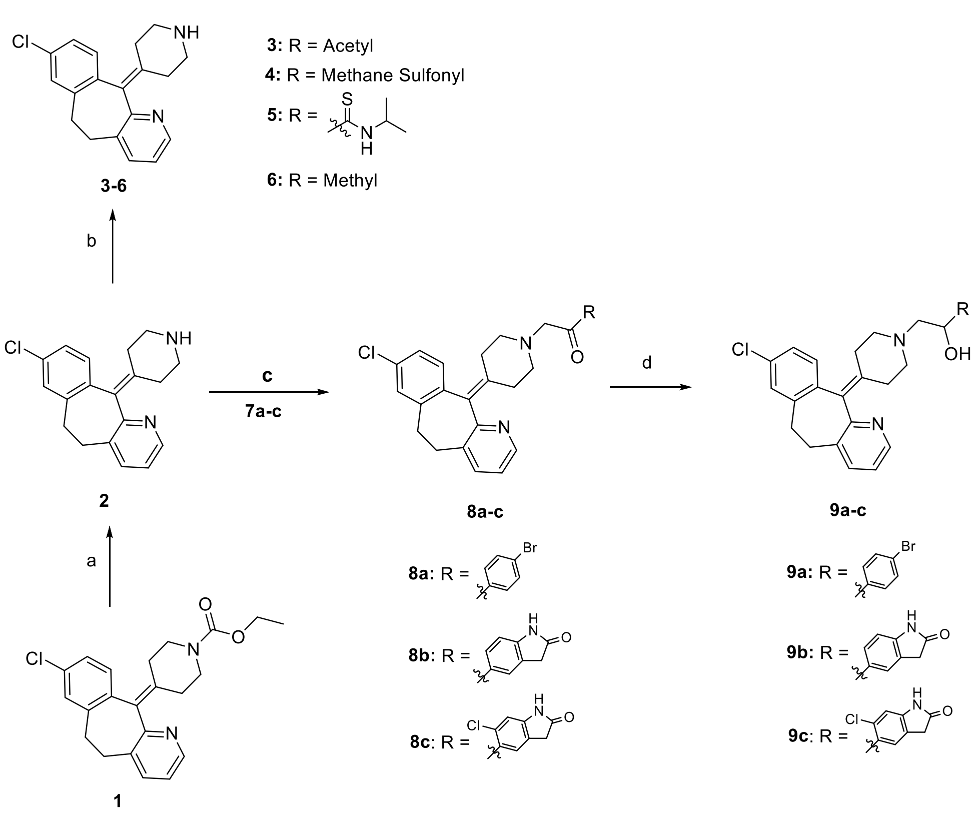

Supplementary Figure 2: Synthesis of compounds 2-6, 8a-c and 9a-c. Reagents and conditions: (a) conc. HCl, reflux, 12 h, 96 %; (b) (i) Acetyl chloride or methane sulfonyl chloride, TEA, DCM, 80-85 %; (ii) Isopropyl isothiocyanate, DIPEA, DCM, 90 %; (iii) formaldehyde, MeOH, $NaBH_4$, 75 %; (c) Aryl and bicyclic acetyl chloride **7a-c**, Na_2CO_3, KI, DMF, 80 ^{0}C, 80-85 %; (d) $NaBH_4$, MeOH, 70-75 %.

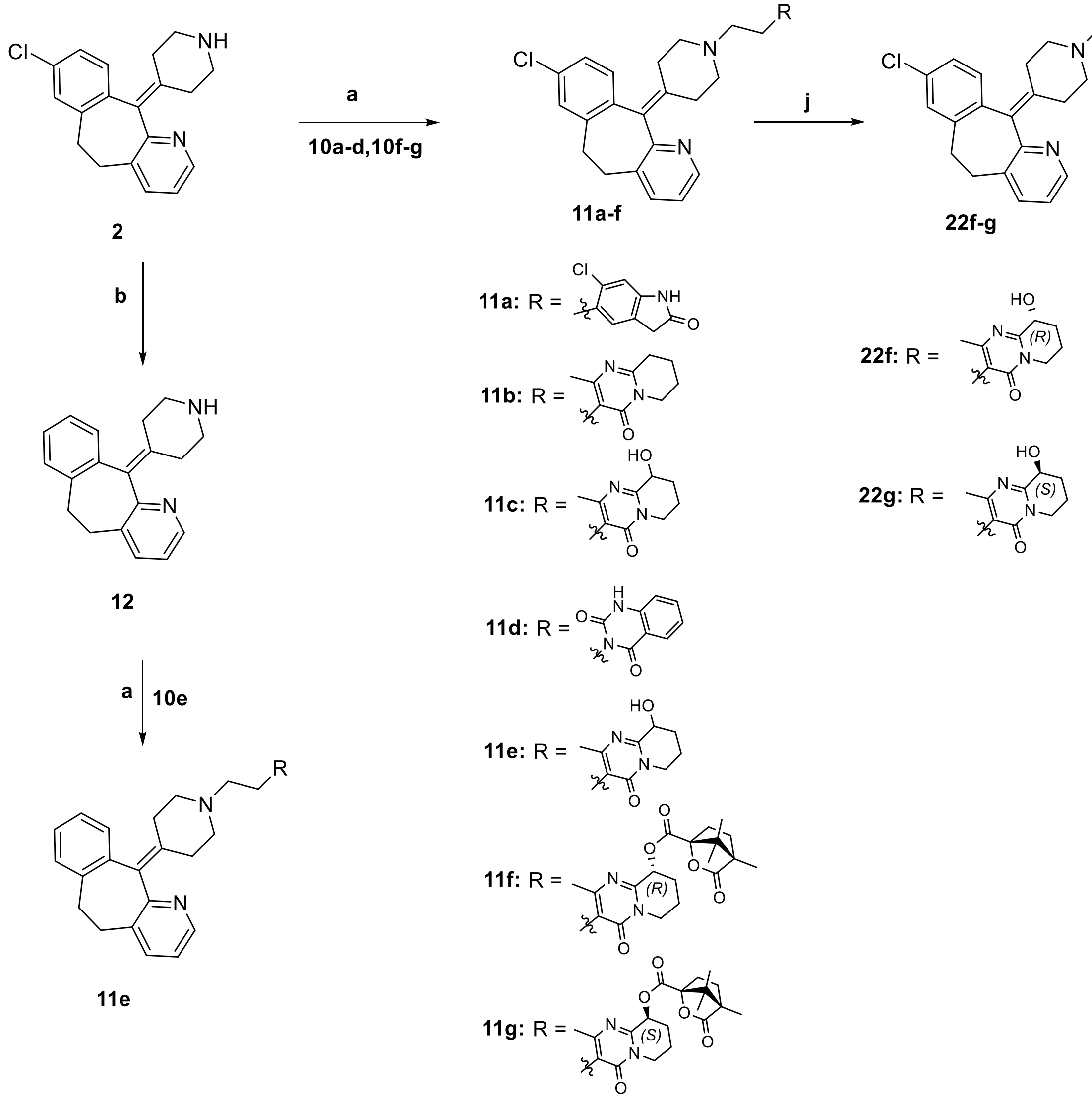

Cl
NH
N
2
b
a
10a-d,10f-g
R
Cl
N
N
11a-f
j
R
Cl
N
N
22f-g
12
a
10e
11e
11a: R =
11b: R =
11c: R =
11d: R =
11e: R =
11f: R =
11g: R =
22f: R =
22g: R =
HO
(R)
(S)

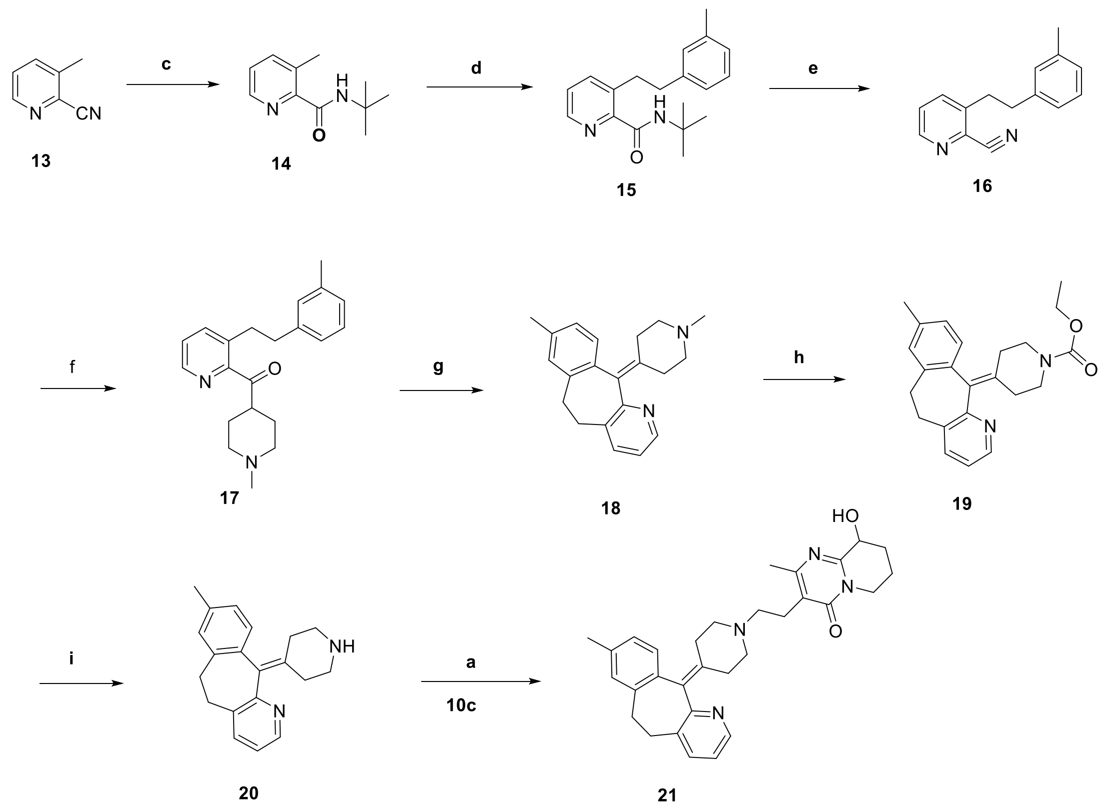

Supplementary Figure3. Synthesis of Compounds 11a-e, 22f, g and 21a,b. Reagents and conditions: (a) Bicyclic ethyl chloride, Na_2CO_3, KI, DMF, 80 ^{0}C, 80 %; (b) Pd/C, MeOH, 68 %; (c) tert-Butyl acetate, AcOH, H_2SO_4, 92%; (d) 1-(Chloromethyl)-3-methylbenzene, n-BuLi, NaBr, THF, -40 ^{0}C, 70 %; (e) $POCl_3$, reflux, 65 %; (f) 4-Chloro-1-methylpiperidine, Mg, I_2, THF, aq. HCl, MeOH, 90 %; (g) CF_3SO_3H, 95 ^{o}C, 80 %; (h) Ethyl chloroformate, toluene, DIPEA, reflux, 65 %; (i) NaOH, H_2O, EtOH, 105 ^{o}C, 5 h, 95 %; (j) *i*Pr_2NH, MeOH 6h, 98%.

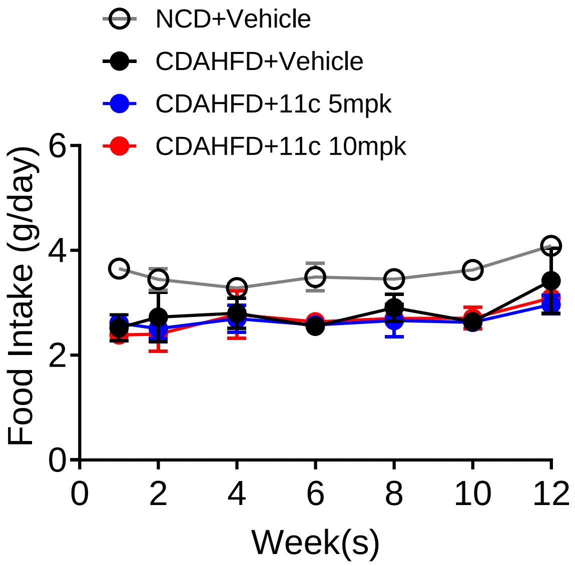

Supplementary Figure 4. Food intake. Food intake of vehicle and CDAHFD-fed mice, two-way ANOVA with the Holm-Sidak *post hoc* test. The data are presented as mean ± SD (n = 2 cages per group).

2

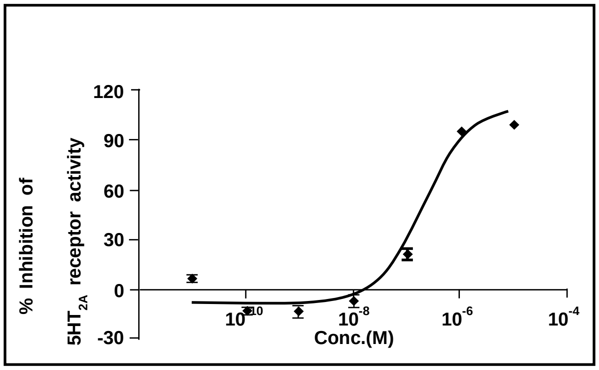
120
90
60
30
0
-30
% Inhibition of
5HT2A receptor activity
10-10
10-8
10-6
10-4
Conc.(M)

6

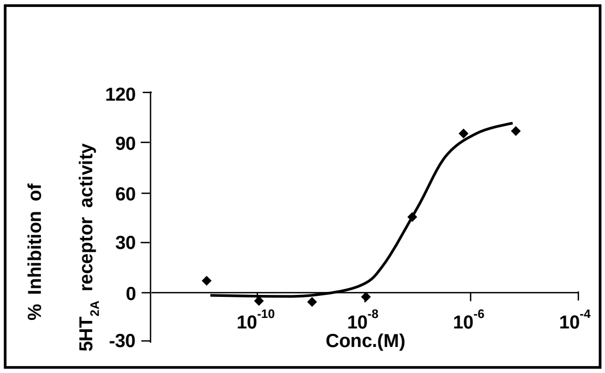
120
90
60
30
0
-30
% Inhibition of
5HT2A receptor activity
10-10
10-8
10-6
10-4
Conc.(M)

8a

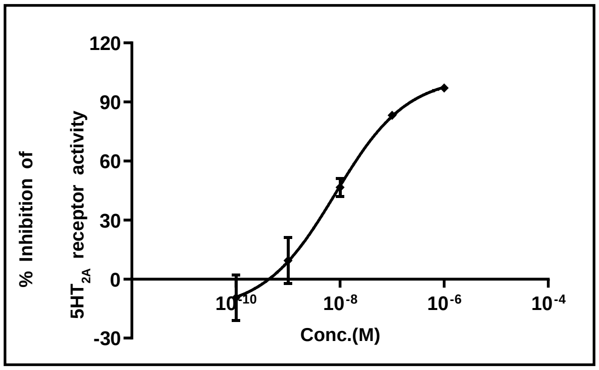
120
90
60
30
0
-30
% Inhibition of
5HT2A receptor activity
10-10
10-8
10-6
10-4
Conc.(M)

9a

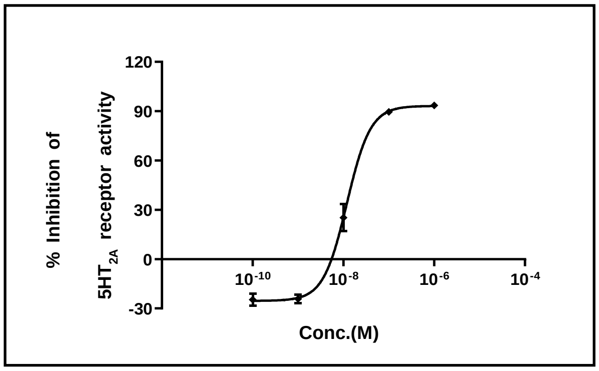
120
90
60
30
0
-30
% Inhibition of
5HT2A receptor activity
10-10
10-8
10-6
10-4
Conc.(M)

9b

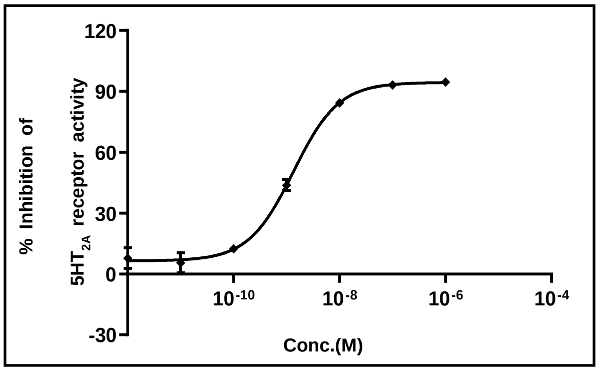
120
90
60
30
0
-30
% Inhibition of
5HT2A receptor activity
10-10
10-8
10-6
10-4
Conc.(M)

9c

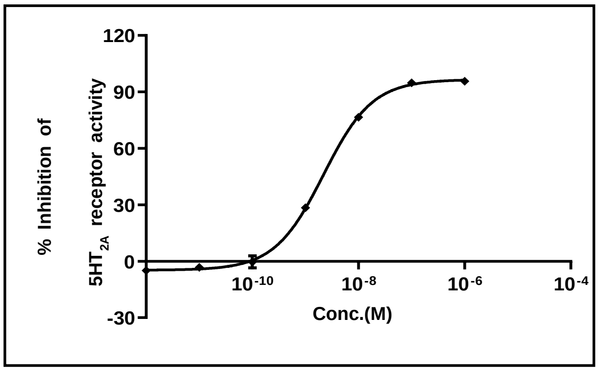
120
90
60
30
0
-30
% Inhibition of
5HT2A receptor activity
10-10
10-8
10-6
10-4
Conc.(M)

11b

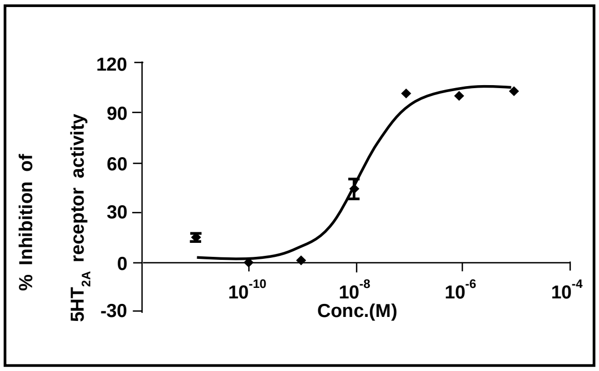

11c

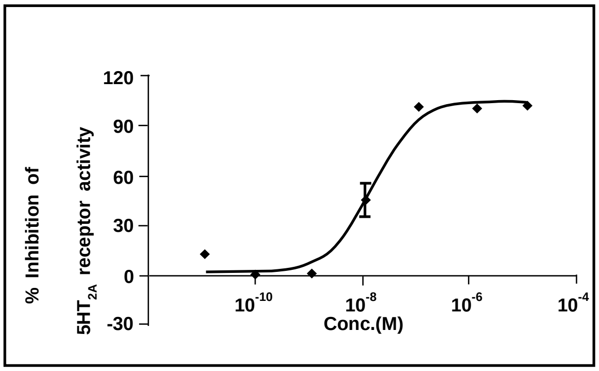

11d

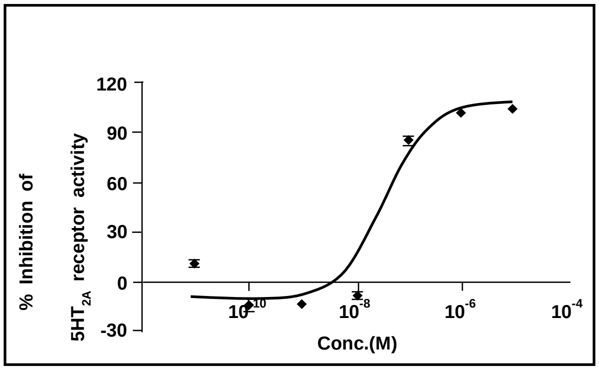

11e

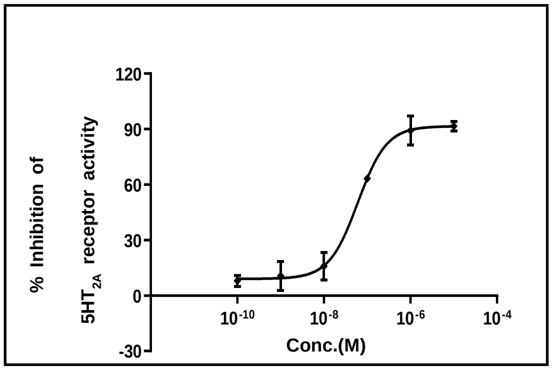

21

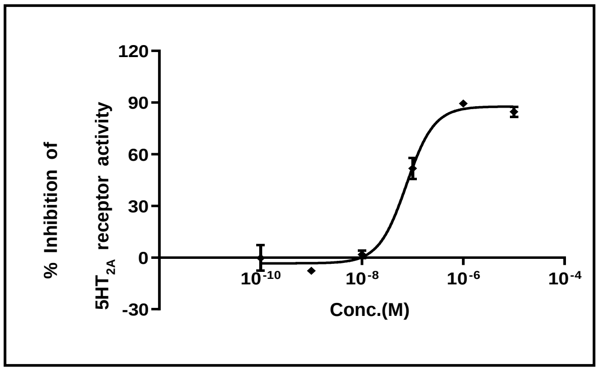

22f

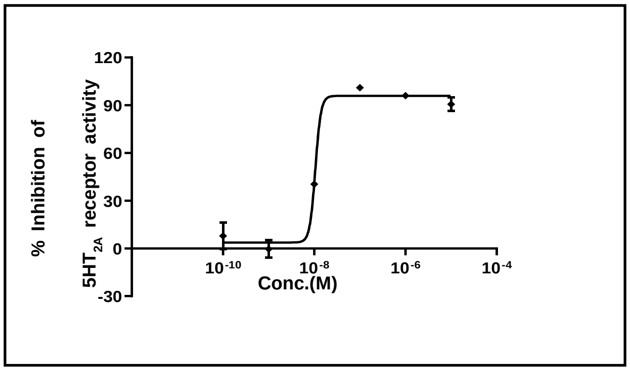

22g

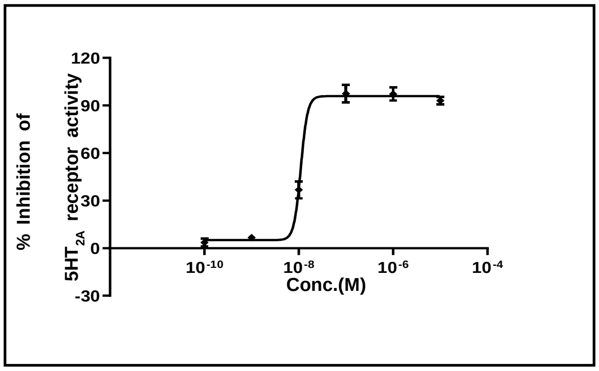

Supplementary Figure 5. Dose response curves of compounds 2, 6, 8a, 9a, 9b, 9c, 11b, 11c, 11d, 11e, 21, 22f and 22g. The X values in the dose-response dataset underwent a logarithmic transformation (X = Log(X)) using GraphPad Prism 7.04 (GraphPad, USA). Subsequently, the Y values in the dose-response data were subjected to normalization. The data are presented as mean ± SEM from 3 independent experiments.

LC purity 99.8861 %

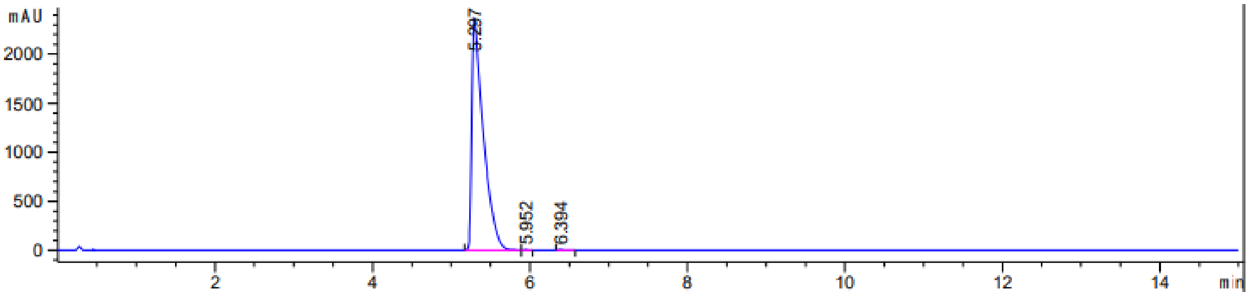

Signal 1: DAD1 A, Sig=254,4 Ref=360,100

Peak #	RetTime [min]	Type	Width [min]	Area [mAU*s]	Height [mAU]	Area %
1	5.297	BB	0.1374	2.38010e4	2365.06055	99.8861
2	5.952	BB	0.0555	6.85116	1.92562	0.0288
3	6.394	BB	0.0572	20.28279	5.23937	0.0851

Supplementary Figure 6. HPLC data of 8-chloro-11-(piperidin-4-ylidene)-6,11-dihydro-5H-benzo[5,6]cyclohepta[1,2-b]pyridine (2)

HPLC purity 98.6643 %

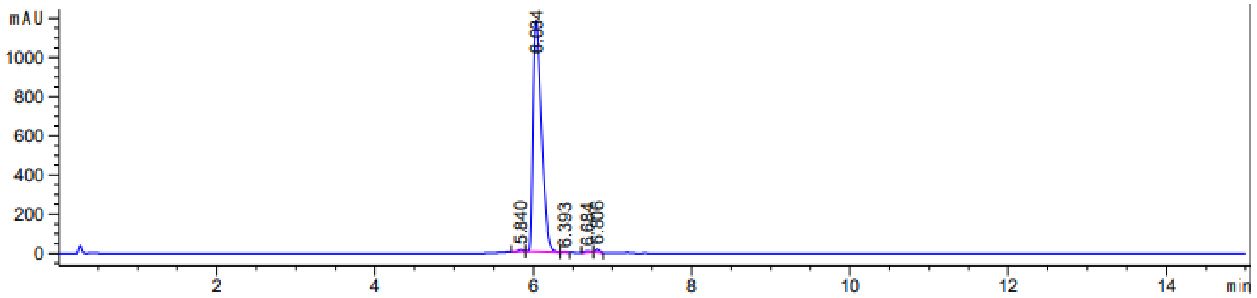

Signal 1: DAD1 A, Sig=254,4 Ref=360,100

Peak #	RetTime [min]	Type	Width [min]	Area [mAU*s]	Height [mAU]	Area %
1	5.840	BB	0.0510	27.16064	8.56277	0.3133
2	6.034	BB	0.1087	8552.38770	1178.01953	98.6643
3	6.393	BB	0.0524	10.52336	3.19899	0.1214
4	6.684	BB	0.0827	27.78792	5.92343	0.3206
5	6.806	BB	0.0498	50.30792	15.51992	0.5804

Supplementary Figure 7. HPLC data of 1-(4-(8-chloro-5,6-dihydro-11H-benzo[5,6]cyclohepta-[1,2-b]pyridin-11-ylidene)piperidin-1-yl)ethan-1-one (3)

HPLC purity 98.7862 %

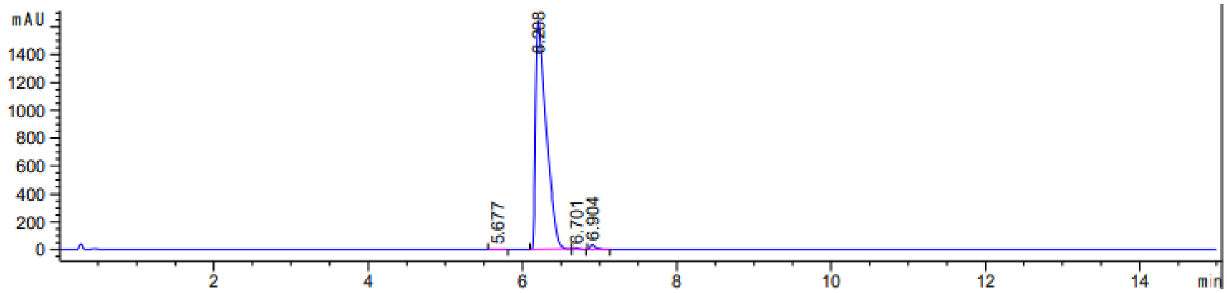

Signal 1: DAD1 A, Sig=254,4 Ref=360,100

Peak #	RetTime [min]	Type	Width [min]	Area [mAU*s]	Height [mAU]	Area %
1	5.677	BB	0.0855	6.36361	1.01053	0.0402
2	6.208	BB	0.1357	1.56514e4	1634.16589	98.7862
3	6.701	BB	0.0525	27.99076	8.47626	0.1767
4	6.904	BB	0.0675	157.95091	34.42237	0.9969

Supplementary Figure 8. HPLC data of 8-chloro-11-(1-(methylsulfonyl)piperidin-4-ylidene)-6,11-dihydro-5H-benzo[5,6] cyclohepta[1,2-b]pyridine (4)

HPLC purity 97.2193 %

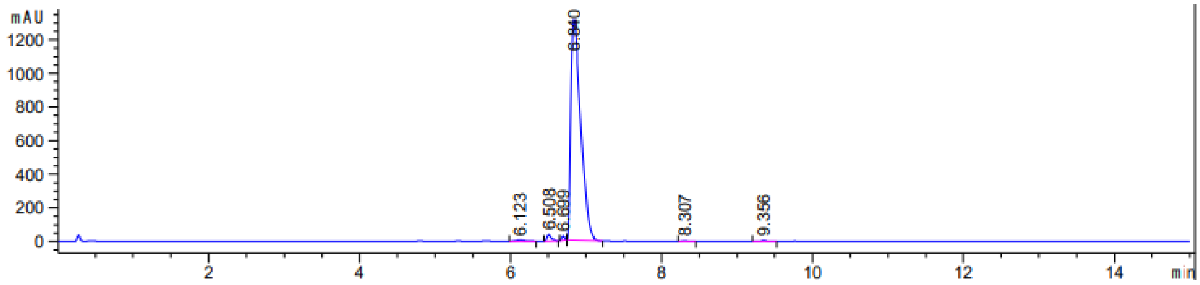

Signal 1: DAD1 A, Sig=254,4 Ref=360,100

Peak #	RetTime [min]	Type	Width [min]	Area [mAU*s]	Height [mAU]	Area %
1	6.123	BB	0.1068	75.78793	9.73811	0.6105
2	6.508	BB	0.0615	159.43781	39.13587	1.2842
3	6.699	BB	0.0463	71.79511	25.94857	0.5783
4	6.840	BB	0.1374	1.20697e4	1311.59558	97.2193
5	8.307	BB	0.0539	14.96001	4.16108	0.1205
6	9.356	BB	0.0496	23.23598	7.21901	0.1872

Supplementary Figure 9. HPLC data of 4-(8-chloro-5,6-dihydro-11H-benzo[5,6]cyclohepta[1,2-b]pyridin-11-ylidene)-N-isopropylpiperidine-1-carbothioamide (5)

HPLC purity 96.5752 %

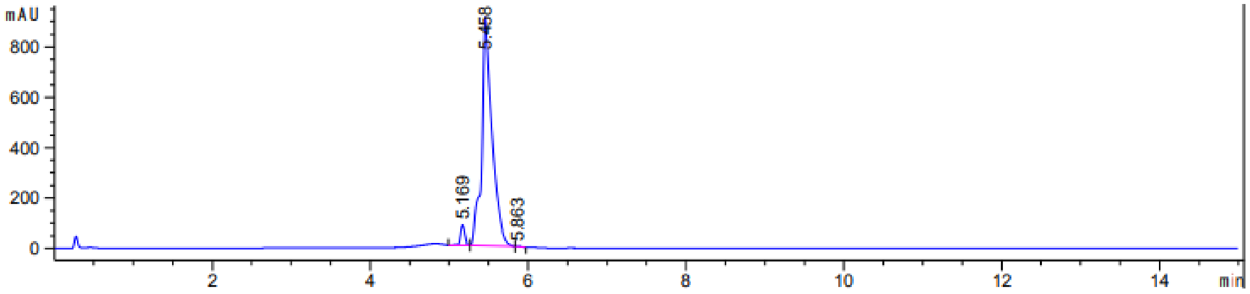


```
Signal 1: DAD1 A, Sig=254,4 Ref=360,100

Peak RetTime Type  Width      Area       Height     Area
  #   [min]        [min]    [mAU*s]      [mAU]       %
----|-------|----|-------|----------|----------|--------|
   1   5.169 BB    0.0563  292.51801   80.58737   3.3613
   2   5.458 BB    0.1258 8404.47852  909.25378  96.5752
   3   5.863 BB    0.0588    5.52908    1.44063   0.0635
```

Supplementary Figure 10. HPLC data of 8-chloro-11-(1-methylpiperidin-4-ylidene)-6,11-dihydro-5H-benzo[5,6]cyclohepta[1,2-b]pyridine (6)

HPLC purity 99.0088 %

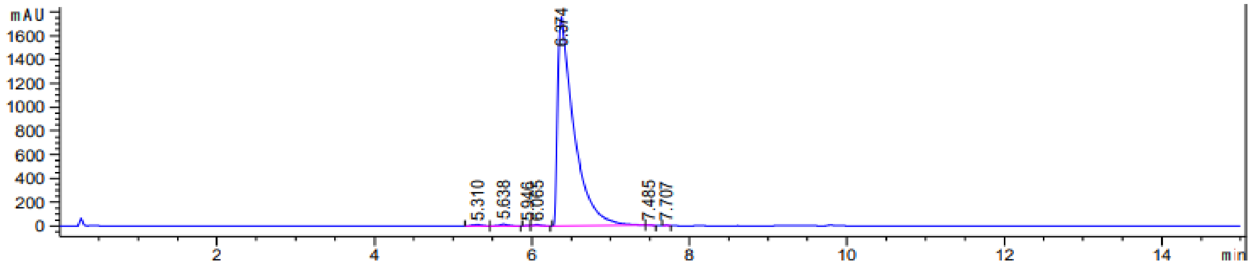

Signal 1: DAD1 A, Sig=254,4 Ref=360,100

Peak #	RetTime [min]	Type	Width [min]	Area [mAU*s]	Height [mAU]	Area %
1	5.310	BB	0.1303	83.51060	10.56129	0.3237
2	5.638	BB	0.0877	90.86881	14.37863	0.3523
3	5.946	BB	0.0501	6.76794	2.18639	0.0262
4	6.065	BB	0.0836	53.19125	10.06918	0.2062
5	6.374	BB	0.1978	2.55393e4	1754.48547	99.0088
6	7.485	BB	0.0432	9.12734	3.41516	0.0354
7	7.707	BB	0.0470	12.21989	4.32581	0.0474

Supplementary Figure 11. HPLC data of 1-(4-bromophenyl)-2-(4-(8-chloro-5,6-dihydro-11H-benzo[5,6]cyclohepta[1,2-b]pyridin-11-ylidene)piperidin-1-yl)ethan-1-one (8a)

HPLC purity 96.0869 %

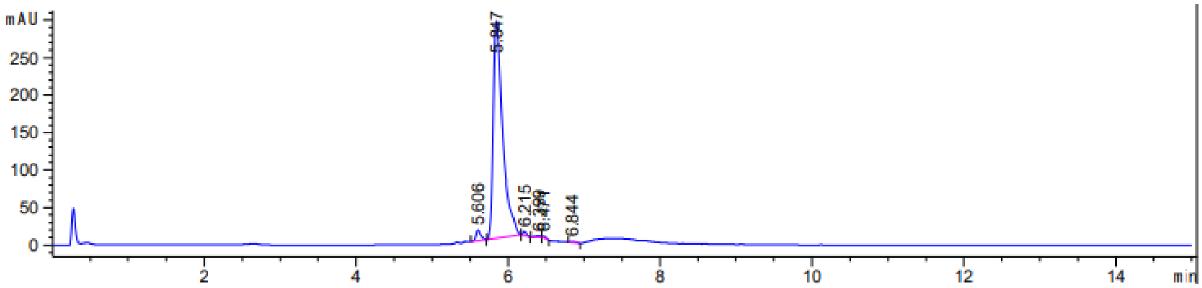

Signal 1: DAD1 A, Sig=254,4 Ref=360,100

Peak #	RetTime [min]	Type	Width [min]	Area [mAU*s]	Height [mAU]	Area %
1	5.606	BB	0.0604	59.17168	14.24999	2.3192
2	5.847	BB	0.1229	2451.52026	289.06812	96.0869
3	6.215	BB	0.0604	20.14680	5.54140	0.7897
4	6.399	BB	0.0605	6.83739	1.64398	0.2680
5	6.471	BB	0.0522	6.72521	2.05643	0.2636
6	6.844	BB	0.0861	6.95705	1.12571	0.2727

Supplementary Figure 12. HPLC data of 5-(2-(4-(8-chloro-5,6-dihydro-11H-benzo[5,6]-cyclohepta[1,2-b]pyridin-11-ylidene)piperidin-1-yl)acetyl)indolin-2-one (8b)

HPLC purity 95.8997 %

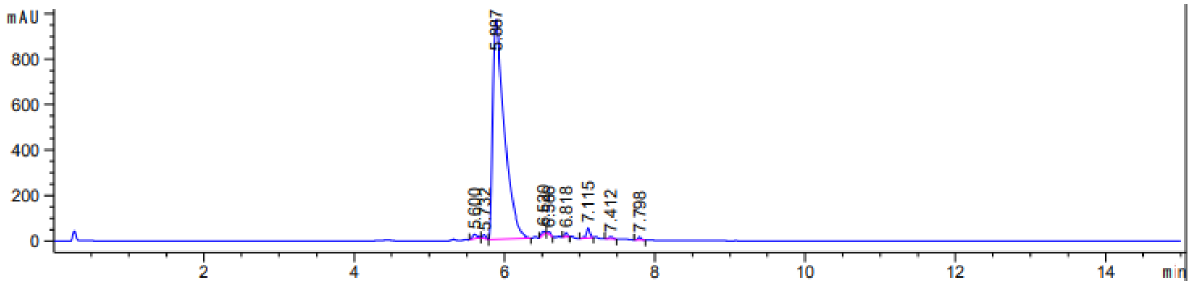

Signal 1: DAD1 A, Sig=254,4 Ref=360,100

Peak #	RetTime [min]	Type	Width [min]	Area [mAU*s]	Height [mAU]	Area %
1	5.600	BB	0.0631	76.39490	18.15882	0.7035
2	5.732	BB	0.0447	41.22150	14.71523	0.3796
3	5.887	BB	0.1519	1.04141e4	964.94427	95.8997
4	6.520	BB	0.0540	46.15461	14.18448	0.4250
5	6.586	BB	0.0480	25.99463	8.90760	0.2394
6	6.818	BB	0.0434	46.71498	17.33427	0.4302
7	7.115	BB	0.0505	140.33443	42.47170	1.2923
8	7.412	BB	0.0525	30.26831	9.17395	0.2787
9	7.798	BB	0.0476	38.18511	12.50169	0.3516

Supplementary Figure 13. HPLC data of 6-chloro-5-(2-(4-(8-chloro-5,6-dihydro-11H-benzo[5,6]cyclohepta[1,2-b]pyridin-11-ylidene)piperidin-1-yl)acetyl)indolin-2-one (8c)

HPLC purity 97.8945 %

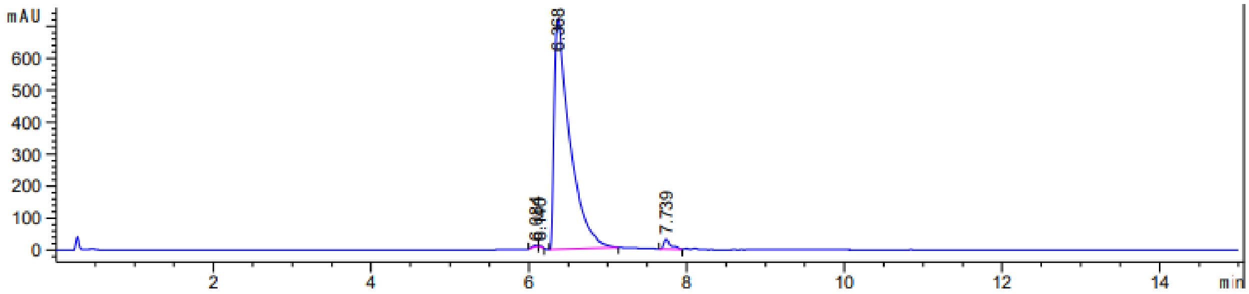

Signal 1: DAD1 A, Sig=254,4 Ref=360,100

Peak #	RetTime [min]	Type	Width [min]	Area [mAU*s]	Height [mAU]	Area %
1	6.084	BB	0.0732	19.13545	4.19402	0.1866
2	6.140	BB	0.0449	9.71819	3.44515	0.0948
3	6.368	BB	0.1902	1.00392e4	722.18024	97.8945
4	7.739	BB	0.0861	187.07043	31.12891	1.8242

Supplementary Figure 14. HPLC data of 1-(4-bromophenyl)-2-(4-(8-chloro-5,6-dihydro-11H-benzo[5,6]cyclohepta[1,2-b]pyridin-11-ylidene)piperidin-1-yl)ethan-1-ol (9a)

HPLC purity 99.0153 %

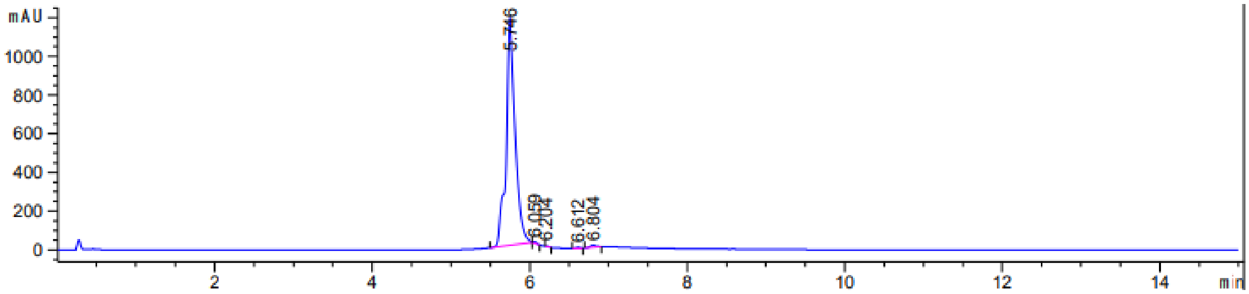

Signal 1: DAD1 A, Sig=254,4 Ref=360,100

Peak #	RetTime [min]	Type	Width [min]	Area [mAU*s]	Height [mAU]	Area %
1	5.746	BB	0.1134	9365.58105	1170.37390	99.0153
2	6.059	BB	0.0564	19.79689	5.72059	0.2093
3	6.204	BB	0.0685	5.37128	1.14996	0.0568
4	6.612	BB	0.0534	13.83679	4.09635	0.1463
5	6.804	BB	0.0677	54.13317	11.76958	0.5723

Supplementary Figure 15. HPLC data of 5-(2-(4-(8-chloro-5,6-dihydro-11H-benzo[5,6]-cyclohepta[1,2-b]pyridin-11-ylidene)piperidin-1-yl)-1-hydroxyethyl)indolin-2-one (9b)

HPLC purity 92.1700 %

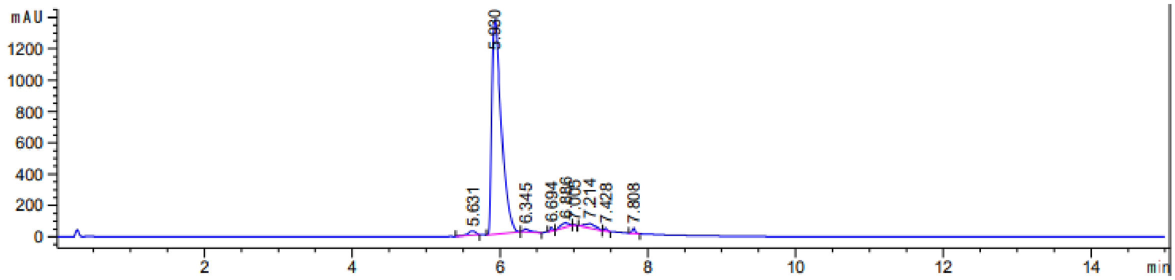

Signal 1: DAD1 A, Sig=254,4 Ref=360,100

Peak #	RetTime [min]	Type	Width [min]	Area [mAU*s]	Height [mAU]	Area %
1	5.631	BB	0.1121	198.45764	26.90147	1.4968
2	5.930	BB	0.1300	1.22205e4	1370.90503	92.1700
3	6.345	BB	0.0927	127.43721	19.37711	0.9612
4	6.694	BB	0.0463	65.04462	23.50346	0.4906
5	6.886	BB	0.1250	217.54041	27.89153	1.6407
6	7.005	BB	0.0379	11.35183	5.11755	0.0856
7	7.214	BB	0.1389	275.90240	27.54308	2.0809
8	7.428	BB	0.0435	45.07892	16.69436	0.3400
9	7.808	BB	0.0466	97.34361	32.80747	0.7342

Supplementary Figure 16. HPLC data of 6-chloro-5-(2-(4-(8-chloro-5,6-dihydro-11H-benzo[5,6]cyclohepta[1,2-b]pyridin-11-ylidene)piperidin-1-yl)-1-hydroxyethyl)indolin-2-one (9c)

HPLC purity 99.7003 %

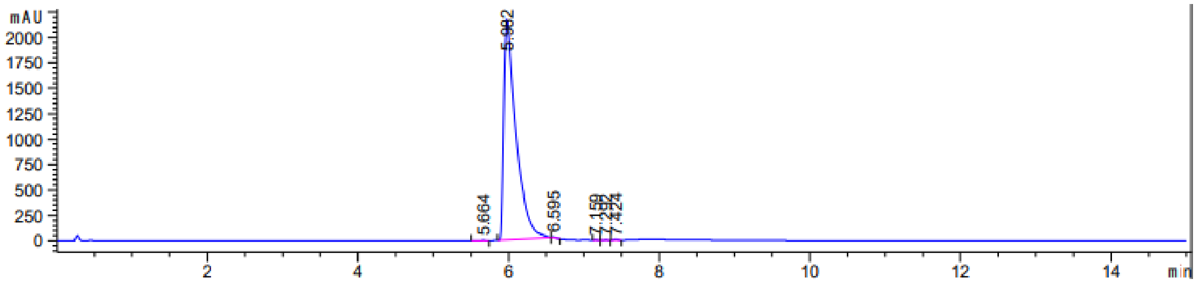

Signal 1: DAD1 A, Sig=254,4 Ref=360,100

Peak #	RetTime [min]	Type	Width [min]	Area [mAU*s]	Height [mAU]	Area %
1	5.664	BB	0.0789	8.38831	1.55594	0.0341
2	5.982	BB	0.1600	2.45193e4	2167.85132	99.7003
3	6.595	BB	0.0494	16.79679	5.23482	0.0683
4	7.159	BB	0.0441	5.16720	1.87826	0.0210
5	7.292	BB	0.0514	8.76037	2.59311	0.0356
6	7.424	BB	0.0539	34.60248	10.12756	0.1407

Supplementary Figure 17. HPLC data of 6-chloro-5-(2-(4-(8-chloro-5,6-dihydro-11H-benzo[5,6]cyclohepta[1,2-b]pyridin-11-ylidene) piperidin-1-yl)ethyl)indolin-2-one (11a)

HPLC purity 100 %

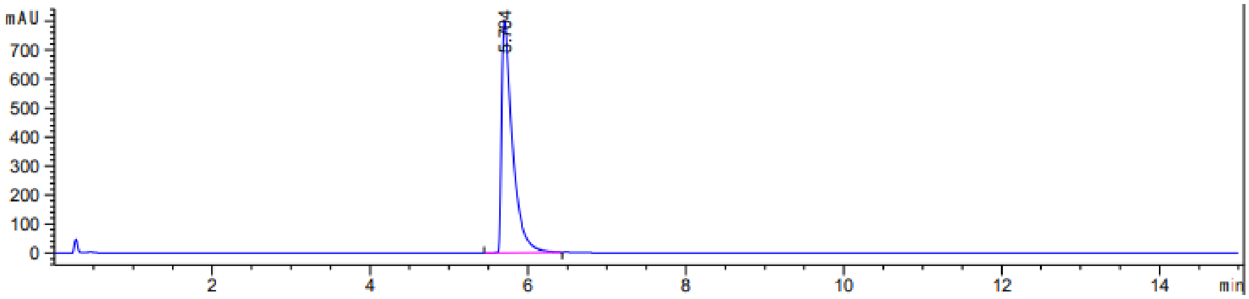

Signal 1: DAD1 A, Sig=254,4 Ref=360,100

Peak #	RetTime [min]	Type	Width [min]	Area [mAU*s]	Height [mAU]	Area %
1	5.704	BB	0.1387	7742.81396	801.69470	100.0000

Supplementary Figure 18. HPLC data of 3-(2-(4-(8-chloro-5,6-dihydro-11H-benzo[5,6]-cyclohepta[1,2-b]pyridin-11-ylidene)piperidin-1-yl)ethyl)-2-methyl-6,7,8,9-tetrahydro-4H-pyrido-[1,2-a]pyrimidin-4-one (11b)

HPLC purity 100 %

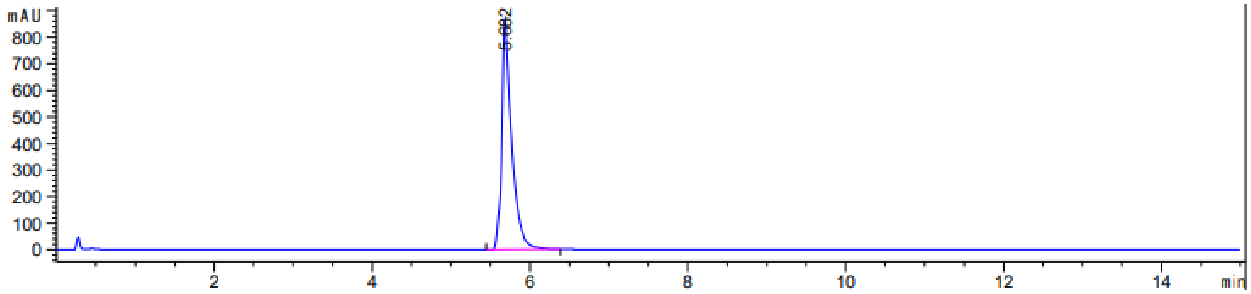

Signal 1: DAD1 A, Sig=254,4 Ref=360,100

Peak #	RetTime [min]	Type	Width [min]	Area [mAU*s]	Height [mAU]	Area %
1	5.682	BB	0.1231	7688.88525	870.16510	100.0000

Supplementary Figure 19. HPLC data of 3-(2-(4-(8-chloro-5,6-dihydro-11H-benzo[5,6]-cyclohepta[1,2-b]pyridin-11-ylidene)piperidin-1-yl)ethyl)-9-hydroxy-2-methyl-6,7,8,9-tetrahydro-4H-pyrido[1,2-a]pyrimidin-4-one (11c)

HPLC purity 99.6548 %

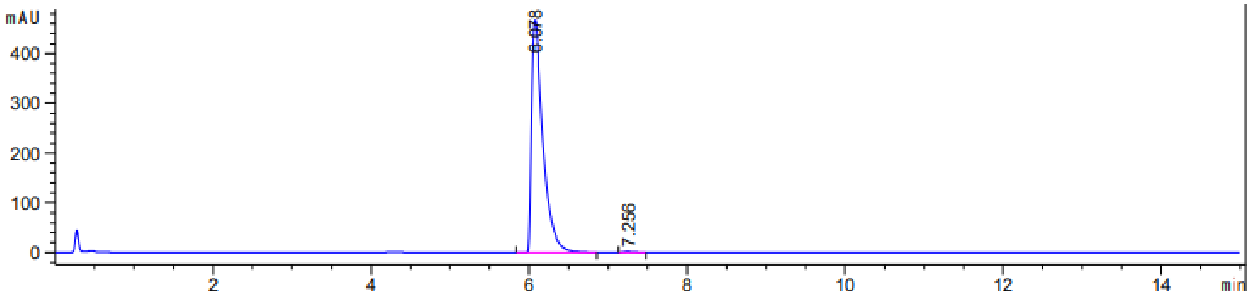

Signal 1: DAD1 A, Sig=254,4 Ref=360,100

Peak #	RetTime [min]	Type	Width [min]	Area [mAU*s]	Height [mAU]	Area %
1	6.078	BB	0.1439	4643.43213	467.33121	99.6548
2	7.256	BB	0.1358	16.08539	1.64898	0.3452

Supplementary Figure 20. HPLC data of 3-(2-(4-(8-chloro-5,6-dihydro-11H-benzo[5,6]-cyclohepta[1,2-b]pyridin-11-ylidene)piperidin-1-yl)ethyl)quinazoline-2,4(1H,3H)-dione (11d)

HPLC purity 95.7449 %

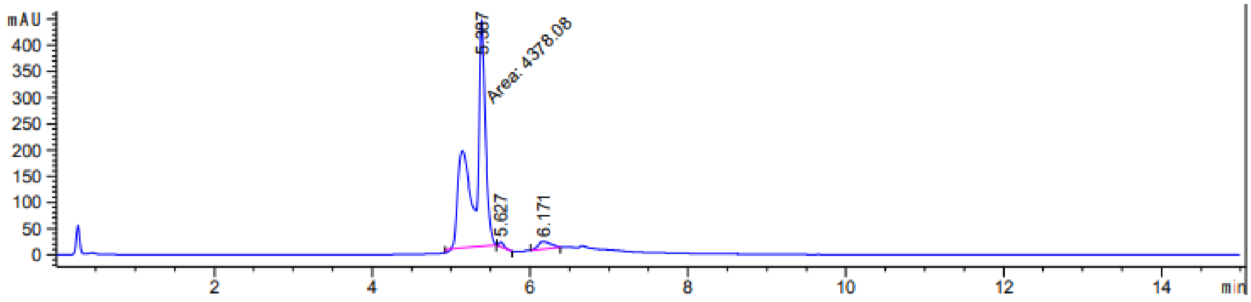

Signal 1: DAD1 A, Sig=254,4 Ref=360,100

Peak #	RetTime [min]	Type	Width [min]	Area [mAU*s]	Height [mAU]	Area %
1	5.387	MM T	0.1703	4378.08398	428.56100	95.7449
2	5.627	BB	0.0700	38.73507	9.03807	0.8471
3	6.171	BB	0.1727	155.83498	13.70349	3.4080

Supplementary Figure 21. HPLC data of 3-(2-(4-(5,6-dihydro-11H-benzo[5,6]cyclohepta[1,2-b]pyridin-11-ylidene)piperidin-1-yl)ethyl)-9-hydroxy-2-methyl-6,7,8,9-tetrahydro-4H-pyrido[1,2-a]pyrimidin-4-one (11e)

HPLC purity 98.8504 %

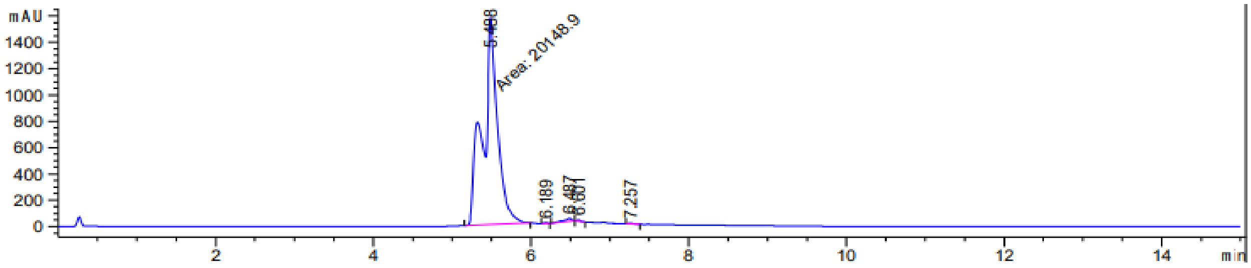

Signal 1: DAD1 A, Sig=254,4 Ref=360,100

Peak #	RetTime [min]	Type	Width [min]	Area [mAU*s]	Height [mAU]	Area %
1	5.488	MM T	0.2148	2.01489e4	1563.26624	98.8504
2	6.189	BB	0.0511	14.07063	4.43044	0.0690
3	6.487	BB	0.0974	166.19809	23.24258	0.8154
4	6.601	BB	0.0467	29.61365	9.95234	0.1453
5	7.257	BB	0.0688	24.44105	5.60811	0.1199

Supplementary Figure 22. HPLC data of 9-hydroxy-3-(2-(4-(8-methyl-5,6-dihydro-11H-benzo[5,6] cyclohepta[1,2-b]pyridin-11-ylidene)piperidin-1-yl)ethyl)-2-methyl-6,7,8,9-tetrahydro-4H-pyrido[1,2-a]pyrimidin-4-one (21)

HPLC purity 99.5089 %

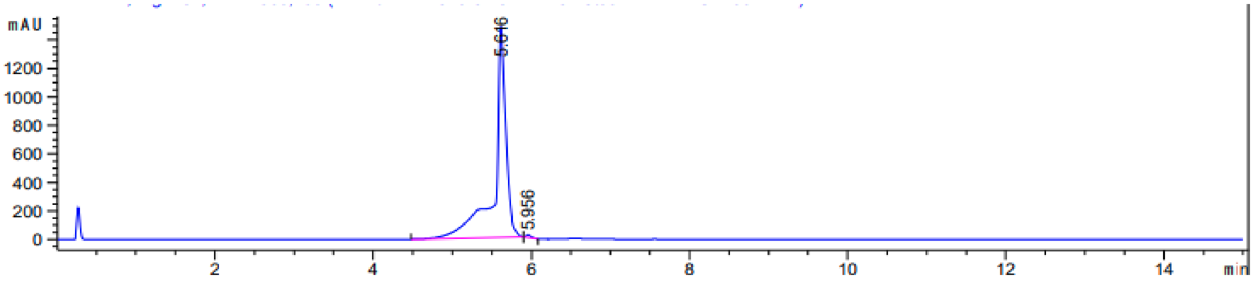

Signal 1: DAD1 A, Sig=254,4 Ref=360,100

Peak #	RetTime [min]	Type	Width [min]	Area [mAU*s]	Height [mAU]	Area %
1	5.616	BB	0.1311	1.46349e4	1482.81470	99.5089
2	5.956	BB	0.0616	72.22642	17.68855	0.4911

Supplementary Figure 23. HPLC data of (S)-3-(2-(4-(8-chloro-5,6-dihydro-11H-benzo[5,6]cyclohepta[1,2-b]pyridin-11-ylidene) piperidin-1-yl)ethyl)-9-hydroxy-2-methyl-6,7,8,9-tetrahydro-4H-pyrido[1,2-a]pyrimidin-4-one (22f)

HPLC purity 99.9353 %

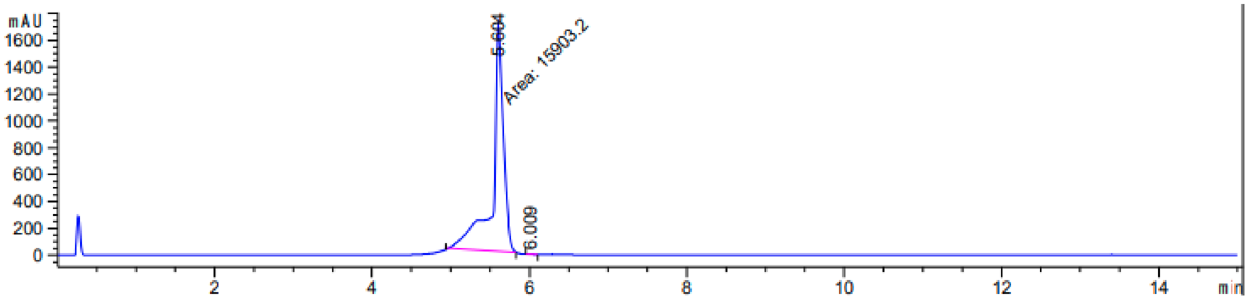

Signal 1: DAD1 A, Sig=254,4 Ref=360,100

Peak #	RetTime [min]	Type	Width [min]	Area [mAU*s]	Height [mAU]	Area %
1	5.604	MM T	0.1565	1.59032e4	1693.79016	99.9353
2	6.009	BB	0.0671	10.30315	2.44985	0.0647

Supplementary Figure 24. HPLC data of (R)-3-(2-(4-(8-chloro-5,6-dihydro-11H-benzo[5,6]cyclohepta[1,2-b]pyridin-11-ylidene) piperidin-1-yl)ethyl)-9-hydroxy-2-methyl-6,7,8,9-tetrahydro-4H-pyrido[1,2-a]pyrimidin-4-one (22g)

<Chromatogram>

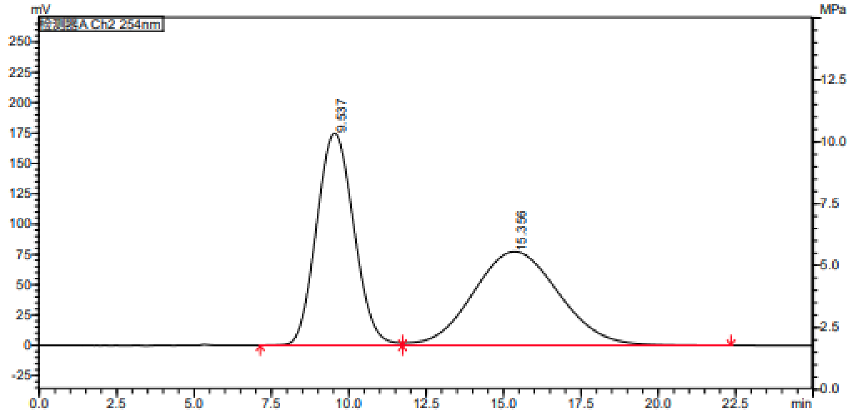

<Peak Table>

Peak#	Ret. Time	Area	Area%	T.Plate#	Tailing F.	Resolution
1	9.537	14715710	49.695	292	1.100	--
2	15.356	14896384	50.305	145	1.072	1.587

Supplementary Figure 25. Chiral HPLC purity of racemic 11c

<Chromatogram>

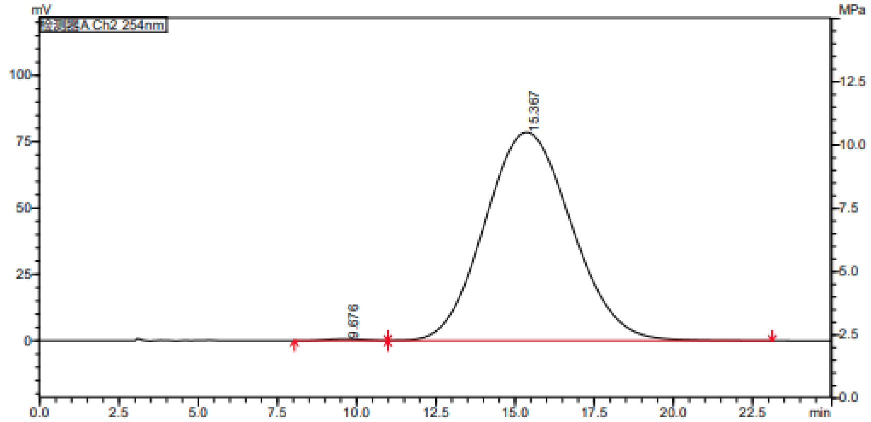

<Peak Table>

Peak#	Ret. Time	Area	Area%	T.Plate#	Tailing F.	Resolution
1	9.676	53959	0.358	346	--	--
2	15.367	15007573	99.642	146	1.086	1.587

Supplementary Figure 26. Chiral HPLC purity of (+)-22f (S isomer)

<Chromatogram>

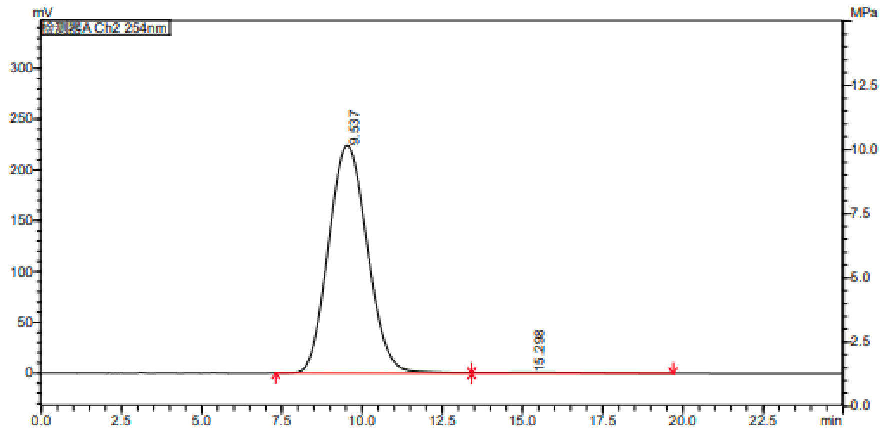

<Peak Table>

Peak#	Ret. Time	Area	Area%	T.Plate#	Tailing F.	Resolution
1	9.537	18904477	99.169	292	1.099	--
2	15.298	158487	0.831	83	--	1.290

Supplementary Figure 27. Chiral HPLC purity of (-)-22g (R isomer)

^{1}H NMR

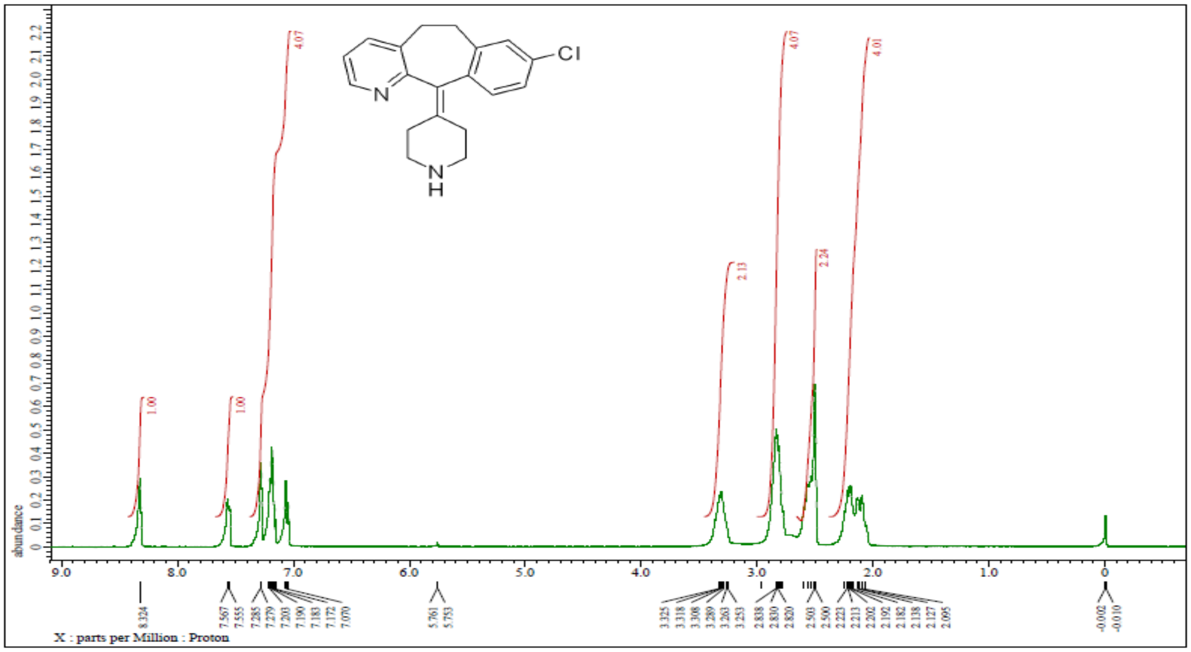

^{13}C NMR

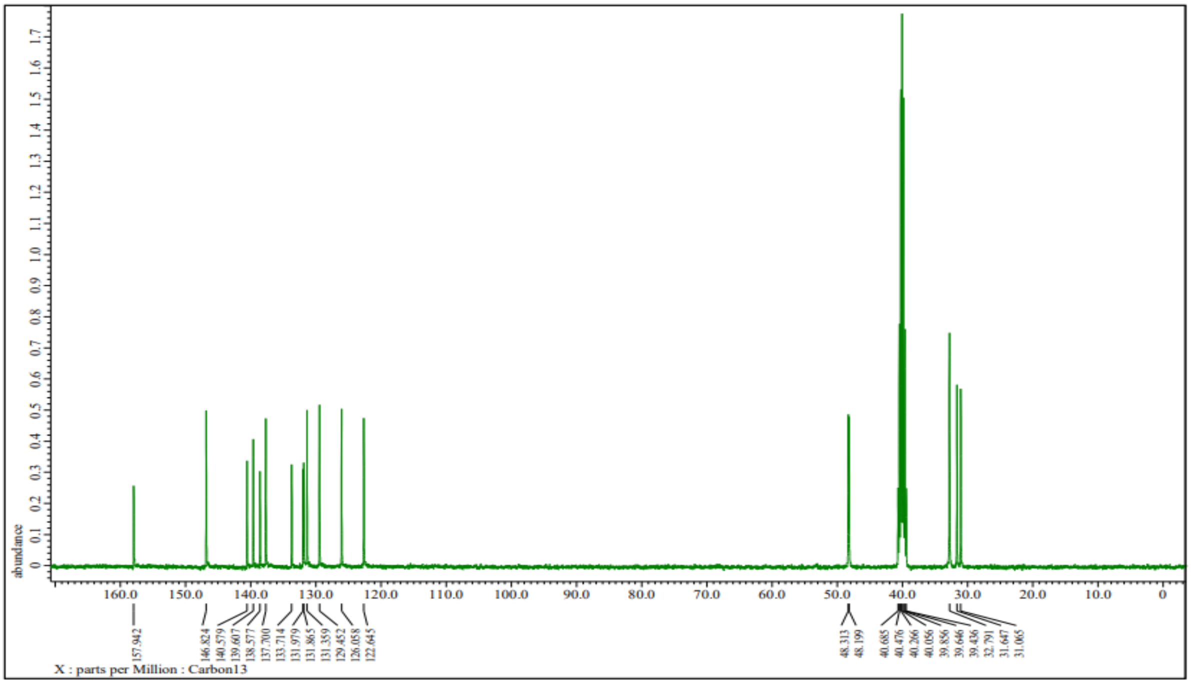

Supplementary Figure 28. NMR data of 8-chloro-11-(piperidin-4-ylidene)-6,11-dihydro-5H-benzo[5,6]cyclohepta[1,2-b]pyridine (2)

^{1}H NMR

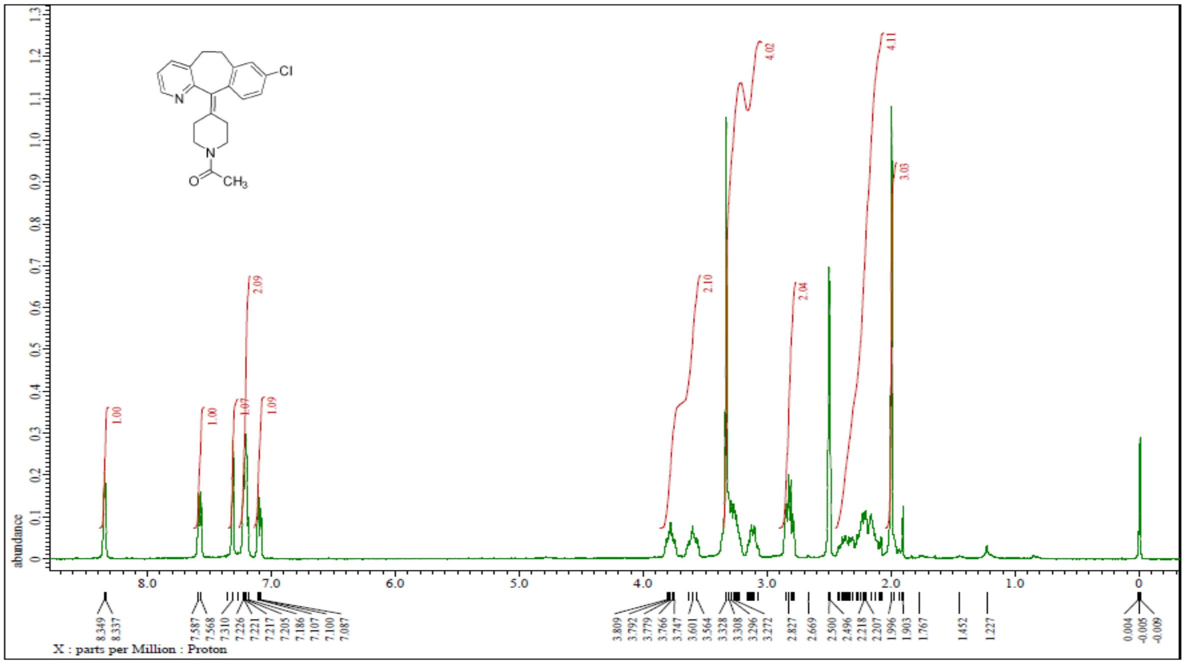

^{13}C NMR

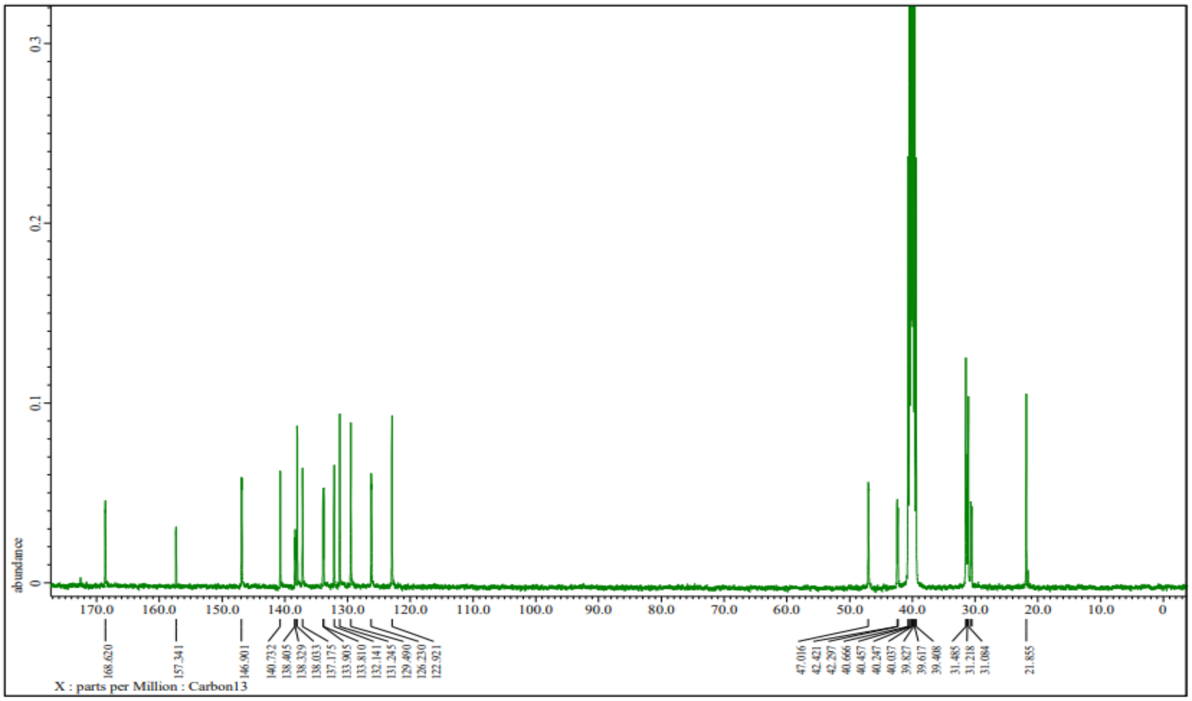

Supplementary Figure 29. NMR data of 1-(4-(8-chloro-5,6-dihydro-11H-benzo[5,6]-cyclohepta[1,2-b]pyridin-11-ylidene)piperidin-1-yl)ethan-1-one (3)

^{1}H NMR

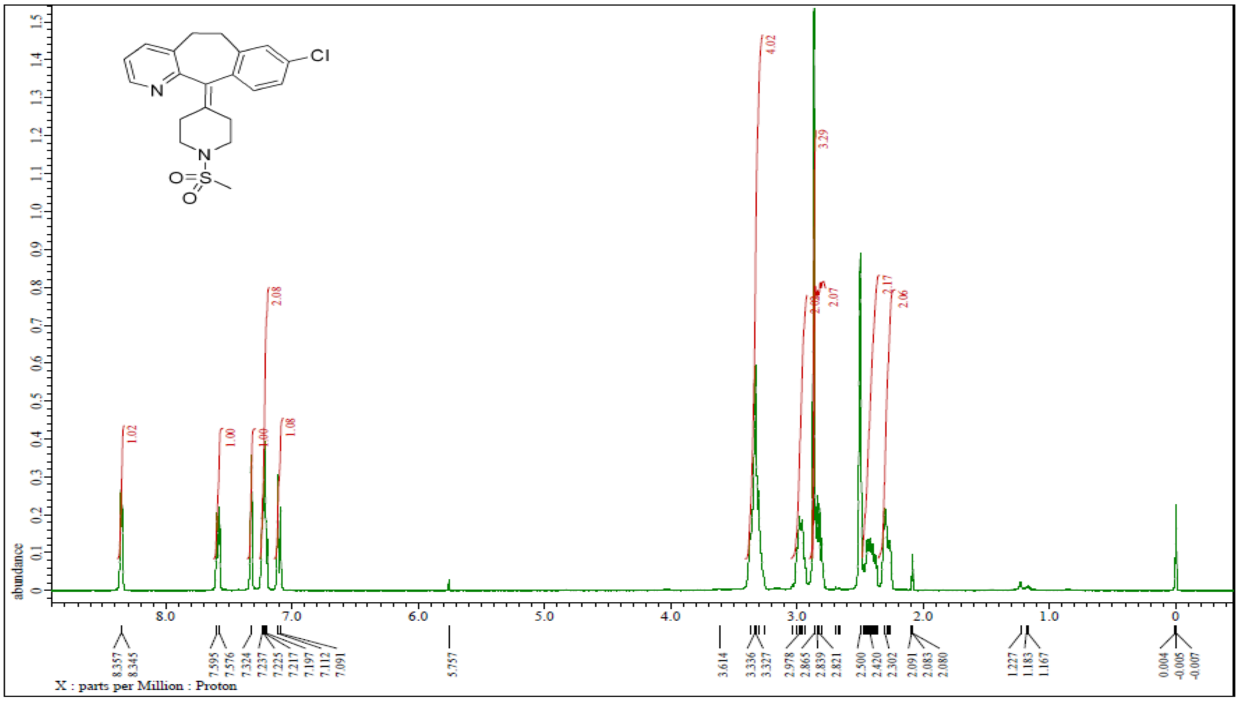

^{13}C NMR

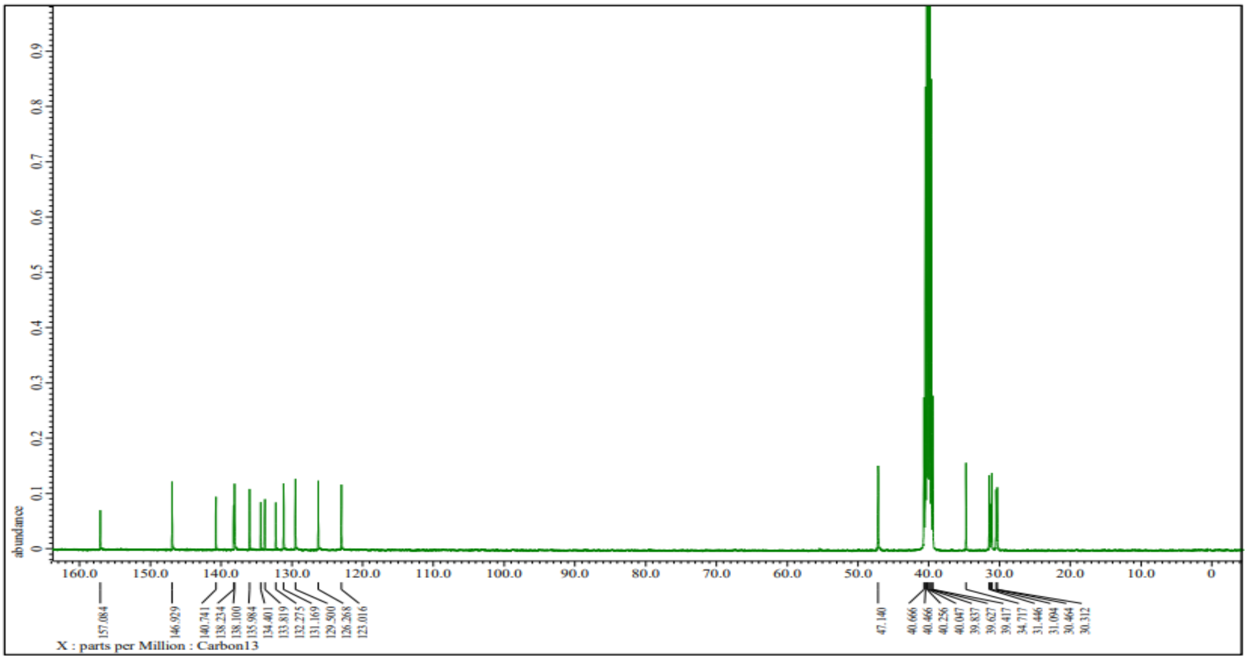

Supplementary Figure 30. NMR data of 8-chloro-11-(1-(methylsulfonyl)piperidin-4-ylidene)-6,11-dihydro-5H-benzo[5,6] cyclohepta[1,2-b]pyridine (4)

^{1}H NMR

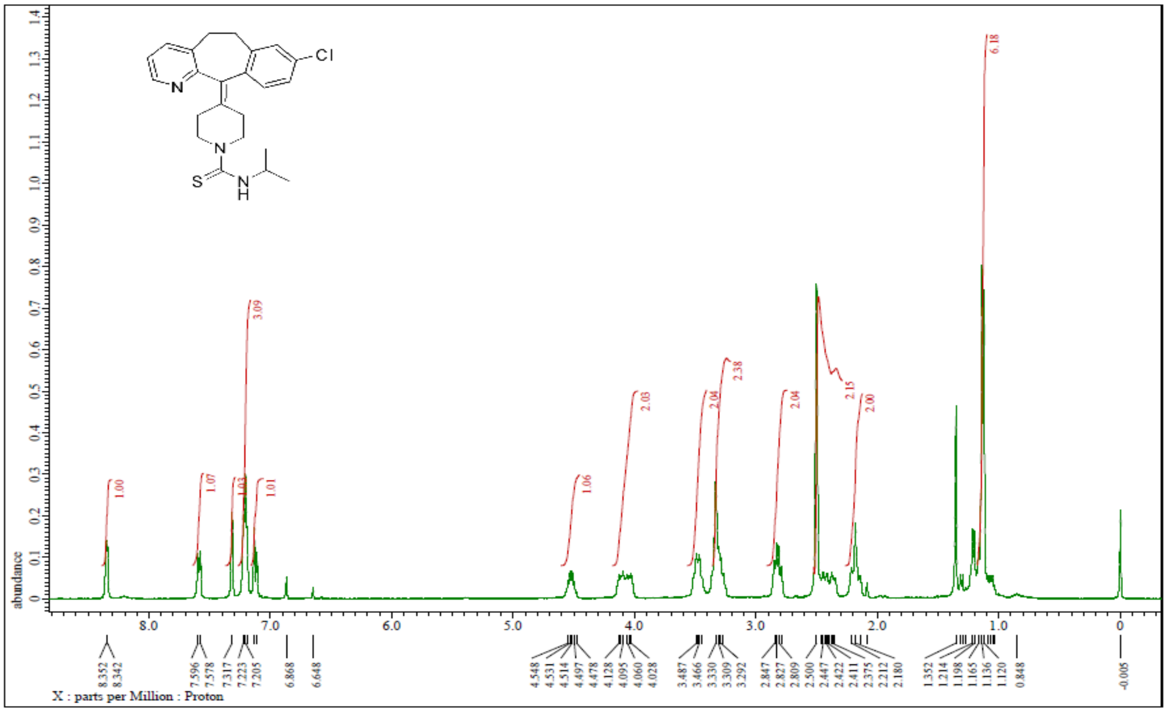

^{13}C NMR

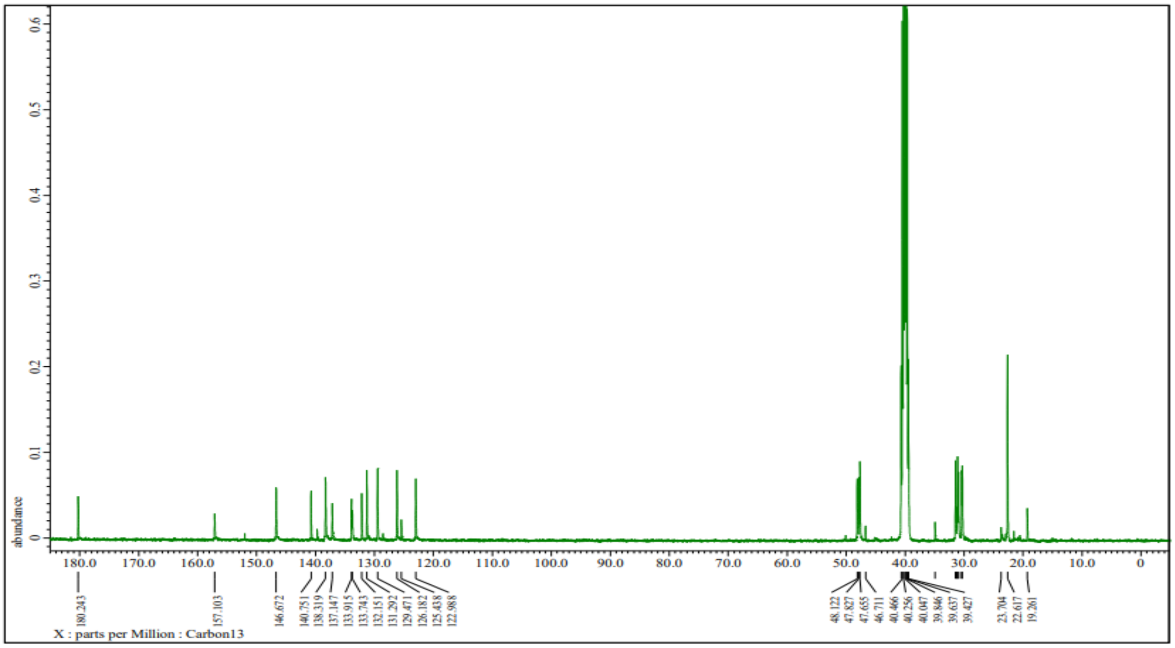

Supplementary Figure 31. NMR data of 4-(8-chloro-5,6-dihydro-11H-benzo[5,6]cyclohepta[1,2-b]pyridin-11-ylidene)-N-isopropylpiperidine-1-carbothioamide (5)

^{1}H NMR

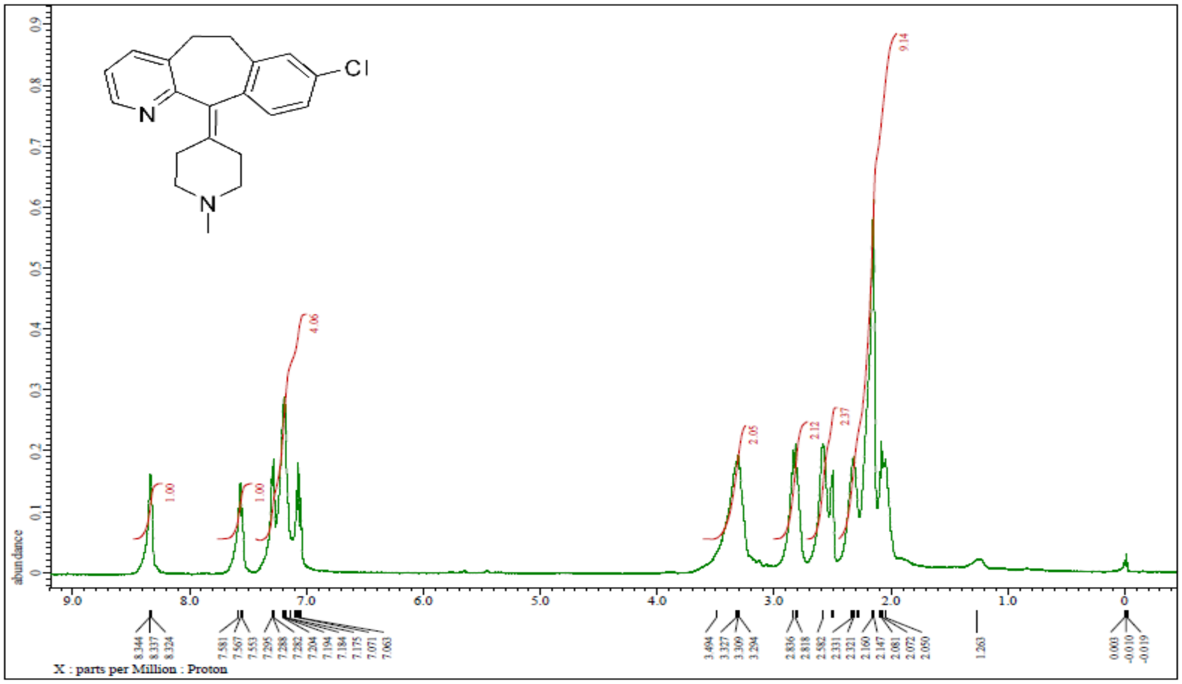

^{13}C NMR

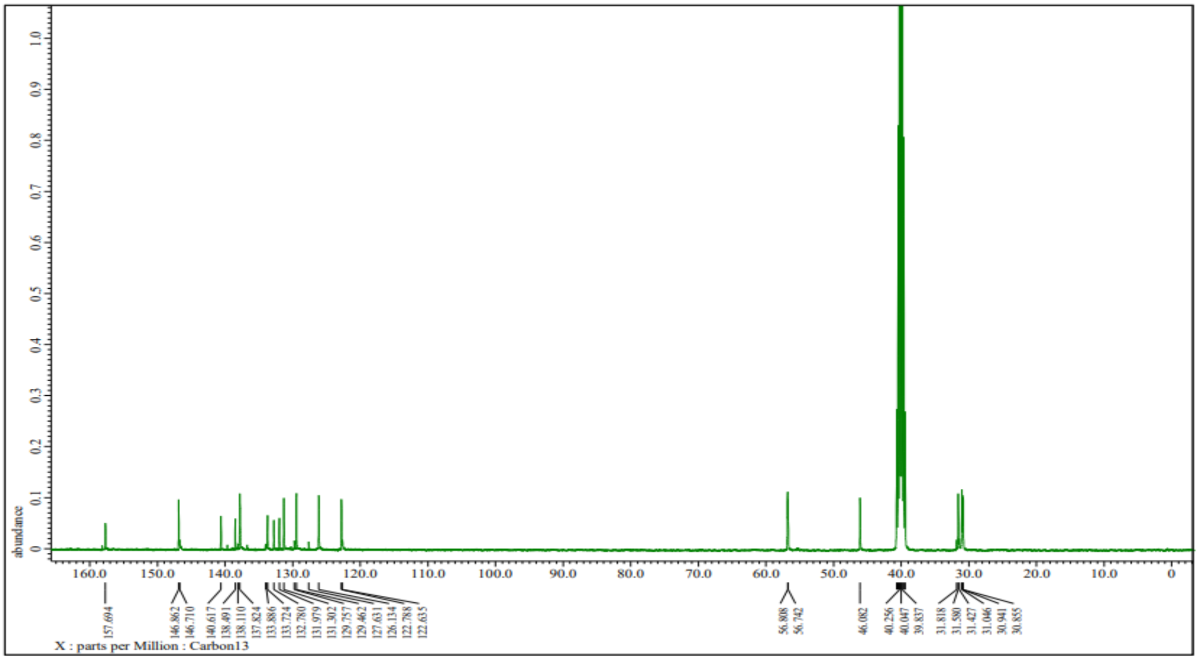

Supplementary Figure 32. NMR data of 8-chloro-11-(1-methylpiperidin-4-ylidene)-6,11-dihydro-5H-benzo[5,6]cyclohepta[1,2-b]pyridine (6)

^{1}H NMR

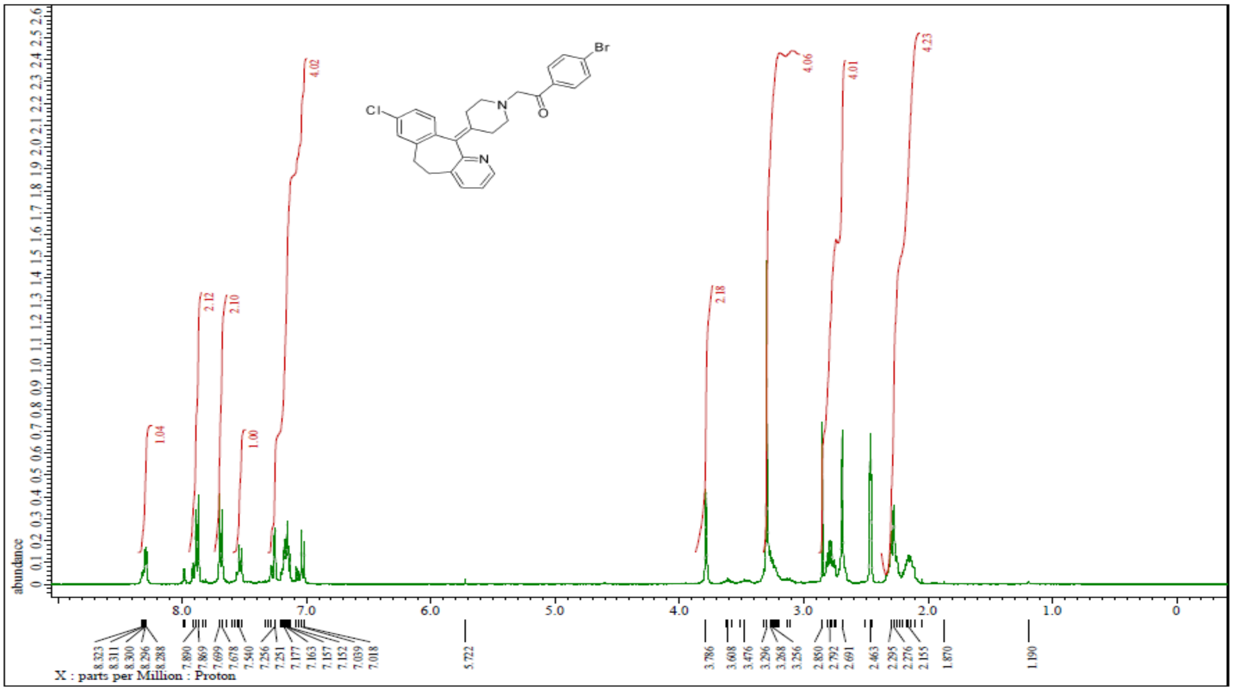

^{13}C NMR

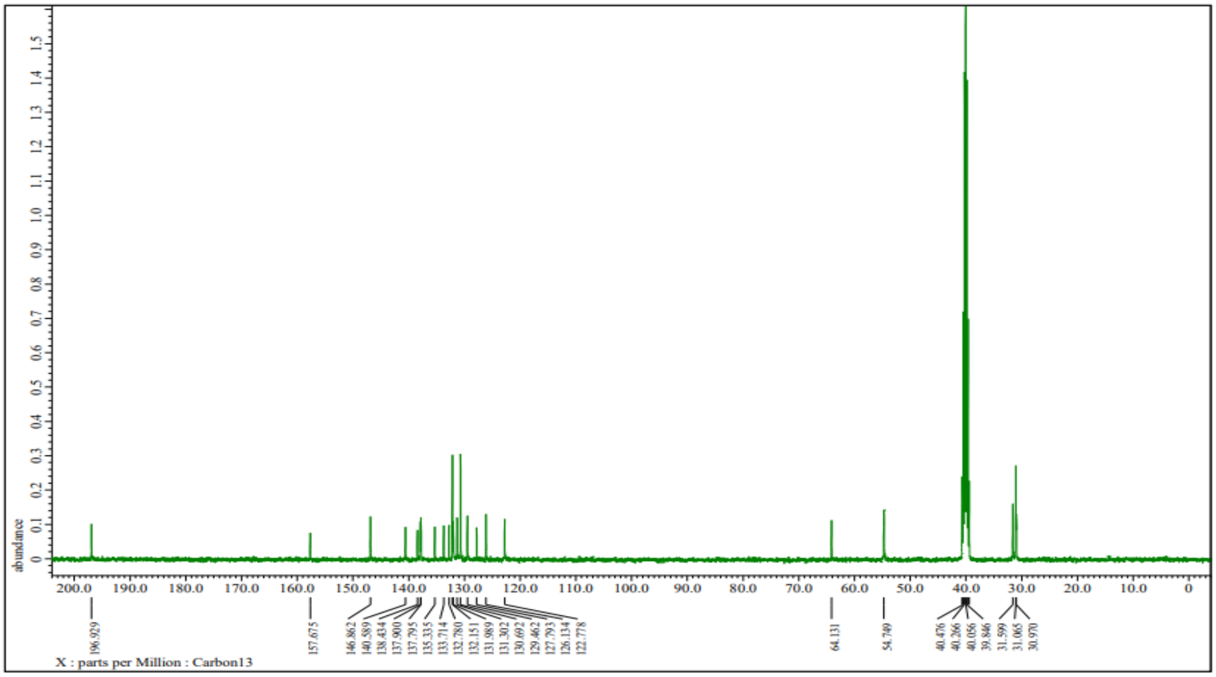

Supplementary Figure 33. NMR data of 1-(4-bromophenyl)-2-(4-(8-chloro-5,6-dihydro-11H-benzo[5,6]cyclohepta[1,2-b]pyridin-11-ylidene)piperidin-1-yl)ethan-1-one (8a)

^{1}H NMR

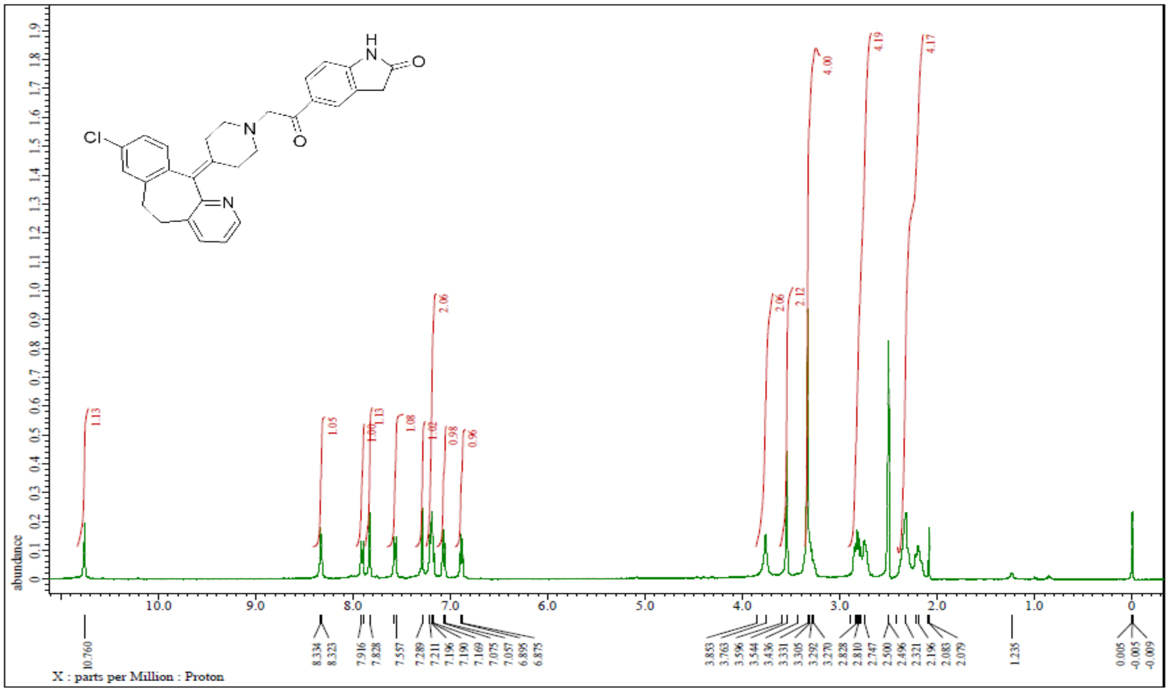

^{13}C NMR

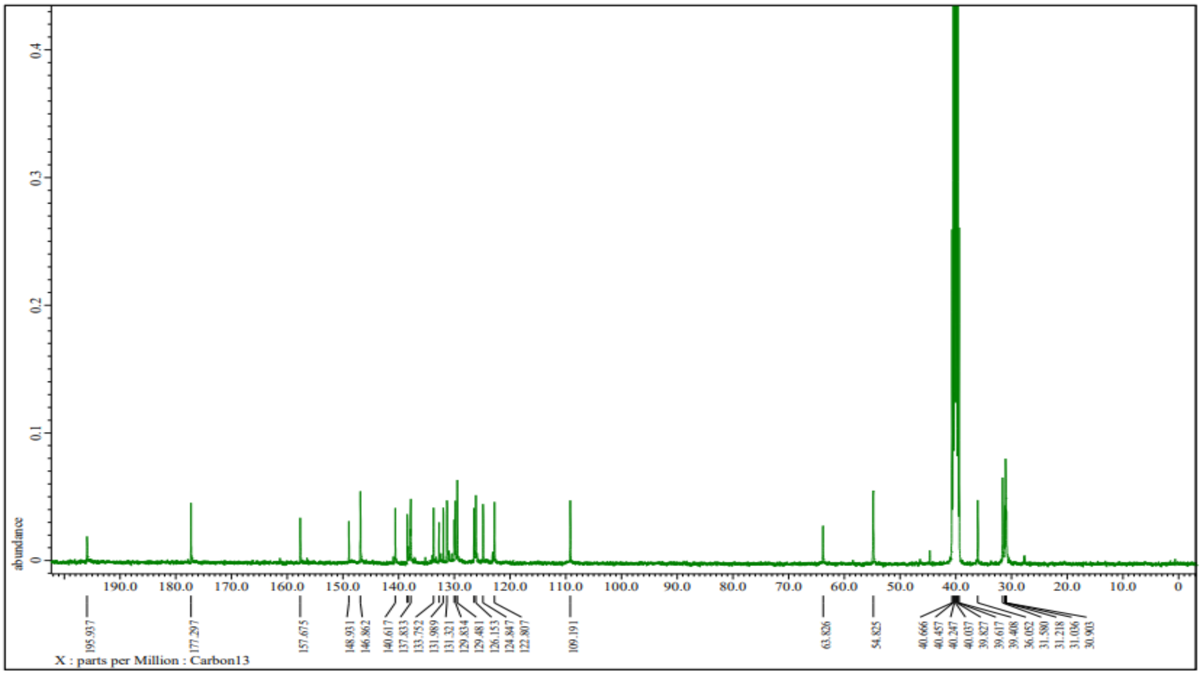

Supplementary Figure 34. NMR data of 5-(2-(4-(8-chloro-5,6-dihydro-11H-benzo[5,6]-cyclohepta[1,2-b]pyridin-11-ylidene)piperidin-1-yl)acetyl)indolin-2-one (8b)

^{1}H NMR

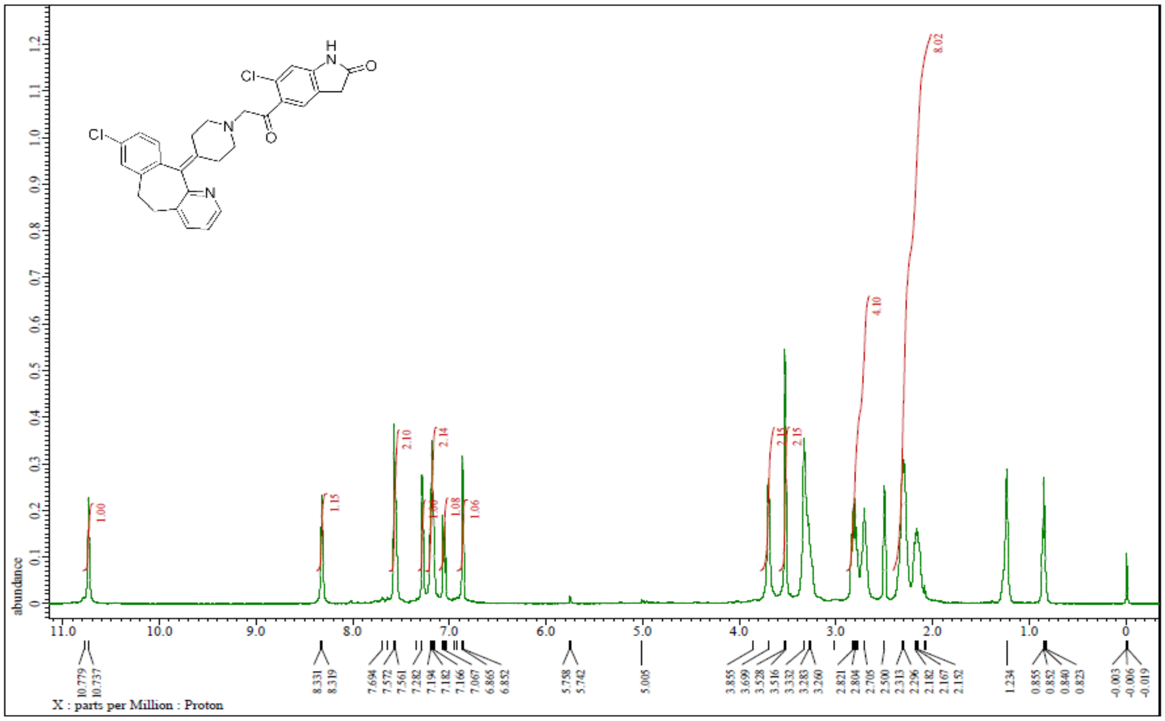

^{13}C NMR

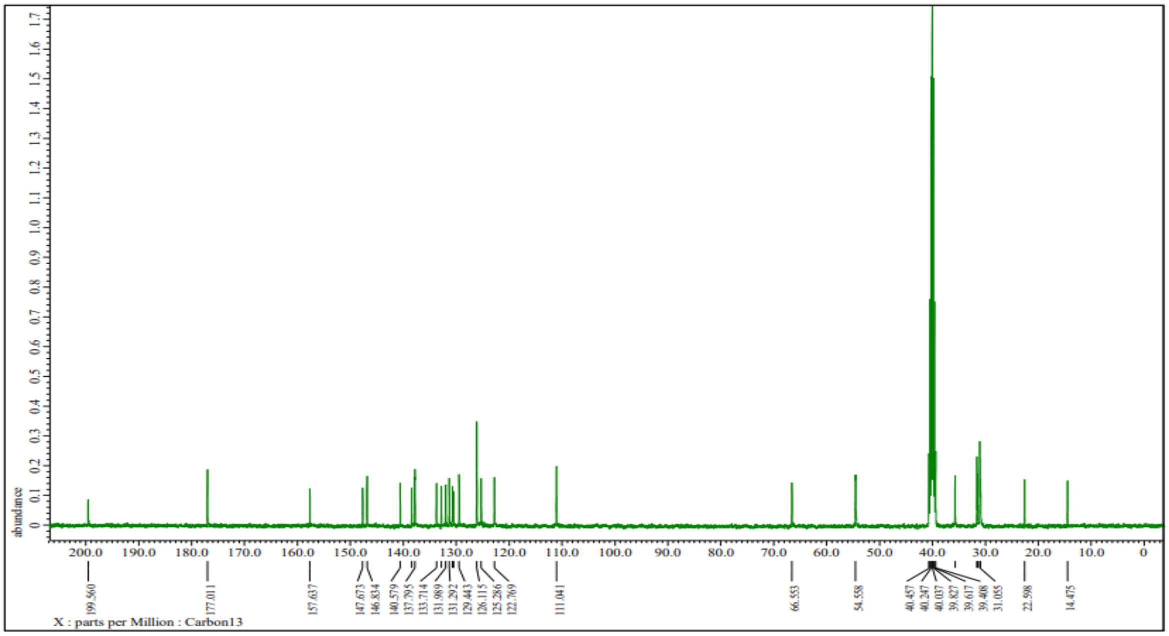

Supplementary Figure 35. NMR data of 6-chloro-5-(2-(4-(8-chloro-5,6-dihydro-11H-benzo[5,6]cyclohepta[1,2-b]pyridin-11-ylidene)piperidin-1-yl)acetyl)indolin-2-one (8c)

^{1}H NMR

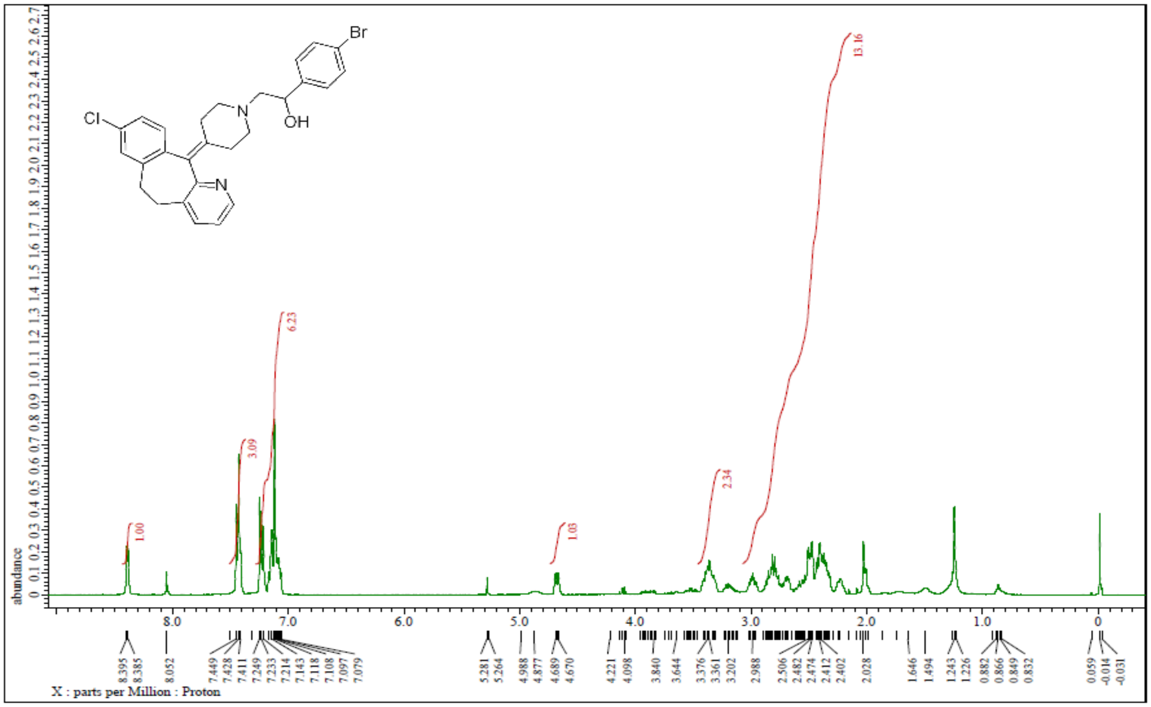

^{13}C NMR

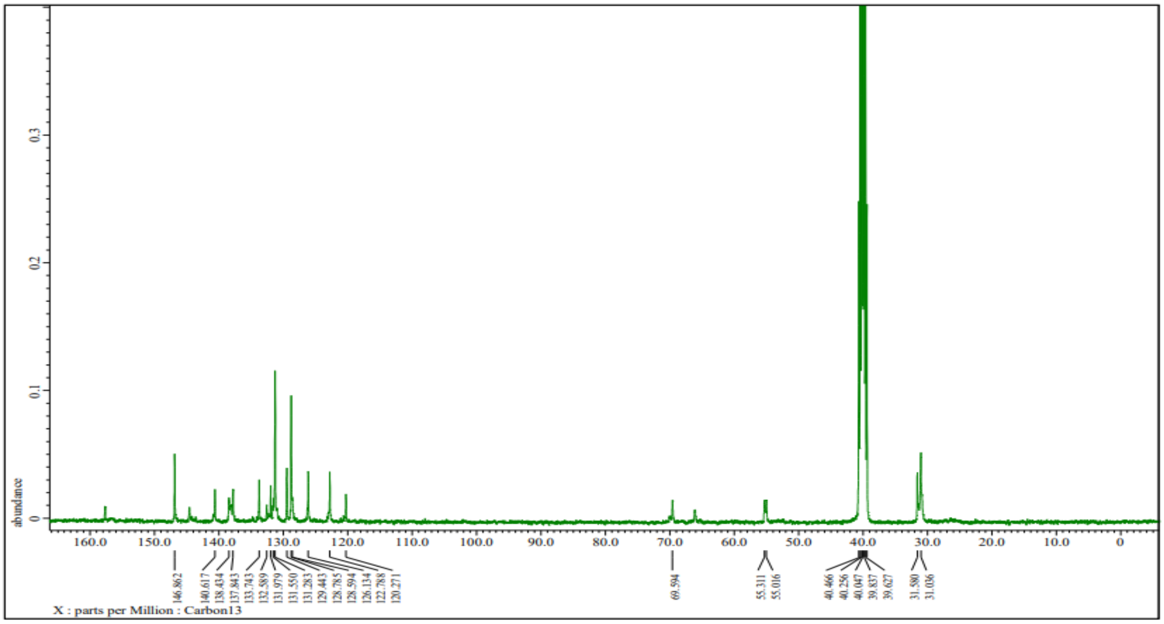

Supplementary Figure 36. NMR data of 1-(4-bromophenyl)-2-(4-(8-chloro-5,6-dihydro-11H-benzo[5,6]cyclohepta[1,2-b]pyridin-11-ylidene)piperidin-1-yl)ethan-1-ol (9a)

^{1}H NMR

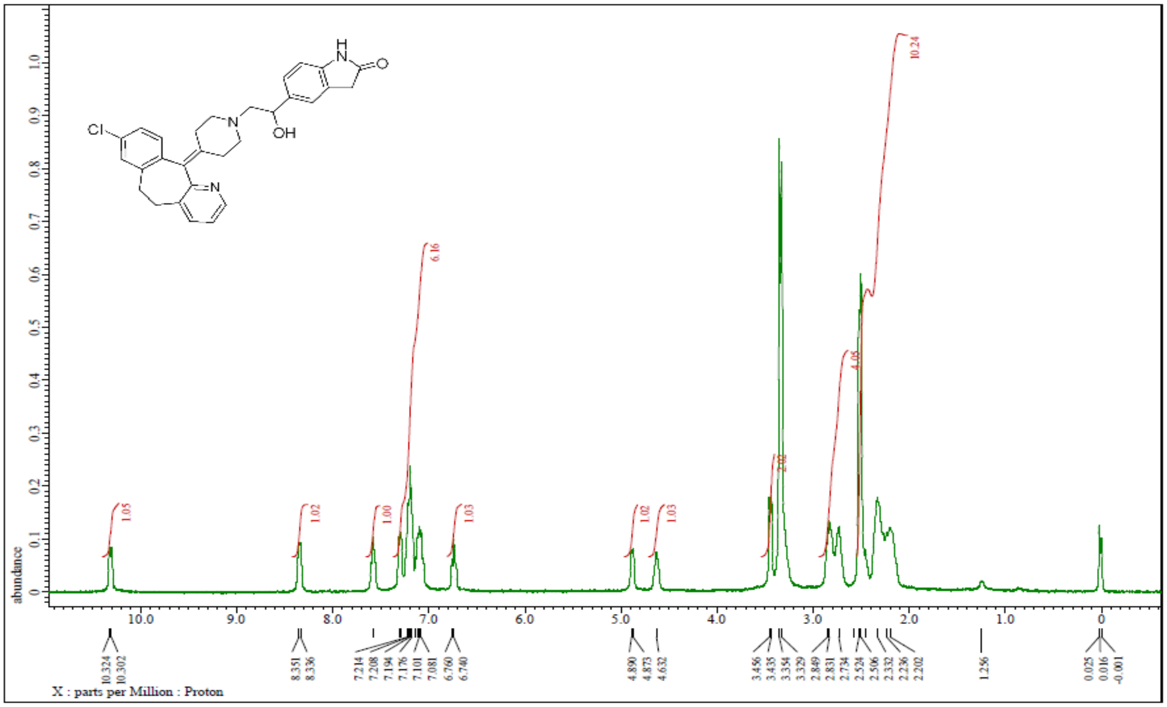

^{13}C NMR

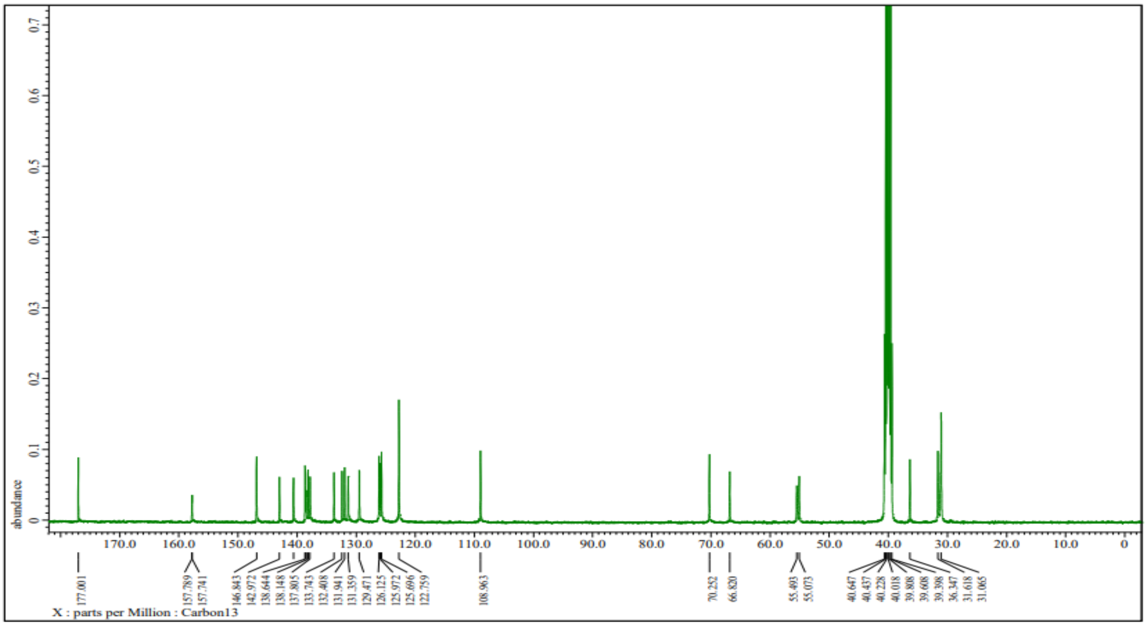

Supplementary Figure 37. NMR data of 5-(2-(4-(8-chloro-5,6-dihydro-11H-benzo[5,6]-cyclohepta[1,2-b]pyridin-11-ylidene)piperidin-1-yl)-1-hydroxyethyl)indolin-2-one(9b)

^{1}H NMR

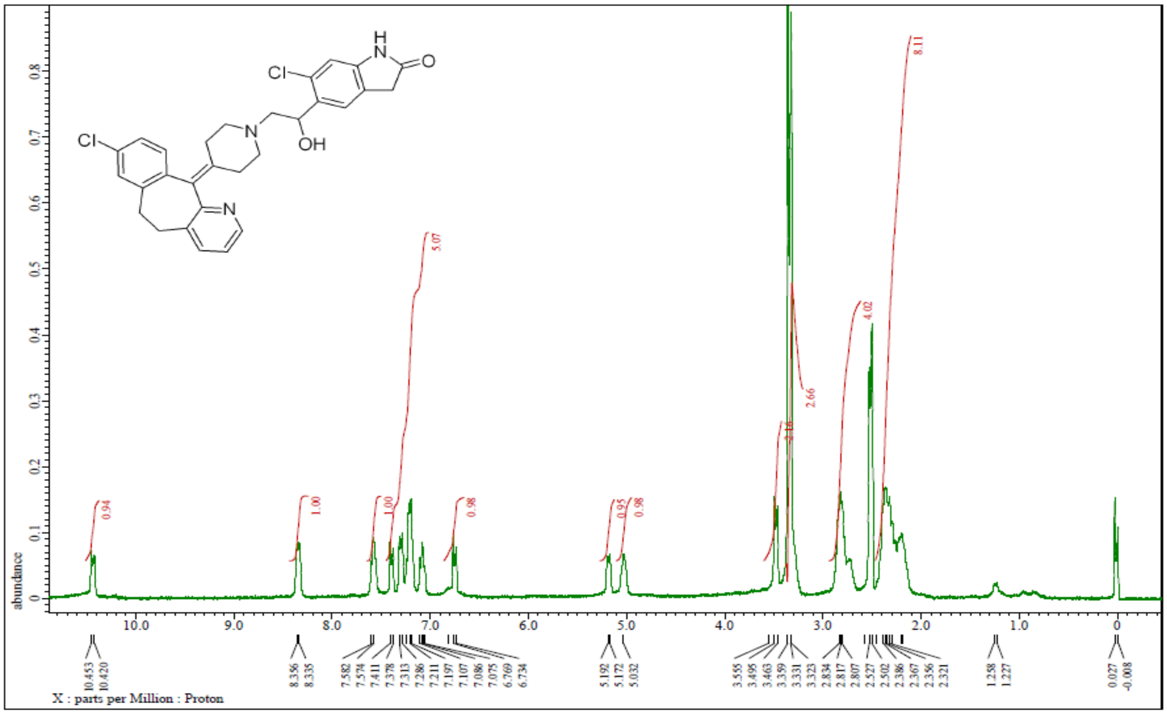

^{13}C NMR

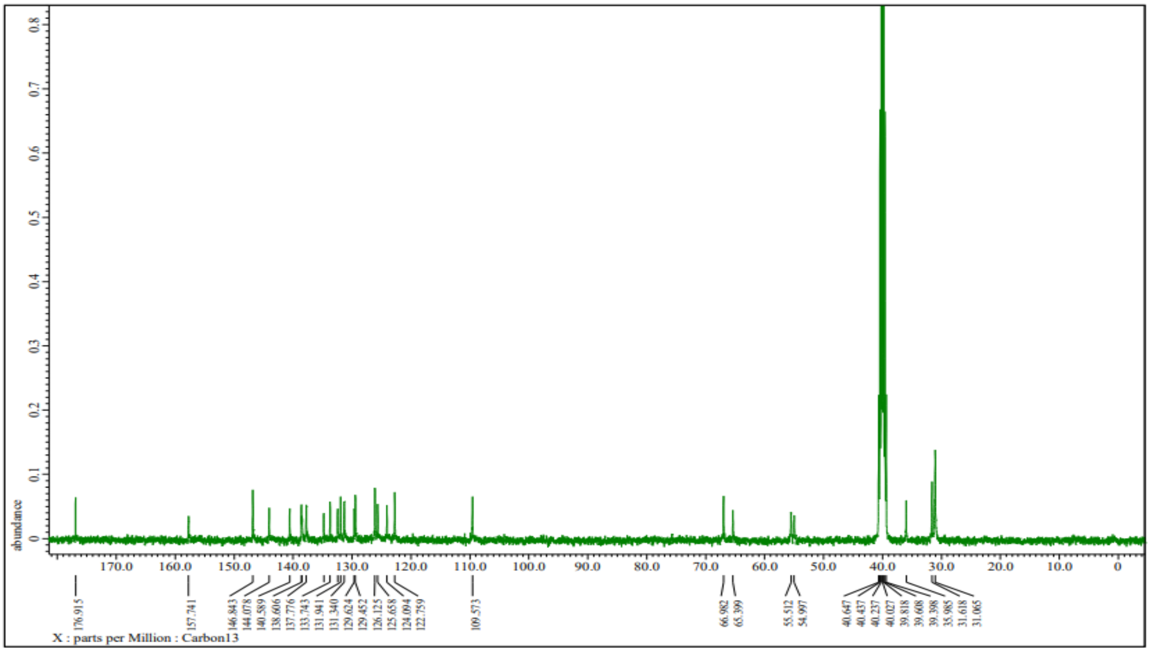

Supplementary Figure 38. NMR data of 6-chloro-5-(2-(4-(8-chloro-5,6-dihydro-11H-benzo[5,6]cyclohepta[1,2-b]pyridin-11-ylidene)piperidin-1-yl)-1-hydroxyethyl)indolin-2-one (9c)

^{1}H NMR

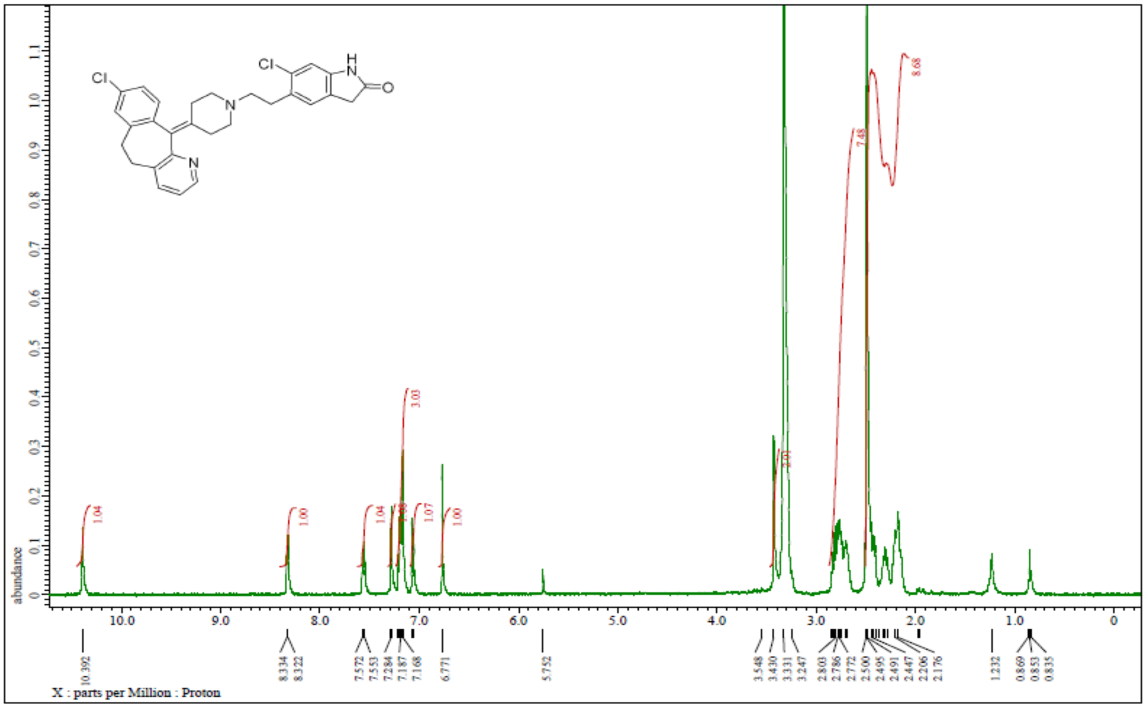

^{13}C NMR

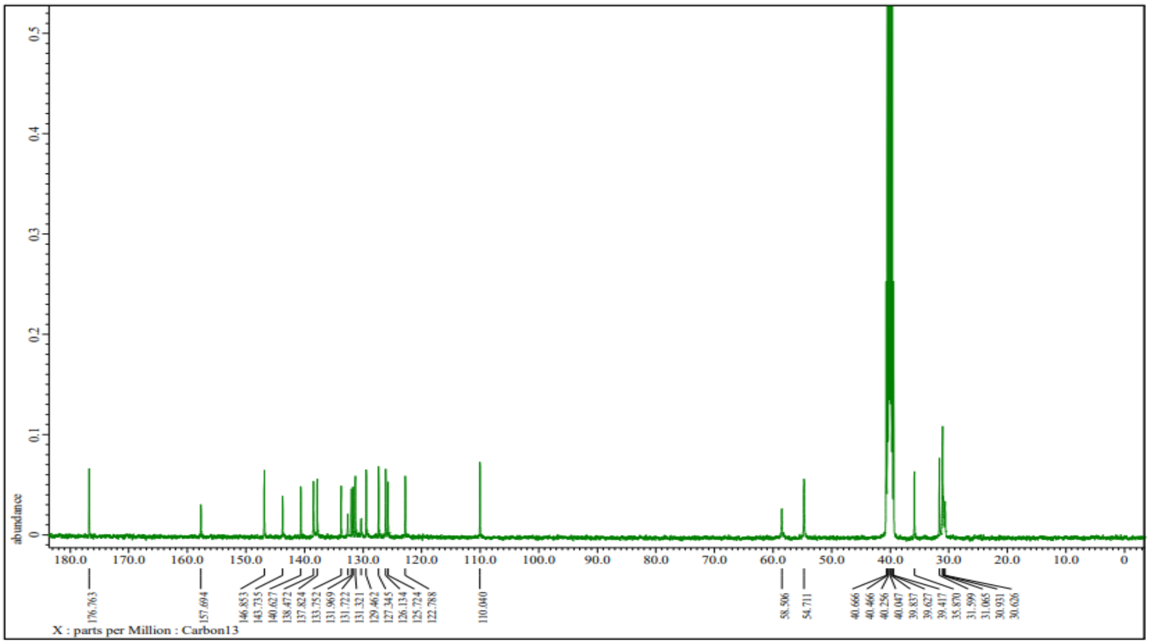

Supplementary Figure 39. NMR data of 6-chloro-5-(2-(4-(8-chloro-5,6-dihydro-11H-benzo[5,6]cyclohepta[1,2-b]pyridin-11-ylidene) piperidin-1-yl)ethyl)indolin-2-one (11a)

^{1}H NMR

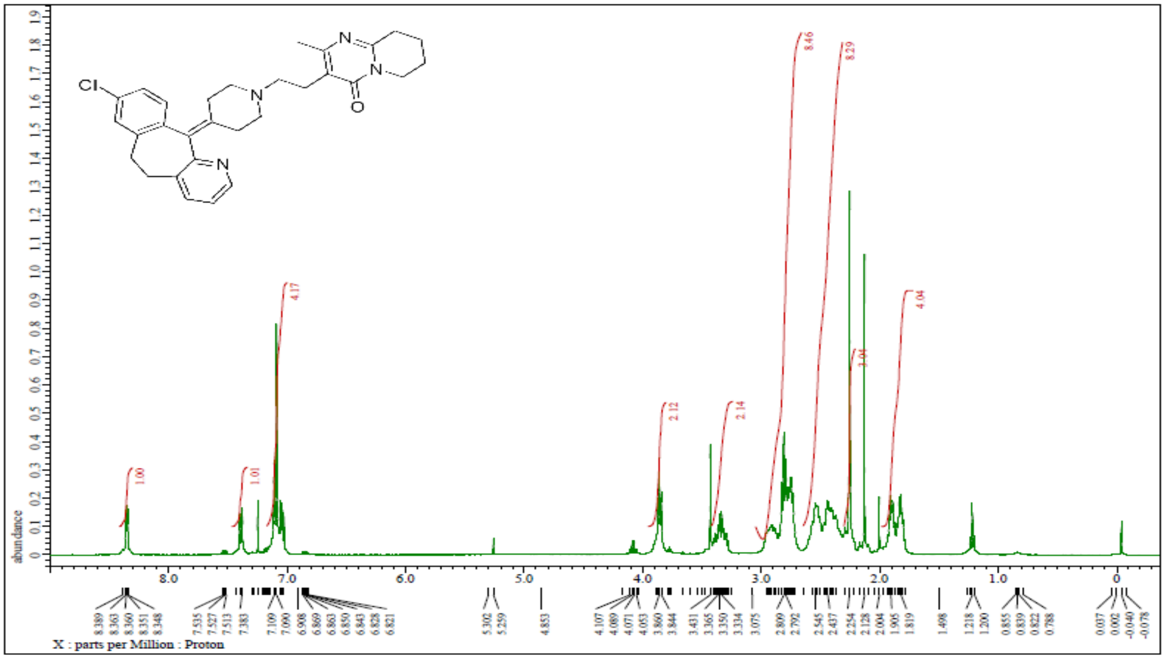

^{13}C NMR

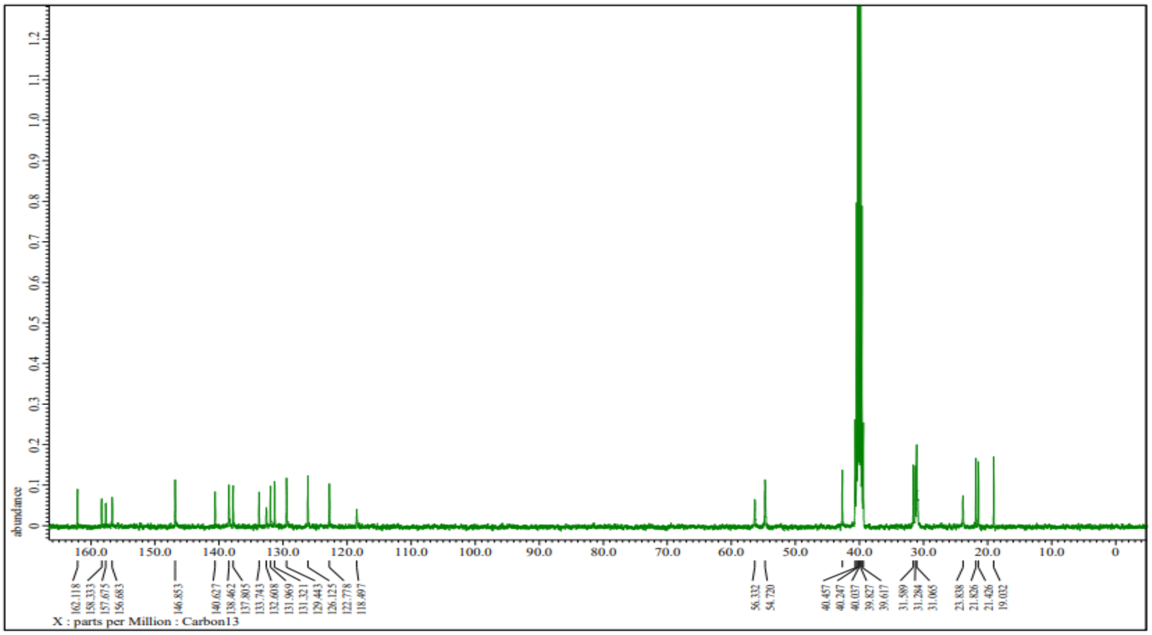

Supplementary Figure 40. NMR data of 3-(2-(4-(8-chloro-5,6-dihydro-11H-benzo[5,6]-cyclohepta[1,2-b]pyridin-11-ylidene)piperidin-1-yl)ethyl)-2-methyl-6,7,8,9-tetrahydro-4H-pyrido-[1,2-a]pyrimidin-4-one (11b)

^{1}H NMR

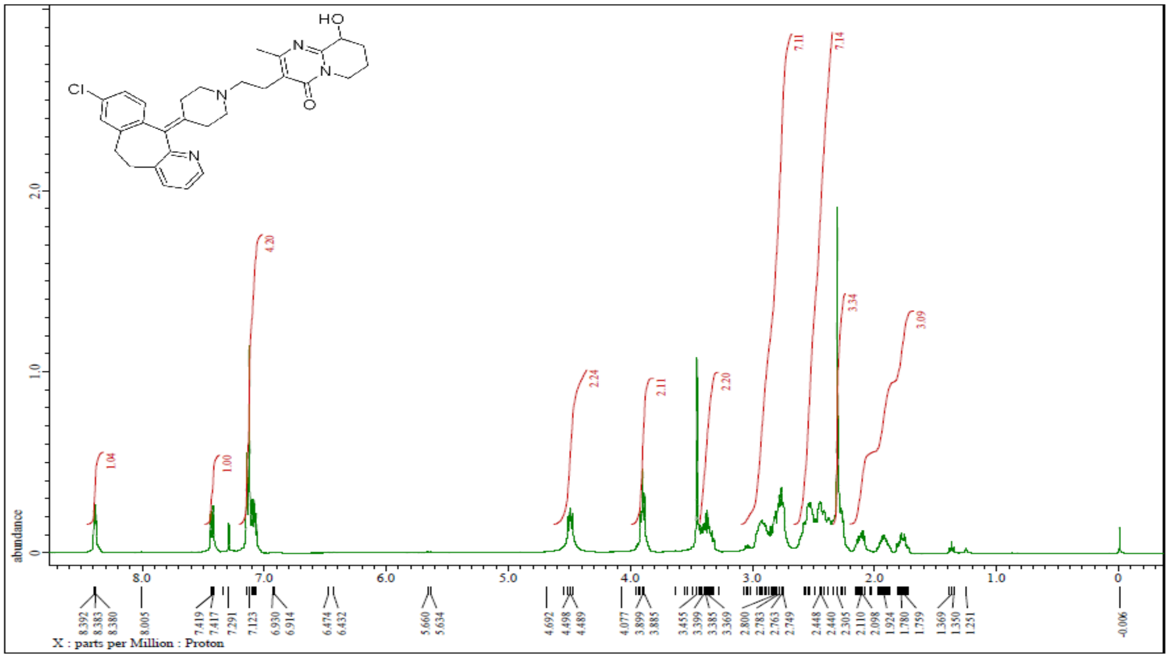

^{13}C NMR

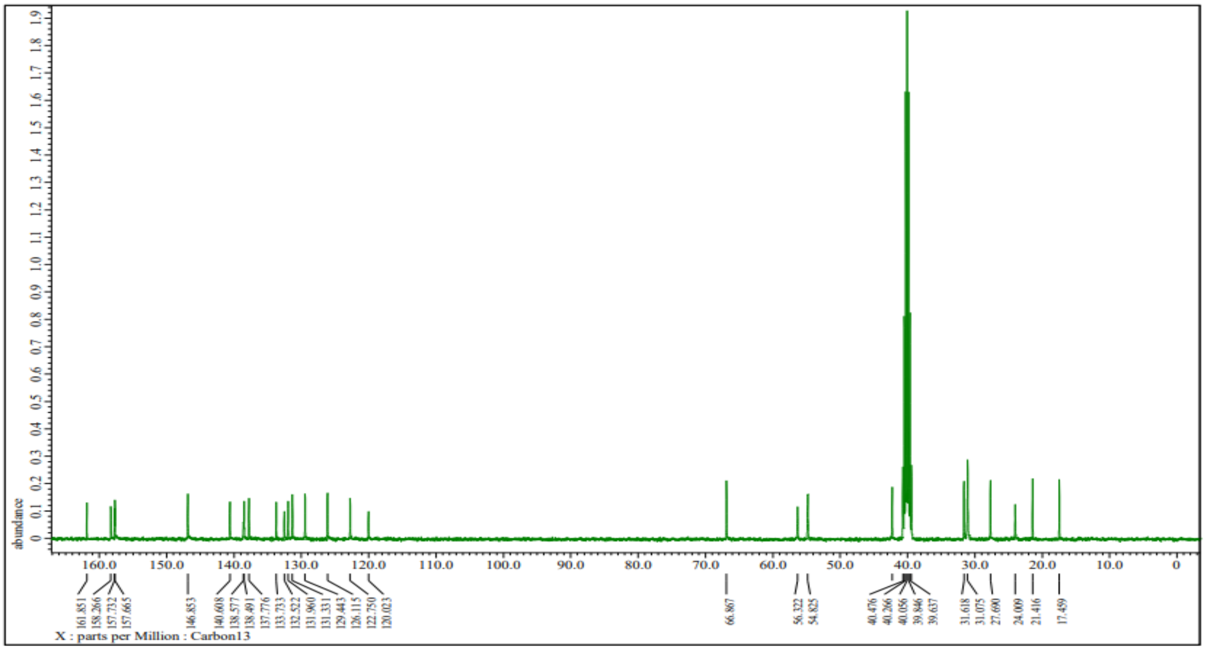

Supplementary Figure 41. NMR data of 3-(2-(4-(8-chloro-5,6-dihydro-11H-benzo[5,6]-cyclohepta[1,2-b]pyridin-11-ylidene)piperidin-1-yl)ethyl)-9-hydroxy-2-methyl-6,7,8,9-tetrahydro-4H-pyrido[1,2-a]pyrimidin-4-one (11c)

^{1}H NMR

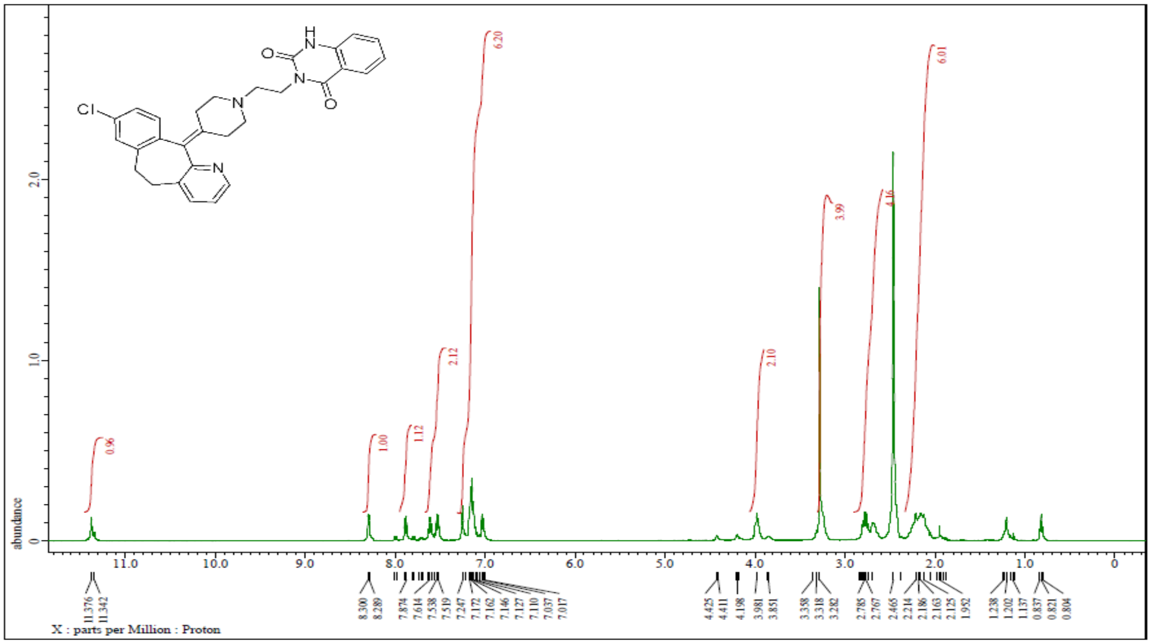

^{13}C NMR

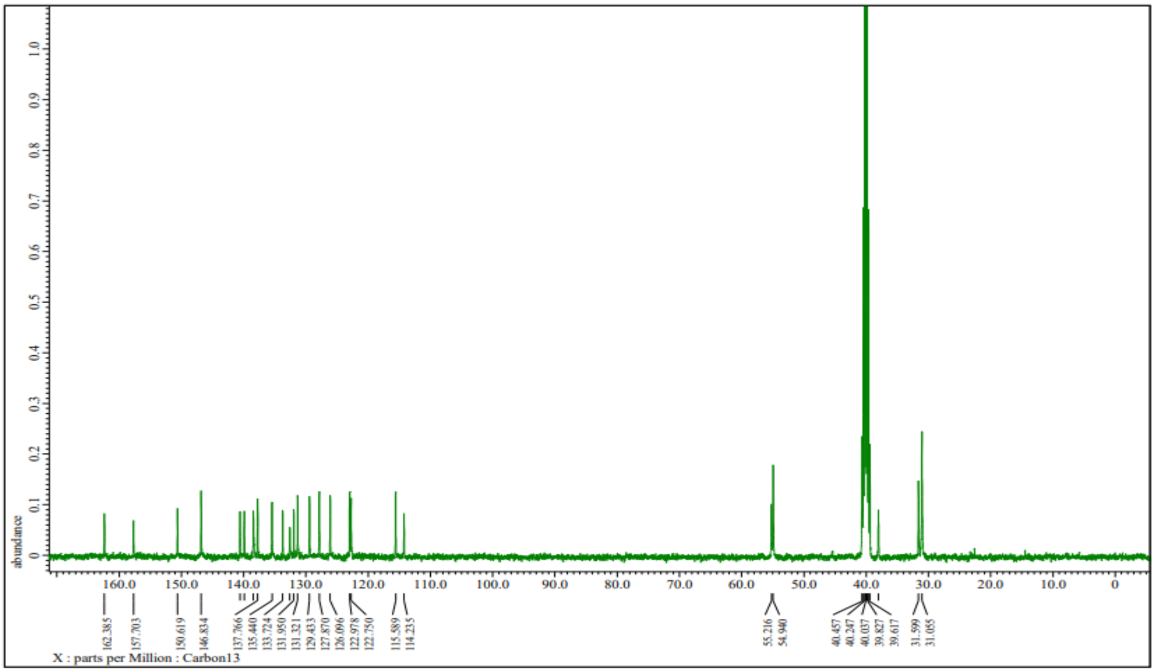

Supplementary Figure 42. NMR data of 3-(2-(4-(8-chloro-5,6-dihydro-11H-benzo[5,6]-cyclohepta[1,2-b]pyridin-11-ylidene)piperidin-1-yl)ethyl)quinazoline-2,4(1H,3H)-dione (11d)

^{1}H NMR

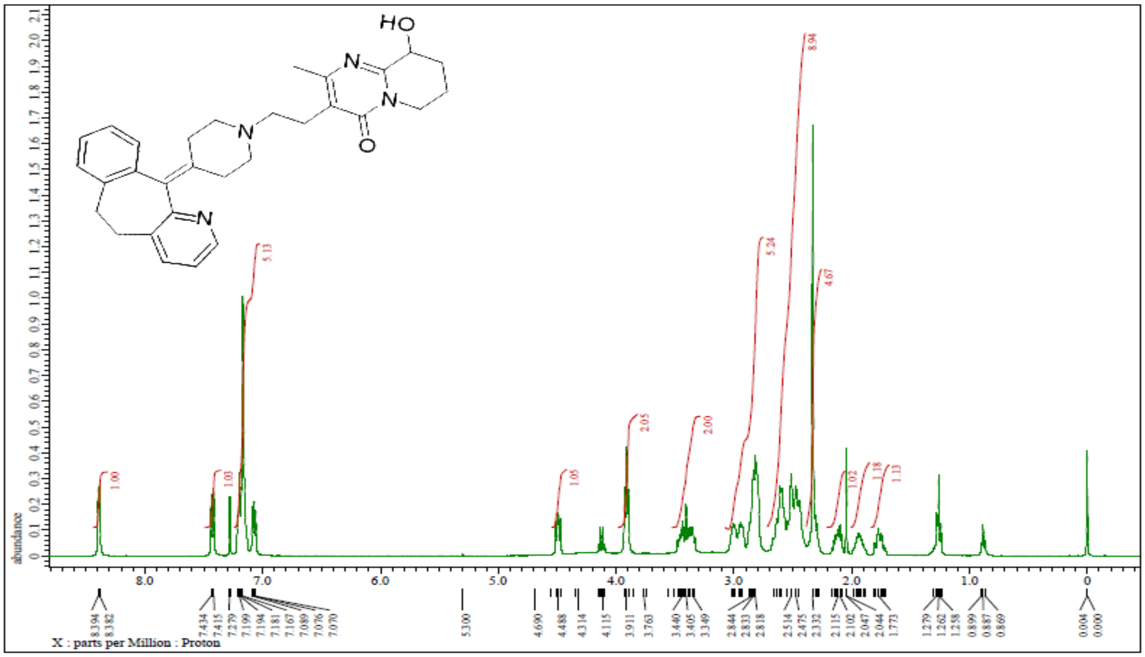

^{13}C NMR

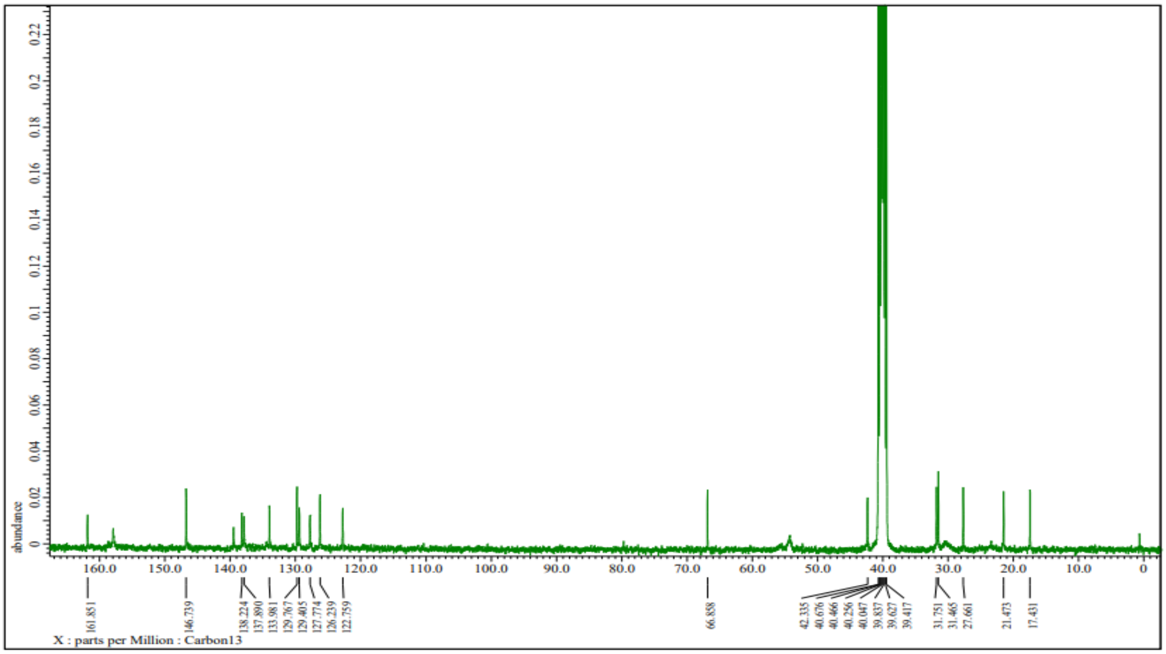

Supplementary Figure 43. NMR data of 3-(2-(4-(5,6-dihydro-11H-benzo[5,6]cyclohepta[1,2-b]pyridin-11-ylidene)piperidin-1-yl)ethyl)-9-hydroxy-2-methyl-6,7,8,9-tetrahydro-4H-pyrido[1,2-a]pyrimidin-4-one (11e)

^{1}H NMR

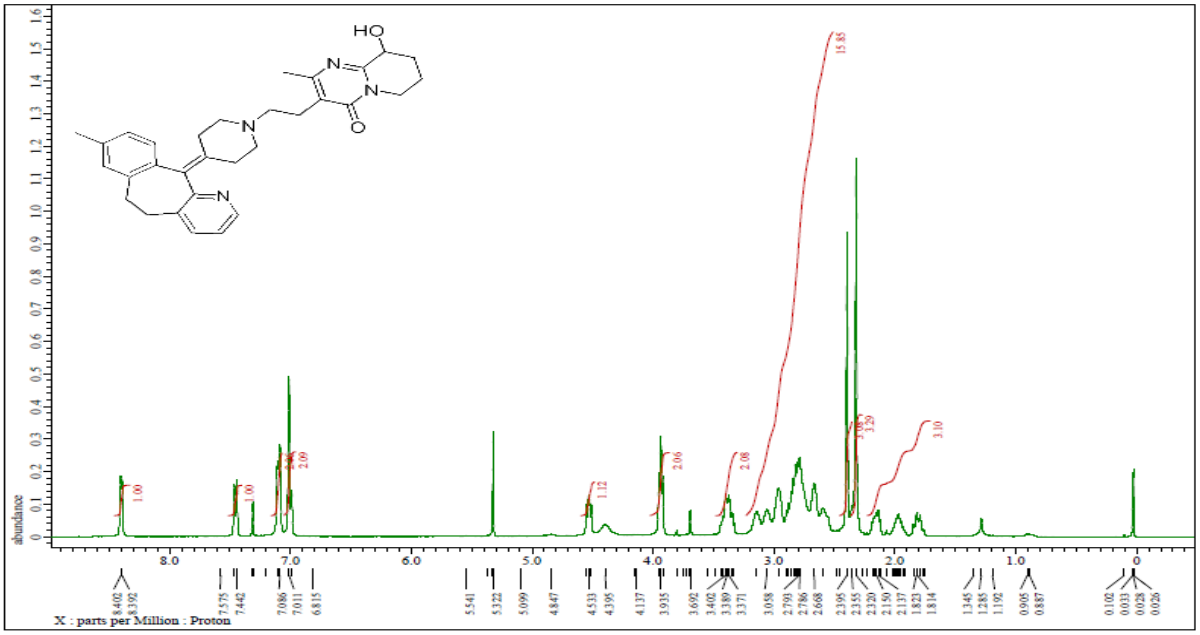

^{13}C NMR

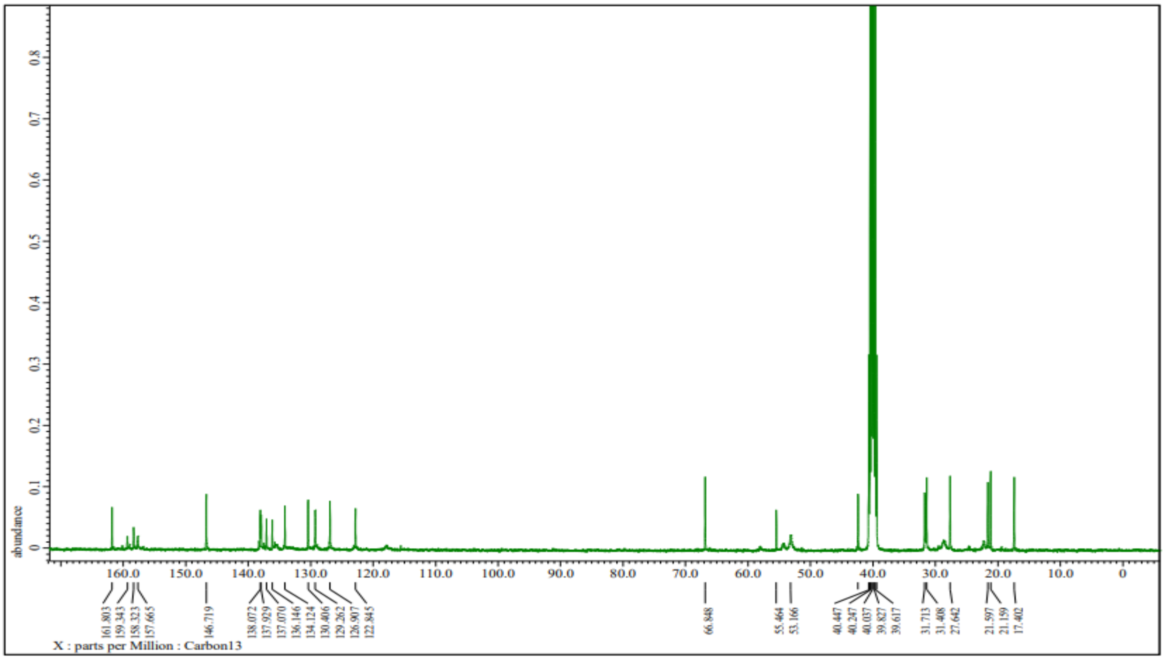

Supplementary Figure 44. NMR data of 9-hydroxy-3-(2-(4-(8-methyl-5,6-dihydro-11H-benzo[5,6] cyclohepta[1,2-b]pyridin-11-ylidene)piperidin-1-yl)ethyl)-2-methyl-6,7,8,9-tetrahydro-4H-pyrido-[1,2-a]pyrimidin-4-one (21)

^{1}H NMR

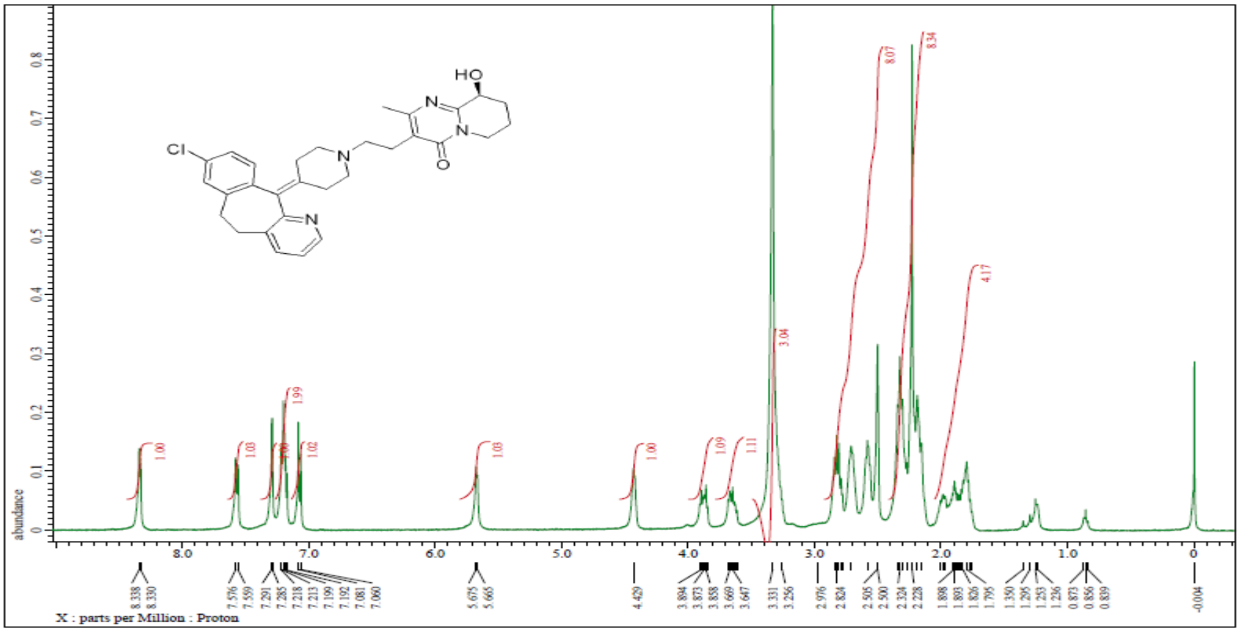

^{13}C NMR

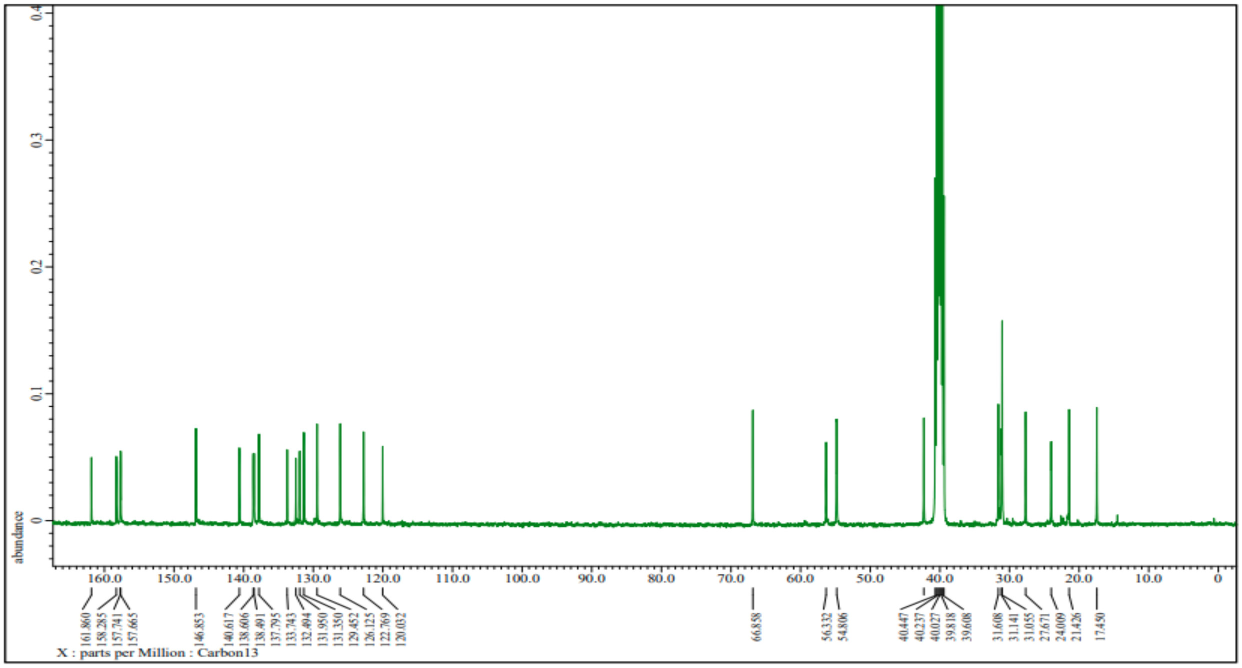

Supplementary Figure 45. NMR data of (S)-3-(2-(4-(8-chloro-5,6-dihydro-11H-benzo[5,6]cyclohepta[1,2-b]pyridin-11-ylidene) piperidin-1-yl)ethyl)-9-hydroxy-2-methyl-6,7,8,9-tetrahydro-4H-pyrido[1,2-a]pyrimidin-4-one (22f)

^{1}H NMR

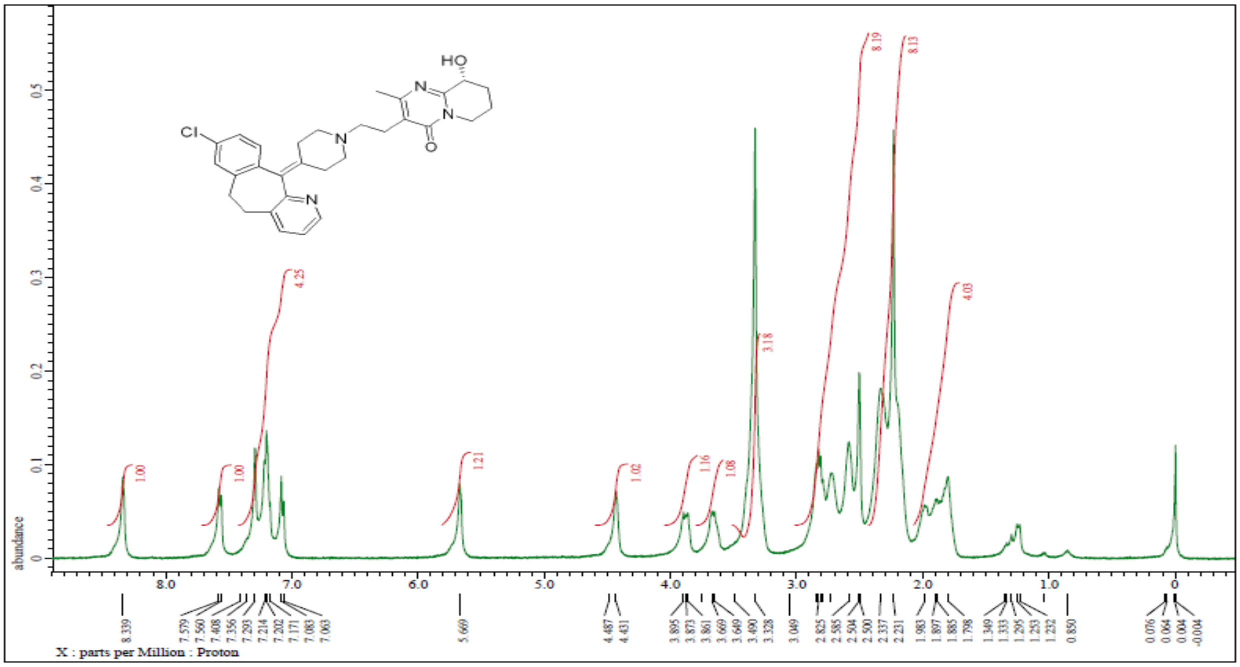

^{13}C NMR

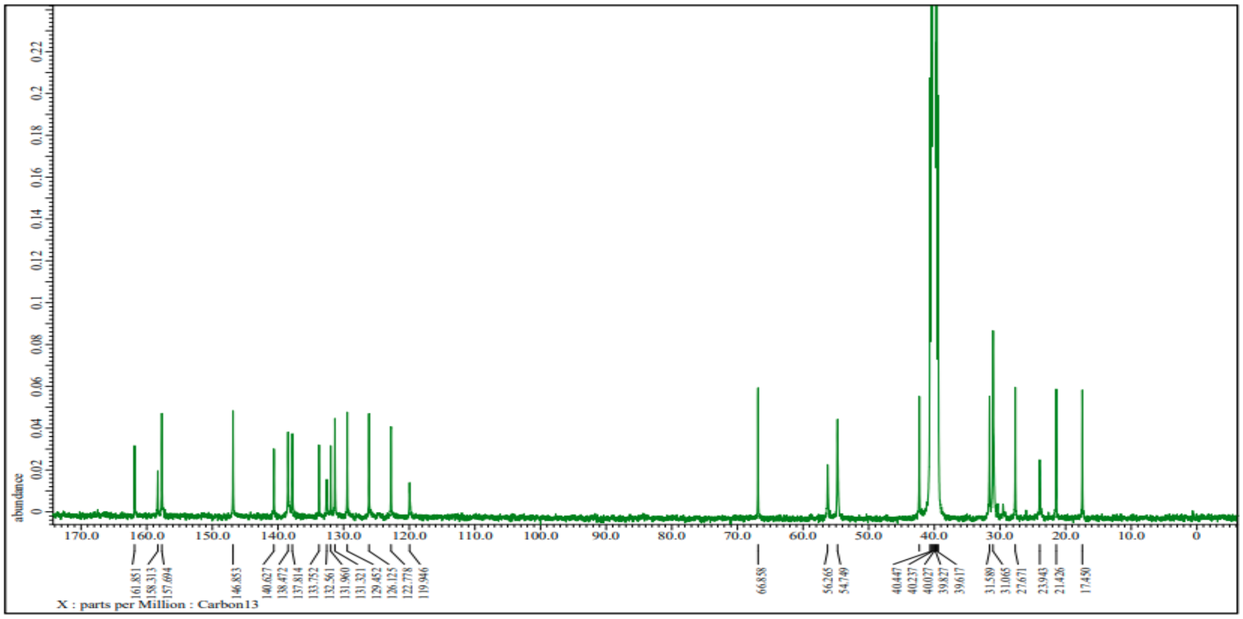

Supplementary Figure 46. NMR data of (R)-3-(2-(4-(8-chloro-5,6-dihydro-11H-benzo[5,6]cyclohepta[1,2-b]pyridin-11-ylidene) piperidin-1-yl)ethyl)-9-hydroxy-2-methyl-6,7,8,9-tetrahydro-4H-pyrido[1,2-a]pyrimidin-4-one (22g)

Supplementary Methods

General information

All solvents and chemicals were used as purchased without further purification. All the reported yields are isolated yields after column chromatography or crystallization. ^{1}H NMR spectra and ^{13}C spectra were recorded on a JEOL JNM-ECS400 spectrometers at 400 MHz for ^{1}H NMR and 100 MHz for ^{13}C NMR respectively, and optical rotations were obtained with a JASCO P-2000 polarimeter. The chemical shift (δ) is expressed in ppm relative to tetramethylsilane (TMS) as an internal standard, and $CDCl_3$, DMSO-d_6, CD_3OD were used as solvents. Multiplicity of peaks is expressed as s (singlet), d (doublet), t (triplet), q (quartet), dd (doublet of doublets), td (triplet of doublets), qd (quartet of doublets), dt (doublet of triplets), and m (multiplet). HRMS data were obtained by a JMS 700 (JEOL, Japan). Melting points were determined on a Melting Point M-560, purchased from Buchi. Optical rotations were measured on a P-2000 polarimeter, purchased from Jasco. High-performance liquid chromatography (HPLC) analyses were performed with a Waters Agilent HPLC system equipped with a PDA detector and an Agilent SB-C18 column (1.8 μm, 2.1 × 50 mm). The mobile phase consisted of buffer A (ultrapure H_2O containing 0.1% trifluoroacetic acid) and buffer B (chromatographic grade CH_3CN) for method.

Method

Time (min)	Water (%)	ACN (%)
0	95	5
2	95	5
8	0	100
15	0	100

Synthetic procedure.

8-Chloro-11-(piperidin-4-ylidene)-6,11-dihydro-5H-benzo[5,6]cyclohepta[1,2-b]pyridine (2)

A mixture of loratadine **1** (2 g, 5.2 mmol) and 10 ml of concentrated hydrochloric acid is stirred at reflux for 12 h. The excess of hydrochloric acid is evaporated, and the residue was dissolved in water. Adjusted pH 8 using ammonium hydroxide. Reaction mass was extracted with dichloromethane. Combined organic layer washed with water and brine, dried over anhydrous Na_2SO_4, and concentrated in vacuo to give 1.5 g of 8-chloro-11-(piperidin-4-ylidene)-6,11-dihydro-5H-benzo[5,6]cyclohepta[1,2-b]pyridine **2** in 92% yield as a cream white solid. ^{1}H NMR (400 MHz, DMSO-d_6): δ 8.32 (d, *J* =4.58 Hz, 1H), 7.56 (d, *J* =7.63 Hz, 1H), 7.28 (s, 1H), 7.23-7.15 (m, 2H), 7.06 (dd, *J* =8.24, 1.53 Hz, 1H), 3.46-3.21 (m, 3H), 2.93-2.75 (m, 4H), 2.65-2.53 (m, 1H), 2.34-2.04 (m, 4H); ^{13}C NMR (100 MHz, DMSO- d_6) δ 157.94, 146.82, 140.57, 139.60, 138.57, 137.70, 133.71, 131.97, 131.86, 131.35, 129.45, 126.05, 122.64, 48.31, 48.19, 32.79, 31.64, 31.06; mp 153-155 °C; IR (CH_2Cl_2) 3385 cm^{-1} ; LCMS [M+H] 311.1; HRMS (FAB) m/z calculated for $C_{19}H_{19}ClN_2$ [M + H]$^+$ 310.12, found 310.124; HPLC purity 99.88%.

1-(4-(8-Chloro-5,6-dihydro-11H-benzo[5,6]cyclohepta[1,2-b]pyridin-11-ylidene)piperidin-1-yl)ethan-1-one (3)

8-chloro-11-(piperidin-4-ylidene)-6,11-dihydro-5H-benzo[5,6]cyclohepta[1,2-b]pyridine **2** (100 mg, 0.322 mmol) was dissolved in 20 ml of dichloromethane. Acetyl chloride (25.25 mg, 0.322 mmol) and triethylamine (97.66 mg, 0.965 mmol) were sequentially added thereto. After completion of the reaction using brine, the reaction mixture was extracted twice with

dichloromethane. The collected organic layer was dried over anhydrous sodium sulfate and concentrated under reduced pressure to obtain a residue which was purified by column chromatography to obtain 91 mg of 1-(4-(8-chloro-5,6-dihydro-11H-benzo[5,6] cyclohepta[1,2-b]pyridin-11-ylidene)piperidin-1-yl)ethan-1-one **3** in 80% yield. ^{1}H NMR (400 MHz, DMSO-d_6): δ 8.34 (d, *J* =4.58 Hz, 1H), 7.57 (d, *J* =7.33 Hz, 1H), 7.31 (s, 1H), 7.25-7.17 (m, 2H), 7.13-7.06 (m, 1H), 3.84-3.72 (m, 1H), 3.67-3.53 (m, 1H), 3.39-3.19 (m, 3H), 3.18-3.05 (m, 1H), 2.88-2.76 (m, 2H), 2.45-2.09 (m, 4H), 1.99 (s, 3H); ^{13}C NMR (100 MHz, DMSO- d_6) δ 168.62, 157.34, 146.90, 140.73, 138.40, 138.03, 137.17, 133.90, 132.14, 131.24, 129.49, 126.23, 122.92, 47.01, 42.42, 31.48, 31.21, 31.08, 30.68, 21.85; mp 79-81 ^{o}C; IR (CH_2Cl_2) 1640 cm^{-1} ; LCMS [M+H] 353.12; HRMS (FAB) m/z calculated for $C_{21}H_{21}ClN_2O$ $[M + H]^+$ 352.13, found 352.134; HPLC purity 98.66%.

8-Chloro-11-(1-(methylsulfonyl)piperidin-4-ylidene)-6,11-dihydro-5H-benzo[5,6] cyclohepta[1,2-b]pyridine (4)

8-chloro-11-(piperidin-4-ylidene)-6,11-dihydro-5H-benzo[5,6]cyclohepta[1,2-b]pyridine **2** (100 mg, 0.322 mmol) was dissolved in 20 ml of dichloromethane. Methane sulfonyl chloride (40.53 mg, 0.354 mmol) and triethylamine (97.66 mg, 0.965 mmol) were sequentially added thereto. After completion of the reaction using brine, the reaction mixture was extracted twice with dichloromethane. The collected organic layer was dried over anhydrous sodium sulfate and concentrated under reduced pressure to obtain a residue which was purified by column chromatography to obtain 102 mg of 8-chloro-11-(1-(methylsulfonyl)piperidin-4-ylidene)-6,11-dihydro-5H-benzo[5,6]cyclohepta[1,2-b]pyridine **4** in 81% yield. ^{1}H NMR (400 MHz, DMSO-d_6):

δ 8.35 (d, *J* =4.58 Hz, 1H), 7.58 (d, *J* =7.79 Hz, 1H), 7.32 (s, 1H), 7.27-7.17 (m, 2H), 7.10 (d, *J* =8.24 Hz, 1H), 3.41-3.24 (m, 4H), 3.04-2.92 (m, 2H), 2.86 (s, 3H), 2.90-2.77 (m, 2H), 2.48-2.35 (m, 2H), 2.34-2.23 (m, 2H); ^{13}C NMR (100 MHz, DMSO- d_6) δ 157.08, 146.92, 140.74, 138.23, 138.10, 135.98, 134.40, 133.81, 132.27, 131.27, 131.16, 129.50, 126.26, 123.01, 47.14, 34.71, 31.44, 31.21, 31.09, 30.46, 30.31; mp 202-205 ^{o}C; IR (CH_2Cl_2) 1333 cm^{-1} ; LCMS [M+H] 389.1; HRMS (FAB) m/z calculated for $C_{20}H_{21}ClN_2O_2S$ $[M + H]^+$ 388.10, found 388.101; HPLC purity 98.78%.

4-(8-Chloro-5,6-dihydro-11H-benzo[5,6]cyclohepta[1,2-b]pyridin-11-ylidene)-N-isopropylpiperidine-1-carbothioamide (5)

8-chloro-11-(piperidin-4-ylidene)-6,11-dihydro-5H-benzo[5,6]cyclohepta[1,2-b]pyridine **2** (100 mg, 0.322 mmol) was dissolved in 20 ml of dichloromethane. Isopropyl isothiocyanate (34.18 mg, 0.338 mmol) and diisopropylethylamine (145.18 mg, 1.126 mmol) were sequentially added thereto. After completion of the reaction using brine, the reaction mixture was extracted twice with dichloromethane. The collected organic layer was dried over anhydrous sodium sulfate and concentrated under reduced pressure to obtain a residue which was purified by column chromatography to obtain 120 mg of 4-(8-chloro-5,6-dihydro-11H-benzo[5,6]cyclohepta[1,2-b]pyridin-11-ylidene)-N-isopropylpiperidine-1-carbothioamide **5** in 90% yield. ^{1}H NMR (400 MHz, DMSO-d_6): δ 8.34 (d, *J* =4.12 Hz, 1H), 7.58 (d, *J* =7.33 Hz, 1H), 7.31 (s, 1H), 7.25-7.17 (m, 3H), 7.12 (d, *J* =8.01 Hz, 1H), 4.59-4.45 (m, 1H), 4.17-3.99 (m, 2H), 3.56-3.42 (m, 2H), 3.38-3.22 (m, 2H), 2.90-2.76 (m, 2H), 2.56-2.32 (m, 2H), 2.25-2.11 (m, 2H), 1.12 (d, *J* = 6.41 Hz, 6H); ^{13}C NMR (100 MHz, DMSO- d_6) δ 180.24, 157.10, 146.67, 140.75, 138.31, 137.14, 133.91, 133.74,

132.15, 131.29, 129.47, 126.18, 125.43, 122.98, 48.12, 47.82, 47.65, 31.46, 31.11, 30.94, 30.39, 22.61; mp 92-94 °C; IR (CH_2Cl_2) 2968,1532 cm^{-1} ; LCMS [M+H] 412.1; HRMS (FAB) m/z calculated for $C_{23}H_{26}ClN_3S$ $[M + H]^+$ 411.15, found 411.154; HPLC purity 97.21%.

8-Chloro-11-(1-methylpiperidin-4-ylidene)-6,11-dihydro-5H-benzo[5,6]cyclohepta[1,2-b]pyridine (6)

Desloratadine **2** (100 mg, 0.261 mmol) was dissolved in 10 ml of methanol. formaldehyde (15.69 mg, 0.522 mmol) and NaBH4 (19.76 mg, 0.522 mmol) were sequentially added thereto. After completion of the reaction methanol was evaporated, and the residue was dissolved in water, the reaction mixture was extracted twice with ethyl acetate. The collected organic layer was dried over anhydrous sodium sulfate and concentrated under reduced pressure to obtain a residue which was purified by column chromatography to obtain 64 mg of 8-chloro-11-(1-methylpiperidin-4-ylidene)-6,11-dihydro-5H-benzo[5,6]cyclohepta[1,2-b]pyridine **6** in 75% yield. ^{1}H NMR (400 MHz, $CDCl_3$): δ 8.39 (d, *J* = 4.58 Hz, 1H), 7.40 (d, *J* = 7.63 Hz, 1H), 7.25-6.98 (m, 4H), 3.52-3.28 (m, 2H), 2.91-2.63 (m, 4H), 2.63-2.31 (m, 4H), 2.26 (s, 3H), 2.16-2.02 (m, 2H); ^{13}C NMR (100 MHz, DMSO- d_6) δ 157.69, 146.86, 140.61, 138.49, 137.82, 133.72, 132.78, 131.97, 131.30, 129.75, 129.46, 126.13, 122.78, 56.80, 56.74, 46.08, 31.58, 31.04, 30.94, 30.85; mp 106-108 °C; IR (CH_2Cl_2) 1465 cm^{-1} ; HRMS (FAB) m/z calculated for $C_{20}H_{21}ClN_2$ $[M + H]^+$ 324.14, found 324.143; HPLC purity 96.57%.

1-(4-Bromophenyl)-2-(4-(8-chloro-5,6-dihydro-11H-benzo[5,6]cyclohepta[1,2-b]pyridin-11-ylidene)piperidin-1-yl)ethan-1-one (8a)

8-chloro-11-(piperidin-4-ylidene)-6,11-dihydro-5H-benzo[5,6]cyclohepta[1,2-b]pyridine **2** (100 mg, 0.261 mmol) was dissolved in 5 ml of N,N-dimethylformamide . Sodium carbonate (108.05 mg, 0.782 mmol), potassium iodide (43.26 mg, 0.261 mmol) and 2-bromo-1-(4-bromophenyl) ethan-1-one **7a** (80 mg, 0.287 mmol) were sequentially added thereto while stirring. The reaction mixture was heated to 80°C. After completion of the reaction using brine, the reaction mixture was extracted twice with ethyl acetate. The collected organic layer was dried over anhydrous sodium sulfate and concentrated under reduced pressure to obtain a foamy residue which was purified by column chromatography to obtain the title compound 1-(4-bromophenyl)-2-(4-(8-chloro-5,6-dihydro-11H-benzo[5,6]cyclohepta[1,2-b]pyridin-11-ylidene)piperidin-1-yl)ethan-1-one **8a** (110 mg, 83%).^{1}H NMR (400 MHz, DMSO-d_6) :δ 8.33 (dd, J = 4.73, 1.53 Hz, 1H), 7.91 (d, J = 8.54 Hz, 2H), 7.72 (d, J = 8.54 Hz, 2H), 7.56 (d, J = 6.41 Hz, 1H), 7.29 (d, J = 2.14 Hz, 1H), 7.25-7.15 (m, 2H), 7.06 (d, J = 8.24 Hz, 1H), 3.82 (s, 2H), 2.88-2.76 (m, 4H), 2.42-2.10 (m, 8H); ^{13}C NMR (100 MHz, DMSO- d_6) δ 196.92, 157.67, 146.86, 140.58, 138.43, 137.90, 137.79, 135.33, 133.71, 132.78, 132.15, 131.98, 131.30, 130.69, 129.46, 127.79, 126.13, 122.77, 64.13, 54.74, 31.59, 31.06, 30.97; mp 143-146 °C; IR (CH_2Cl_2) 1682 cm^{-1} ; HRMS (FAB) m/z calculated for $C_{27}H_{24}BrClN_2O$ $[M + H]^+$ 506.08, found 506.076; HPLC purity 99.00%.

Compounds **8b** and **8c** were synthesized following the procedure given above. Their spectral data are as follows.

5-(2-(4-(8-Chloro-5,6-dihydro-11H-benzo[5,6]cyclohepta[1,2-b]pyridin-11-ylidene)piperidin-1-yl)acetyl)indolin-2-one (8b)

Yield: 84%; ^{1}H NMR (400 MHz, DMSO-d_6): δ 10.76 (s, 1H), 8.32 (d, J =4.58 Hz, 1H), 7.90 (d, J =8.01 Hz, 1H), 7.82 (s, 1H), 7.56 (d, J =7.79 Hz, 1H), 7.28 (s, 1H), 7.25-7.15 (m, 2H), 7.06 (d, J =7.10 Hz, 1H), 6.88 (d, J =8.01 Hz, 1H), 3.76 (s, 2H), 3.54 (s, 1H), 3.39-3.23 (m, 2H), 2.90-2.68 (m, 4H), 2.44-2.12 (m, 6H); ^{13}C NMR (100 MHz, DMSO- d_6) δ 195.93, 177.29, 157.67, 148.93, 146.86, 140.61, 138.45, 137.83, 133.75, 132.76, 131.98, 131.32, 130.01, 129.83, 129.48, 126.48, 126.15, 124.84, 122.80, 109.19, 63.82, 54.82, 36.05, 31.58, 31.21, 31.03, 30.90; LCMS [M+H] 484.1; mp 162-164 ^{o}C; IR (CH_2Cl_2) 3441, 1712, 1615 cm^{-1} ; HRMS (FAB) m/z calculated for $C_{29}H_{26}ClN_3O_2$ [M + H]$^{+}$ 483.17, found 483.171; HPLC purity 96.08%.

6-Chloro-5-(2-(4-(8-chloro-5,6-dihydro-11H-benzo[5,6]cyclohepta[1,2-b]pyridin-11-ylidene)piperidin-1-yl)acetyl)indolin-2-one (8c)

Yield: 86%; ^{1}H NMR (400 MHz, DMSO-d_6) :δ 10.78(s, 1H), 8.34-8.22 (m, 1H), 7.60-7.45 (m, 2H), 7.30-7.20(m, 1H), 7.20-7.15 (m, 2H), 7.05-6.95 (m, 1H), 6.85-6.80 (m, 1H), 3.70 (s, 2H), 3.53 (s, 2H), 3.40-3.20 (m, 2H), 2.90-2.60 (m, 4H), 2.40-2.24 (m, 4H), 2.22-2.06 (m, 2H); ^{13}C NMR (100 MHz, DMSO- d_6) δ 199.56, 177.01, 157.63, 147.67, 146.83, 140.57, 138.41, 137.79, 133.71, 132.80, 131.98, 131.29, 130.69, 130.52, 129.44, 126.11, 125.28, 122.28, 122.76, 111.04, 66.55, 54.55, 35.72, 31.58, 31.48, 31.05, 30.92, 22.59, 14.47; mp 149-151 ^{o}C; IR (CH_2Cl_2) 3440, 1711, 1621 cm^{-1} ; HRMS (FAB) m/z calculated for $C_{29}H_{25}C_{l2}N_3O_2$ [M + H]$^{+}$ 517.13, found 517.132; HPLC purity 95.89%.

1-(4-Bromophenyl)-2-(4-(8-chloro-5,6-dihydro-11H-benzo[5,6]cyclohepta[1,2-b]pyridin-11-ylidene)piperidin-1-yl)ethan-1-ol (9a)

A mixture of 1-(4-bromophenyl)-2-(4-(8-chloro-5,6-dihydro-11H-benzo[5,6]cyclohepta[1,2-b]pyridin-11-ylidene)piperidin-1-yl)ethan-1-one **8a** (50 mg , 0.098 mmol) and NaBH4 (7.45 mg, 0.197 mmol) in 10 ml of methanol is stirred at room temperature. After completion of the reaction methanol was evaporated, and the residue was dissolved in water, the reaction mixture was extracted twice with ethyl acetate. The collected organic layer was dried over anhydrous sodium sulfate and concentrated under reduced pressure to obtain a residue which was purified by column chromatography to obtain 35 mg of 1-(4-bromophenyl)-2-(4-(8-chloro-5,6-dihydro-11H-benzo[5,6]cyclohepta[1,2-b]pyridin-11-ylidene)piperidin-1-yl)ethan-1-ol **9a** in 70% yield . ^{1}H NMR (400 MHz, $CDCl_3$): δ 8.40 (d, *J* = 4.27 Hz, 1H), 7.49-7.39 (m, 3H), 7.28-7.20 (m, 2H), 7.20-7.06 (m, 4H), 4.74-4.64 (m, 1H), 3.47-3.29 (m, 2H), 3.27-2.18 (m, 12H); ^{13}C NMR (100 MHz, DMSO- d_6) δ 157.7, 146.88, 144.50, 140.53, 138.50, 137.84, 133.76, 131.99, 131.30, 129.50, 128.80, 125.15, 122.80, 120.28, 59.50, 66.16, 55.31, 55.03, 31.51, 31.05; mp 187-189 ^{o}C; IR (CH_2Cl_2) 3395, 1440 cm^{-1} ; HRMS (FAB) m/z calculated for $C_{27}H_{26}BrClN_2O$ [M + H]$^{+}$ 508.09, found 508.0992; HPLC purity 97.89%.

Compounds **9b** and **9c** were synthesized following the procedure given above. Their spectral data are as follows.

5-(2-(4-(8-Chloro-5,6-dihydro-11H-benzo[5,6]cyclohepta[1,2-b]pyridin-11-ylidene)piperidin-1-yl)-1-hydroxyethyl)indolin-2-one (9b)

Yield: 68%,^{1}H NMR (400 MHz, DMSO-d_6) :δ 10.28 (d, *J* = 8.85 Hz, 1H), 8.31 (d, *J* = 6.10 Hz, 1H), 7.56 (t, *J* = 7.93 Hz, 1H), 7.33-7.00 (m, 6H), 6.71 (t, *J* = 7.93 Hz, 1H), 4.90-4.82 (m, 1H), 4.66-4.55 (m, 1H), 3.42 (d, *J* = 8.54 Hz, 2H) , 2.87-2.64 (m, 4H), 2.39-2.07 (m, 6H); ^{13}C NMR

(100 MHz, DMSO- d_6) δ 177.00, 157.78, 146.84, 142.97, 140.59, 138.64, 138.51, 138.14, 137.80, 133.74, 132.40, 131.94, 131.35, 129.47, 126.12, 125.97, 125.69, 122.75, 108.96, 70.25, 66.82, 55.49, 55.07, 36.34, 31.61, 31.06; mp 163-165 ^oC; IR (CH_2Cl_2) 3416, 1702, 1439 cm^{-1} ; HRMS (FAB) m/z calculated for $C_{29}H_{28}ClN_3O_2$ $[M + H]^+$ 485.19, found 485.194; HPLC purity 99.01%.

6-Chloro-5-(2-(4-(8-chloro-5,6-dihydro-11H-benzo[5,6]cyclohepta[1,2-b]pyridin-11-ylidene)piperidin-1-yl)-1-hydroxyethyl)indolin-2-one (9c)

Yield: 72%,^{1}H NMR (400 MHz, DMSO-d_6) :δ 10.47-10.39 (m, 1H), 8.34 (d, J = 8.54 Hz, 1H), 7.64-7.52 (m, 1H), 7.44-7.02 (m, 5H), 6.79-6.72 (m, 1H), 5.18 (d, J = 7.93 Hz, 1H), 5.09-4.97 (m, 1H), 3.53-3.43 (m, 2H), 2.91-2.67 (m, 4H), 2.47-2.08 (m, 10H); ^{13}C NMR (100 MHz, DMSO- d_6) δ 176.91, 157.74, 146.84, 144.07, 140.58, 138.60, 138.51, 137.77, 134.80, 133.74, 132.45, 131.94, 131.34, 129.62, 129.45, 126.12, 125.65, 124.09, 122.75, 109.57, 66.98, 65.39, 55.51, 54.99, 35.98, 31.61, 31.06; mp 187-189 ^oC; IR (CH_2Cl_2) 3443, 1709, 1479 cm^{-1} ; HRMS (FAB) m/z calculated for $C_{29}H_{27}Cl_2N_3O_2$ $[M + H]^+$ 519.15, found 519.156; HPLC purity 92.17%.

6-Chloro-5-(2-(4-(8-chloro-5,6-dihydro-11H-benzo[5,6]cyclohepta[1,2-b]pyridin-11-ylidene) piperidin-1-yl)ethyl)indolin-2-one (11a)

8-chloro-11-(piperidin-4-ylidene)-6,11-dihydro-5H-benzo[5,6]cyclohepta[1,2-b]pyridine **2** (100 mg, 0.322 mmol) was dissolved in 5 ml of N,N-dimethylformamide . Sodium carbonate (68.20 mg, 0.644 mmol), potassium iodide (53.41 mg, 0.322 mmol) and 6-chloro-5-(2-chloroethyl)indolin-2-one **10a** (74 mg, 0.322 mmol) were sequentially added thereto while stirring. The reaction mixture was heated to 80°C. After completion of the reaction using brine, the reaction mixture was extracted twice with ethyl acetate. The collected organic layer was dried over

anhydrous sodium sulfate and concentrated under reduced pressure to obtain a foamy residue which was purified by column chromatography to obtain 125 mg of 6-chloro-5-(2-(4-(8-chloro-5,6-dihydro-11H-benzo[5,6]cyclohepta[1,2-b]pyridin-11-ylidene)piperidin-1-yl)ethyl)indolin-2-one **11a** in 77% yield. ^{1}H NMR (400 MHz, DMSO-d_6) :δ 10.36 (s, 1H), 8.30 (d, *J* = 4.58 Hz, 1H), 7.57 (d, *J* = 7.63 Hz, 1H), 7.31-7.26 (m, 1H), 7.24-7.15 (m, 3H), 7.07 (d, *J* = 8.24 Hz, 1H), 6.78 (s, 1H), 3.43 (s, 2H), 2.88-2.64 (m, 6H), 2.54-2.12 (m, 10H); ^{13}C NMR (100 MHz, DMSO- d_6) δ 176.76, 157.69, 146.85, 143.73, 140.62, 138.47, 137.82, 133.75, 132.60, 131.96, 131.72, 131.32, 130.32, 129.46, 127.34, 126.13, 125.72, 122.78, 110.04, 58.50, 54.71, 35.87, 31.59, 31.06, 30.93, 30.62; mp 159-161 ^{o}C; IR (CH_2Cl_2) 3197, 1709 cm^{-1} ; HRMS (FAB) m/z calculated for $C_{29}H_{27}Cl_2N_3O$ [M + H]$^{+}$ 503.15, found 503.152; HPLC purity 99.70%.

Compounds **11b-e** were synthesized following the procedure given above. Their spectral data are as follows.

3-(2-(4-(8-Chloro-5,6-dihydro-11H-benzo[5,6]cyclohepta[1,2-b]pyridin-11-ylidene)-piperidin-1-yl)ethyl)-2-methyl-6,7,8,9-tetrahydro-4H-pyrido[1,2-a]pyrimidin-4-one (11b)

(140 mg, 89%). ^{1}H NMR (400 MHz, CD_3OD): δ 8.49 (dd, *J* = 4.88, 1.22 Hz, 1H), 7.87 (d, *J* = 7.63 Hz, 1H), 7.50-7.44 (m, 1H), 7.38 (d, *J* = 2.14 Hz, 1H), 7.35-7.23 (m, 2H), 4.92-4.78 (m, 2H), 3.65-3.28 (m, 9H), 2.96-2.43 (m, 16H); ^{13}C NMR (100 MHz, DMSO- d_6) δ 162.11, 158.33, 157.67, 156.68, 146.85, 140.62, 138.46, 137.80, 133.74, 132.60, 131.96, 131.32, 129.44, 126.12, 122.77, 118.49, 56.33, 54.72, 42.68, 31.58, 31.28, 31.06, 23.83, 21.82, 21.42, 19.03; mp 125-127 ^{o}C; IR (CH_2Cl_2) 1651 cm^{-1} ; HRMS (FAB) m/z calculated for $C_{30}H_{33}ClN_4O$ [M + H]$^{+}$ 500.23, found 500.235; HPLC purity 100%.

3-(2-(4-(8-Chloro-5,6-dihydro-11H-benzo[5,6]cyclohepta[1,2-b]pyridin-11-ylidene)-piperidin-1-yl)ethyl)-9-hydroxy-2-methyl-6,7,8,9-tetrahydro-4H-pyrido[1,2-a]pyrimidin-4-one (11c)

(745 mg, 90%).^{1}H NMR (400 MHz, DMSO-d_6) :δ 8.30 (d, *J* = 4.58 Hz, 1H), 7.53 (d, *J* = 7.63 Hz, 1H), 7.26 (s, 1H), 7.21-7.12 (m, 2H), 7.04 (d, *J* = 7.93 Hz, 1H), 5.63 (d, *J* = 4.27 Hz, 1H), 4.43-4.34 (m, 1H), 3.89-3.78 (m, 1H), 3.67-3.55 (m, 1H), 3.29 (s, 3H), 3.35-3.20 (m, 2H), 2.86-2.42 (m, 6H), 2.40-2.04 (m, 10H), 1.98-1.68 (m, 2H); ^{13}C NMR (100 MHz, DMSO- d_6) δ 161.85, 158.26, 157.73, 157.66, 146.85, 140.60, 138.49, 137.77, 133.73, 132.52, 131.96, 131.33, 129.44, 126.11, 122.75, 120.02, 66.86, 56.32, 54.82, 42.28, 31.61, 31.07, 27.69, 24.00, 21.41, 17.45; mp 188-192 ^{o}C; IR (CH_2Cl_2) 3420, 1652 cm^{-1} ; LCMS [M+H] 517.2; HRMS (FAB) m/z calculated for $C_{30}H_{33}ClN_4O_2$ [M + H]$^{+}$ 516.23, found 516.229; HPLC purity 100%.

3-(2-(4-(8-Chloro-5,6-dihydro-11H-benzo[5,6]cyclohepta[1,2-b]pyridin-11-ylidene) piperidin-1-yl)ethyl)quinazoline-2,4(1H,3H)-dione (11d)

^{1}H NMR (400 MHz, DMSO-d_6) :δ 11.41 (s,1H), 8.33 (d, *J* = 4.27 Hz, 1H), 7.91 (d, *J* = 7.93 Hz, 1H), 7.64 (t, *J* = 7.93 Hz, 1H), 7.56 (d, *J* = 7.63 Hz, 1H), 7.33-7.11 (m, 5H), 7.06 (d, *J* = 8.24 Hz, 1H), 4.01 (t, *J* = 6.10 Hz, 2H), 3.39-3.21 (m, 2H), 2.90-2.64 (m, 4H), 2.37-2.06 (m, 8H); ^{13}C NMR (100 MHz, DMSO- d_6) δ 162.38, 157.70, 150.61, 146.83, 140.58, 139.89, 138.44, 137.76, 135.44, 133.72, 132.58, 131.95, 131.32, 129.43, 127.87, 126.09, 122.97, 122.75, 115.58, 114.23, 55.21, 54.94, 38.03, 31.59, 31.05; mp 133-135 ^{o}C; IR (CH_2Cl_2) 1716, 1662 cm^{-1} ; LCMS [M+H] 517.2;

HRMS (FAB) m/z calculated for $C_{29}H_{27}ClN_4O_2$ [M + H] $^+$ 498.18, found 498.182; HPLC purity 99.65%.

3-(2-(4-(5,6-Dihydro-11H-benzo[5,6]cyclohepta[1,2-b]pyridin-11-ylidene)piperidin-1-yl)ethyl)-9-hydroxy-2-methyl-6,7,8,9-tetrahydro-4H-pyrido[1,2-a]pyrimidin-4-one (11e)

^{1}H NMR (400 MHz, $CDCl_3$): δ 8.38 (d, *J* = 4.88 Hz, 1H), 7.42 (d, *J* = 7.63 Hz, 1H), 7.23-7.12 (m, 4H), 7.10-7.04 (m, 1H), 4.52-4.45 (m, 1H), 3.90 (t, *J* = 6.71 Hz, 2H), 3.50-3.30 (m, 2H), 3.05-2.76 (m, 6H), 2.71-2.26 (m, 10H), 2.32 (s, 3H), 2.18-2.06 (m, 1H), 2.0-1.86 (m, 1H), 1.82-1.70 (m, 1H); ^{13}C NMR (100 MHz, DMSO- d_6) δ 161.85, 158.0, 146.73, 139.5, 138.22, 137.89, 133.98, 129.76, 129.40, 127.77, 126.23, 122.75, 66.85, 42.33, 31.75, 31.46, 27.66, 21.47, 17.43; mp 115-118 ^{o}C; IR (CH_2Cl_2) 3423, 1648 cm^{-1} ; HRMS (FAB) m/z calculated for $C_{30}H_{34}N_4O_2$ [M + H] $^+$ 482.27, found 482.268; HPLC purity 95.74%.

9-Hydroxy-3-(2-(4-(8-methyl-5,6-dihydro-11H-benzo[5,6] cyclohepta[1,2-b]pyridin-11-ylidene)piperidin-1-yl)ethyl)-2-methyl-6,7,8,9-tetrahydro-4H-pyrido[1,2-a]pyrimidin-4-one (21)

N-(tert-Butyl)-3-methylpicolinamide (14)

2-Cyano-3-methylpyridine **13** (1 g, 8.465 mmol) and acetic acid (1.662 g 27.679 mmol, 1.58 mL) were stirred at room temperature while concentrated sulfuric acid (1.868 g, 19.045 mmol, 1.01 mL) was added over 0.5 h. During the addition, the solution was first an opaque, white solution and then became clear and colorless by the end of the addition. tert- Butyl acetate (1.966 g, 16.929 mmol, 2.271 mL) was added over 45 min under nitrogen atmosphere. After addition, the resulting

solution was stirred at rt for 12 h. The reaction was quenched by addition into aqueous NaOH solution, the solid was collected by filtration and dried under vacuum to afford 1.5 g of **14** in 92% yield as a white crystalline solid

N-(tert-Butyl)-3-(3-methylphenethyl) picolinamide (15)

To a solution of N-(tert-butyl)-3-methylpicolinamide **14** (1.5 g, 7.802 mmol) in THF (50 mL) under Nitrogen atmosphere at -40° C was added n-BuLi (2.5 M in hexane, 1.025 g, 6.4 mL, 15.994) and then NaBr (80.28 mg, 0.780 mmol). The solution was allowed to stir for 30 min before the addition of a solution of 1-(chloromethyl)-3-methylbenzene (1.207 g, 8.582 mmol) in THF (5 mL). The reaction was quenched after 1.5 h by the addition of water and then allowed to warm to room temperature and extracted with EtOAc. The organic phase was dried over Na_2SO_4, filtered, and concentrated to afford 1.2 g of **15** in 52% yield

3-(3-Methylphenethyl) picolinonitrile (16)

A solution of N-(tert-butyl)-3-(3-methylphenethyl) picolinamide **15** (1.2 g, 4.048 mmol) in 4 mL (6.207 g, 40.484 mmol) of phosphorous oxychloride was heated at reflux for 3 h. Excess phosphorus oxychloride was removed by distillation and the remaining solution was carefully poured into ice water. The pH of the solution was adjusted to 8 with 50% aqueous sodium hydroxide. The product was collected by filtration, washed with water, and dried under vacuum to afford 760 mg of **16** in 84% yield

(3-(3-Methylphenethyl)pyridin-2-yl)(1-methylpiperidin-4-yl)methanone (17)

Magnesium turnings (124.62 mg, 5.128 mmol) and iodine (18 mg, 0.068 mmol) in THF (50 ml) were Stirred at 50-60°C. 4-Chloro-N-methyl piperdine (685.26 mg, 5.128 mmol) was added slowly. The reaction mass was refluxed and maintain for 4.0 hours at 65-75°C. The reaction mass was cooled to 25-40°C. Further, 3-(3-methylphenethyl)picolinonitrile **16** (760 mg, 3.419 mmol) was added and the reaction mass was continued stirring for 6.0 hours at 25-40°C followed by addition of water (50 mL) and HCl (2 mL). The reaction mass was stirred for 1.0 hour at 25-40°C. THF was distilled from aqueous layer and sodium hydroxide solution was added to adjust pH 4. the reaction mixture was extracted twice with ethyl acetate. The collected organic layer was dried over anhydrous sodium sulfate and concentrated under reduced pressure to obtain a residue which was purified by column chromatography to afford 680 mg of **17** in 62% yield.

8-Methyl-11-(1-methylpiperidin-4-ylidene)-6,11-dihydro-5H-benzo[5,6]cyclohepta[1,2-b]pyridine (18)

(3-(3-methylphenethyl) pyridin-2-yl)(1-methylpiperidin-4-yl)methanone **17** (680 mg, 2.109 mmol) in trifluoromethanesulfonic acid (3 ml, 4.447 g, 31.633 mmol) at 90°-95° C for 18 hours under nitrogen. Cool the reaction and quench the reaction with ice-water and adjust the pH to 6 with NaOH. Extract die product with ethyl acetate and the organic layer was wash with brine. The solution was concentrated under reduced pressure to obtain a residue which was purified by column chromatography to afford 550 mg of **18** in 85% yield.

Ethyl 4-(8-methyl-5,6-dihydro-11H-benzo[5,6]cyclohepta[1,2-b]pyridin-11-ylidene)-piperidine-1-carboxylate (19)

Ethyl chloroformate (0.5 mL; 588.16 mg; 5.420 mmol) was added slowly to a hot (-80° C) toluene solution (50 mL) of the 8-methyl-11-(1-methylpiperidin-4-ylidene)-6,11-dihydro-5H-benzo[5,6]cyclohepta[1,2-b]pyridine **18** (550 mg, 1.807 mmol). Following complete addition, the temperature was maintained at 80° C. for 1 hr. The reaction mixture was cooled to ambient temperature and the toluene solution washed with water which was adjusted to pH 10 with aqueous sodium hydroxide. The organic layer was concentrated to a residue which was dissolved in hot acetonitrile and decolorized with charcoal. The solution was concentrated under reduced pressure to obtain a residue which was purified by column chromatography to afford 400 mg of **19** in 61% yield. ^{1}H NMR (400 MHz, $CDC1_3$) 61.25 (t, 3H, *J* =8 Hz), 2.3-2.4 (m, 3H), 2.4-2.5 (m,1H), 2.7-2.9 (m,2H), 3.1-3.2 (m,2H), 3.3- 3.4 (m,2H), 3.81 (br s, 2H), 4.13 (q, 2H, *J* = 8 Hz), 7.1-7.3 (m,4H), 7.43 (dd, 1H , *J* =9,2 Hz), 8.40 (d, 1H, *J* =5 Hz).

8-Methyl-11-(piperidin-4-ylidene)-6,11-dihydro-5H-benzo[5,6]cyclohepta[1,2-b]pyridine (20)

A mixture of ethyl 4-(8-methyl-5,6-dihydro-11H-benzo[5,6]cyclohepta[1,2-b]pyridin-11-ylidene)piperidine-1-carboxylate **19** (400 mg, 1.104 mmol) and 10 ml of concentrated hydrochloric acid is stirred at reflux for 12 h. The excess of hydrochloric acid is evaporated, and the residue was dissolved in water. Adjusted pH 8 using ammonium hydroxide. Reaction mass was extracted with dichloromethane. Combined organic layer washed with water and brine, dried over anhydrous Na_2SO_4, and concentrated in vacuo to give 250 mg of 8-chloro-11-(piperidin-4-ylidene)-6,11-dihydro-5H-benzo[5,6]cyclohepta[1,2-b]pyridine **20** in 78% yield as a cream white solid.

9-Hydroxy-3-(2-(4-(8-methyl-5,6-dihydro-11H-benzo[5,6]cyclohepta[1,2-b]pyridin-11-ylidene)piperidin-1-yl)ethyl)-2-methyl-6,7,8,9-tetrahydro-4H-pyrido[1,2-a]pyrimidin-4-one (21)

8-methyl-11-(piperidin-4-ylidene)-6,11-dihydro-5H-benzo[5,6]cyclohepta[1,2-b]pyridine (**20**) (100 mg, 0.344 mmol) was dissolved in 5 ml of N,N-dimethylformamide . Sodium carbonate (73 mg, 0.689 mmol), potassium iodide (57.16 mg, 0.344 mmol) and 3-(2-chloroethyl)-9-hydroxy-2-methyl-6,7,8,9-tetrahydro-4H-pyrido[1,2-a] pyrimidin-4-one (**10c**) (84 mg, 0.344 mmol) were sequentially added thereto while stirring. The reaction mixture was heated to 80°C. After completion of the reaction using brine, the reaction mixture was extracted twice with ethyl acetate. The collected organic layer was dried over anhydrous sodium sulfate and concentrated under reduced pressure to obtain a foamy residue which was purified by column chromatography to obtain 130 mg of **21** in 76% yield. ^{1}H NMR (400 MHz, $CDCl_3$): δ 8.37 (d, *J* = 4.27 Hz, 1H), 7.42 (d, *J* = 7.63 Hz, 1H), 7.11-7.04 (m, 2H), 7.01-6.94 (m, 2H), 4.54-4.47 (m, 1H), 3.90 (t, *J* = 6.71 Hz, 2H), 3.44-3.28 (m, 2H), 3.20-2.50 (m, 14H), 2.36 (s, 3H), 2.29 (s, 3H), 2.41-2.25 (m, 2H), 2.21-2.07 (m, 1H), 2.01-1.87 (m, 1H), 1.85-1.71 (m, 1H); ^{13}C NMR (100 MHz, DMSO- d_6) δ 161.80, 159.34, 158.32, 157.66, 146.71, 138.07, 137.92, 137.07, 136.14, 134.12, 130.40, 129.26, 126.90, 122.84, 66.84, 55.46, 53.16, 42.38, 31.71, 31.40, 24.64, 21.59, 21.15, 17.40; mp 99-102 ^{o}C; IR (CH_2Cl_2) 3416, 1651 cm^{-1} ; HRMS (FAB) m/z calculated for $C_{31}H_{36}N_4O_2$ [M + H]$^+$ 496.28, found 496.284; HPLC purity 98.85%.

(S)-3-(2-Chloroethyl)-2-methyl-4-oxo-6,7,8,9-tetrahydro-4H-pyrido[1,2-a] pyrimid in-9-yl (1R,4S)-4,7,7-trimethyl-3-oxo-2-oxabicyclo[2.2.1]heptane-1-carboxylate (10f)

(+)-**10f** was synthesized by using reported procedure of Xu et al.[1.] ^{1}H NMR (400 MHz, Chloroform-*d*) δ 6.02 – 5.81 (m, 1H), 4.10 – 3.80 (m, 2H), 3.80 – 3.63 (m, 2H), 3.06 – 2.88 (m, 2H), 2.56 – 2.37 (m, 1H), 2.28 (s, 3H), 2.23 – 1.82 (m, 6H), 1.76 – 1.61 (m, 1H), 1.16 – 0.91 (m, 9H). $[\alpha]_D^{24.96}$ = +63.39° (c 0.2, methanol). Rt (Normal phase HPLC) = 7.451 min

(R)-3-(2-Chloroethyl)-2-methyl-4-oxo-6,7,8,9-tetrahydro-4H-pyrido[1,2-a]pyrimidin-9-yl (1R,4S)-4,7,7-trimethyl-3-oxo-2-oxabicyclo[2.2.1]heptane-1-carboxylate (10g)

(-)-**10g** was synthesized by reported procedure of Xu et al.[1.] ^{1}H NMR (400 MHz, Chloroform-*d*) δ 5.98 – 5.86 (m, 1H), 4.08 – 3.82 (m, 2H), 3.78 – 3.69 (m, 2H), 3.03 – 2.91 (m, 2H), 2.55 – 2.37 (m, 1H), 2.29 (s, 3H), 2.23 – 1.84 (m, 6H), 1.85- 1.69 (m, 1H), 1.17 – 0.97 (m, 9H). $[\alpha]_D^{22.77}$ = -67.68° (c 0.2, methanol). Rt (Normal phase HPLC) = 7.459 min

Compounds **11f** and **11g** were synthesized following the procedure of compound **11a** by using the intermediates (S)-3-(2-chloroethyl)-2-methyl-4-oxo-6,7,8,9-tetrahydro-4H-pyrido[1,2-a] pyrimidin-9-yl (1R,4S)-4,7,7-trimethyl-3-oxo-2-oxabicyclo[2.2.1]heptane-1-carboxylate (**10f)** and (R)-3-(2-chloroethyl)-2-methyl-4-oxo-6,7,8,9-tetrahydro-4H-pyrido[1,2-a]pyrimidin-9-yl (1R,4S)-4,7,7-trimethyl-3-oxo-2-oxabicyclo[2.2.1]heptane-1-carboxylate (**10g**).

(S)-3-(2-(4-(8-Chloro-5,6-dihydro-11H-benzo[5,6]cyclohepta[1,2-b]pyridin-11-ylidene) piperidin-1-yl)ethyl)-9-hydroxy-2-methyl-6,7,8,9-tetrahydro-4H-pyrido[1,2-a]pyrimidin-4-one (22f)

(S)-3-(2-(4-(8-chloro-5,6-dihydro-11H-benzo[5,6]cyclohepta[1,2-b]pyridin-11-ylidene)piperidin-1-yl)ethyl)-2-methyl-4-oxo-6,7,8,9-tetrahydro-4H-pyrido[1,2-a]pyrimidin-9-yl (1S,4R)-4,7,7-trimethyl-3-oxo-2-oxabicyclo[2.2.1]heptane-1-carboxylate (**11f**) (50 mg, 0.072 mmol) was dissolved in 5 ml of methanol. *i*Pr_2NH (0.2 ml, 1.434 mmol) was added thereto while stirring. The reaction mixture was stirred for 6 hours at room temperature. After completion of the reaction, the solvent was concentrated under reduced pressure to obtain a crude residue which was purified by column chromatography to afford 37 mg of (+)-**22f** in 98% yield and chiral HPLC showed ee >99%. ^{1}H NMR (400 MHz, DMSO-d_6) δ 8.29 (dd, J = 4.8, 1.7 Hz, 1H), 7.53 (dd, J = 7.8, 1.7 Hz, 1H), 7.25 (d, J = 2.4 Hz, 1H), 7.22 – 7.09 (m, 2H), 7.03 (d, J = 8.2 Hz, 1H), 5.63 (d, J = 4.8 Hz, 1H), 4.49 – 4.29 (m, 1H), 3.92 – 3.76 (m, 1H), 3.71 – 3.53 (m, 1H), 3.31 (s, 3H), 2.88 – 2.72 (m, 2H), 2.73 – 2.61 (m, 2H), 2.61 – 2.50 (m, 2H), 2.50 – 2.40 (m, 2H), 2.35 – 2.17 (m, 8H), 2.00 – 1.70 (m, 4H). ^{13}C NMR (101 MHz, DMSO-d_6) δ 161.88, 158.30, 157.68, 146.87, 140.63, 138.62, 138.50, 137.81, 133.76, 132.51, 131.96, 131.36, 129.47, 126.14, 122.79, 120.04, 66.87, 56.34, 54.85, 54.80, 42.30, 31.61, 31.15, 31.06, 27.68, 24.01, 21.42, 17.45. IR (CH_2Cl_2) 3383, 1651 cm^{-1} ; mp 188-192 ^{o}C; $[\alpha]_D^{24.5}$ = +24.57^{o} (c 0.2, ethanol). Rt (Chiral HPLC) = 15.367 min.

(R)-3-(2-(4-(8-Chloro-5,6-dihydro-11H-benzo[5,6]cyclohepta[1,2-b]pyridin-11-ylidene) piperidin-1-yl)ethyl)-9-hydroxy-2-methyl-6,7,8,9-tetrahydro-4H-pyrido[1,2-a]pyrimidin-4-one (22g)

(R)-3-(2-(4-(8-chloro-5,6-dihydro-11H-benzo[5,6]cyclohepta[1,2-b]pyridin-11-ylidene) piperidin -1-yl)ethyl)-2-methyl-4-oxo-6,7,8,9-tetrahydro-4H-pyrido[1,2-a]pyrimidin-9-yl (1S,4R)-4,7,7-trimethyl-3-oxo-2-oxabicyclo[2.2.1]heptane-1-carboxylate (**11g**) (Compound **11g** was synthesized by reported procedure Xu et al.) (50 mg, 0.072 mmol) was dissolved in 5 ml of methanol . *i*Pr_2NH (0.2 ml, 1.434 mmol) was added thereto while stirring. The reaction mixture was stirred for 6 hours at room temperature. After

completion of the reaction, the solvent was concentrated under reduced pressure to obtain a crude residue which was purified by column chromatography to afford 36 mg of (-)-**22g** in 97% yield and chiral HPLC showed ee >99%. ^{1}H NMR (400 MHz, DMSO-d_6) δ 8.29 (d, *J* = 4.8 Hz, 1H), 7.52 (d, *J* = 7.8 Hz, 1H), 7.36 – 6.97 (m, 4H), 5.62 (d, *J* = 4.8 Hz, 1H), 4.49 – 4.30 (m, 1H), 3.95 – 3.74 (m, 1H), 3.72 – 3.51 (m, 1H), 3.36 (s, 3H), 2.90 – 2.46 (m, 8H), 2.36 – 2.17 (m, 8H), 2.01 – 1.72 (m, 4H). ^{13}C NMR (101 MHz, DMSO-d_6) δ 161.87, 158.33, 157.71, 146.87, 140.64, 138.48, 137.82, 133.76, 132.58, 131.98, 131.34, 129.46, 126.14, 122.80, 119.96, 66.87, 56.26, 54.75, 42.30, 31.60, 31.06, 27.68, 23.95, 21.43, 17.45. IR (CH_2Cl_2) 3416, 1651 cm^{-1} ; mp 187-193 °C; $[\alpha]_D^{24.3}$ = -27.77° (c 0.2, ethanol). Rt (Chiral HPLC) = 9.537 min.

Supplementary References

1. Weichu Xu.; George E. W.; Milka Y.; Ivan B. Y. Synthesis and Absolute Configuration Assignment of 9-Hydroxyrisperidone Enantiomers. *Letters in Organic Chemistry*, 11, 470-473 (2014).
