# Supplementary material for: Discovery of a peripheral 5HT2A antagonist as a clinical candidate for metabolic dysfunction-associated steatohepatitis
Source: Nat Commun. 2024 Jan 20;15:645. doi: 10.1038/s41467-024-44874-3 (PMC10799935; doi:10.1038/s41467-024-44874-3)
Supplement: Supplementary file 3 — Reporting Summary [file 41467_2024_44874_MOESM3_ESM.pdf]

nature portfolio

Corresponding author(s): Jin Hee Ahn, Hail Kim

Last updated by author(s): Dec 15, 2023

# Reporting Summary

Nature Portfolio wishes to improve the reproducibility of the work that we publish. This form provides structure for consistency and transparency in reporting. For further information on Nature Portfolio policies, see our Editorial Policies and the Editorial Policy Checklist.

## Statistics

For all statistical analyses, confirm that the following items are present in the figure legend, table legend, main text, or Methods section.

| n/a | Confirmed | |
|---|---|---|
| ☐ | ☒ | The exact sample size (*n*) for each experimental group/condition, given as a discrete number and unit of measurement |
| ☐ | ☒ | A statement on whether measurements were taken from distinct samples or whether the same sample was measured repeatedly |
| ☐ | ☒ | The statistical test(s) used AND whether they are one- or two-sided<br>*Only common tests should be described solely by name; describe more complex techniques in the Methods section.* |
| ☒ | ☐ | A description of all covariates tested |
| ☐ | ☒ | A description of any assumptions or corrections, such as tests of normality and adjustment for multiple comparisons |
| ☐ | ☒ | A full description of the statistical parameters including central tendency (e.g. means) or other basic estimates (e.g. regression coefficient) AND variation (e.g. standard deviation) or associated estimates of uncertainty (e.g. confidence intervals) |
| ☐ | ☒ | For null hypothesis testing, the test statistic (e.g. *F*, *t*, *r*) with confidence intervals, effect sizes, degrees of freedom and *P* value noted<br>*Give P values as exact values whenever suitable.* |
| ☒ | ☐ | For Bayesian analysis, information on the choice of priors and Markov chain Monte Carlo settings |
| ☒ | ☐ | For hierarchical and complex designs, identification of the appropriate level for tests and full reporting of outcomes |
| ☒ | ☐ | Estimates of effect sizes (e.g. Cohen's *d*, Pearson's *r*), indicating how they were calculated |

*Our web collection on statistics for biologists contains articles on many of the points above.*

## Software and code

Policy information about availability of computer code

| | |
|---|---|
| Data collection | QuantiStudio Software (Applied Biosystems) was used for realtime PCR data collection. Body composition were measured by Bruker LF50 BCA-analyzer. Confocal Lase Scanning Microscopes (Leica, SP8X and Zeiss, LSM 780)) with Leica Application Suite X V.3.7.1.2166 and ZEN blue V.3.1 software were used to acquire imaging data. |
| Data analysis | Statistics were calculated by Graphpad Prism 8, ImageJ V.1.52v for quantify immunofluorescence data, ZEN blue V.3.1 and Leica Application Suite X V3.7.1.21655 were used to analyze imaging data. |

For manuscripts utilizing custom algorithms or software that are central to the research but not yet described in published literature, software must be made available to editors and reviewers. We strongly encourage code deposition in a community repository (e.g. GitHub). See the Nature Portfolio guidelines for submitting code & software for further information.

# Data

Policy information about availability of data

All manuscripts must include a data availability statement. This statement should provide the following information, where applicable:

- Accession codes, unique identifiers, or web links for publicly available datasets
- A description of any restrictions on data availability
- For clinical datasets or third party data, please ensure that the statement adheres to our policy

The raw data generated in this study are provided as a Source Data file. Source data are provided with this paper. All data that support the findings of this study are available from the authors on reasonable request.

# Research involving human participants, their data, or biological material

Policy information about studies with human participants or human data. See also policy information about sex, gender (identity/presentation), and sexual orientation and race, ethnicity and racism.

| | |
|---|---|
| Reporting on sex and gender | Not Applicable |
| Reporting on race, ethnicity, or other socially relevant groupings | Not Applicable |
| Population characteristics | Not Applicable |
| Recruitment | Not Applicable |
| Ethics oversight | Not Applicable |

Note that full information on the approval of the study protocol must also be provided in the manuscript.

# Field-specific reporting

Please select the one below that is the best fit for your research. If you are not sure, read the appropriate sections before making your selection.

[x] Life sciences  [ ] Behavioural & social sciences  [ ] Ecological, evolutionary & environmental sciences

For a reference copy of the document with all sections, see nature.com/documents/nr-reporting-summary-flat.pdf

# Life sciences study design

All studies must disclose on these points even when the disclosure is negative.

| | |
|---|---|
| Sample size | No statistical methods were used to predetermine sample sizes. Sample size was chosen based on own previous results and according to standards in the field. Sample sizes were similar to those reported previously: e.g. doi: 10.1038/s41467-018-07287-7. |
| Data exclusions | No data were excluded from the analysis. |
| Replication | Results from cultured cell lines are based on experiments that have been independently performed at least 3 times with one or more technical replicates per experiment, as specified in the figure legends. All findings were reproduced. For the in vivo efficacy of compound 11c, every mouse represents a biological replicate (n), and the numbers are mentioned in each figure and/or figure legends. |
| Randomization | All of sex and age-matched mice were randomly assigned to each group. |
| Blinding | Total NAFLD activity scores were quantified in a blinded manner. Mice were randomized into the different groups. The investigators were blinded to group allocation during data collection and analysis. |

# Reporting for specific materials, systems and methods

We require information from authors about some types of materials, experimental systems and methods used in many studies. Here, indicate whether each material, system or method listed is relevant to your study. If you are not sure if a list item applies to your research, read the appropriate section before selecting a response.

## Materials & experimental systems

| n/a | Involved in the study | |
|---|---|---|
| ☐ | ☒ | Antibodies |
| ☐ | ☒ | Eukaryotic cell lines |
| ☒ | ☐ | Palaeontology and archaeology |
| ☐ | ☒ | Animals and other organisms |
| ☒ | ☐ | Clinical data |
| ☒ | ☐ | Dual use research of concern |
| ☒ | ☐ | Plants |

## Methods

| n/a | Involved in the study | |
|---|---|---|
| ☒ | ☐ | ChIP-seq |
| ☒ | ☐ | Flow cytometry |
| ☒ | ☐ | MRI-based neuroimaging |

# Antibodies

Antibodies used

α-SMA (catalog # ab5694, 1:500 dilution ), TNF-α (catalog # ab6671, 1:100 dilution) and IL-1β (catalog # ab9722, 1:100 dilution)

Validation

All immunofluorescence experiments were performed on mouse tissues in this study. All antibodies used in this study are commercially available and are stated to be tested by the manufacturer for species reactivity to mice. All antibodies were validated and described in our previous papers (Oh, C.J.; Kim, J.Y.; Min, A.K.; Park, K.G.; Harris, R.A.; Kim, H.J; Lee, I.K. Sulforaphane attenuates hepatic fibrosis via NF-E2-related factor 2-mediated inhibition of transforming growth factor-β/Smad signaling. Free Radical Biology and Medicine, 52(3), 671-682 (2012)).The statements and validation data for each antibody for the species and application are also available on the manufacturer's website.

# Eukaryotic cell lines

Policy information about cell lines and Sex and Gender in Research

Cell line source(s)

HEK293 were purchased from the American Type Culture Collection (ATCC, Manassas, VA, USA), Human 5HT2A-CMV6-vector was purchased from Origene (Cat. RC210182). This construct was transfected into HEK293 cells with Lipofectamin 3000 (Invitrogen; L3000015). The transfected cells were cultured in a medium containing 0.3 mg/mL G418 (Sigma; G418-RO) for 6 weeks, and single colonies were subsequently isolated and were utilized in experiments as described in the manuscript entitled "Design, Synthesis, and Biological Evaluation of New Peripheral 5HT2A Antagonists for Nonalcoholic Fatty Liver Disease." published in Journal of Medicinal Chemistry, 2020. The cell lines for the 5HT subtypes activity were performed by Eurofins Cerep, France (5HT1A, PMID: 15534042; 5HT1B, PMID: 9484854; 5HT1D, PMID: 8978753; 5HT2B and 5HT2C, PMID: 10498829; 5HT4E, PMID: 10683202; 5HT6, PMID: 8522988; 5HT7, PMID: 9808674). The cell line in table 5 for cytotoxic test, were performed by KRICT, Republic of Korea,

Authentication

The cell line has not been authenticated in the current study.

Mycoplasma contamination

All cells utilized were periodically tested for mycoplasma contamination using a mycoplasma detection kit (Cat. No. rep-pt1; InvivoGen), and all cell lines were tested negative for mycoplasma contamination.

Commonly misidentified lines
(See ICLAC register)

No commonly misidentified cell lines were used in this study.

# Animals and other research organisms

Policy information about studies involving animals; ARRIVE guidelines recommended for reporting animal research, and Sex and Gender in Research

Laboratory animals

All animal experiments were approved by the KAIST Institutional Animal Care and Use Committee (IACUC) and the Kyungpook National University IACUC. 5-week-old (the Jpan SLC. Inc.) and 11-week-old (the Charles River Japan) C57BL/6J male mice were used for all animal experiments CDAHFD and HFD, respecitively. Mice were maintained in a specific pathogen-free barrier facility under a regular light-dark cycle (12-hour light/12-hour dark) at 24℃ with 40-60% humidity. Food (standard chow diet, Envigo, 2018S) and water were provided ad libitum.

Wild animals

The study did not involve wild animals.

Reporting on sex

Only male mice were used for this study.

Field-collected samples

The study did not involve samples collected in the field.

Ethics oversight

All animal experiments were complied with relevant ethical regulations. Experimental protocols for this study were approved by the Institutional Animal Care and Use Committee at the Korea Advanced Institute of Science and Technology (KA2023-027-v2) and Kyungpook National University (KNU-20200134).

Note that full information on the approval of the study protocol must also be provided in the manuscript.
